# Extracellular Matrix Disparities in an *Nkx2-5* Mutant Mouse Model of Congenital Heart Disease

**DOI:** 10.3389/fcvm.2020.00093

**Published:** 2020-05-29

**Authors:** Deanna Bousalis, Christopher S. Lacko, Nora Hlavac, Fariz Alkassis, Rebecca A. Wachs, Sahba Mobini, Christine E. Schmidt, Hideko Kasahara

**Affiliations:** ^1^J. Crayton Pruitt Family Department of Biomedical Engineering, University of Florida, Gainesville, FL, United States; ^2^Department of Physiology and Functional Genomics, University of Florida, Gainesville, FL, United States; ^3^Department of Biological Systems Engineering, University of Nebraska-Lincoln, Lincoln, NE, United States; ^4^Instituto de Micro y Nanotecnología, IMN-CNM, CSIC (CEI UAM+CSIC), Madrid, Spain; ^5^Centro de Biología Molecular Severo Ochoa (CBMSO, UAM-CSIC), Universidad Autónoma de Madrid, Madrid, Spain

**Keywords:** extracellular matrix, congenital heart disease, decellularization, proteomics, *Nkx2-5*, integrin, gap junction, connexin

## Abstract

Congenital heart disease (CHD) affects almost one percent of all live births. Despite diagnostic and surgical reparative advances, the causes and mechanisms of CHD are still primarily unknown. The extracellular matrix plays a large role in cell communication, function, and differentiation, and therefore likely plays a role in disease development and pathophysiology. Cell adhesion and gap junction proteins, such as integrins and connexins, are also essential to cellular communication and behavior, and could interact directly (integrins) or indirectly (connexins) with the extracellular matrix. In this work, we explore disparities in the expression and spatial patterning of extracellular matrix, adhesion, and gap junction proteins between wild type and *Nkx2-5*^+/*R*52*G*^ mutant mice. Decellularization and proteomic analysis, Western blotting, histology, immunostaining, and mechanical assessment of embryonic and neonatal wild type and *Nkx2-5* mutant mouse hearts were performed. An increased abundance of collagen IV, fibronectin, and integrin β-1 was found in *Nkx2-5* mutant neonatal mouse hearts, as well as increased expression of connexin 43 in embryonic mutant hearts. Furthermore, a ventricular noncompaction phenotype was observed in both embryonic and neonatal mutant hearts, as well as spatial disorganization of ECM proteins collagen IV and laminin in mutant hearts. Characterizing such properties in a mutant mouse model provides valuable information that can be applied to better understanding the mechanisms of congenital heart disease.

## Introduction

Congenital heart disease (CHD) affects nine out of every 1,000 children born worldwide (1, 2), or almost one percent of all live births. CHD is characterized by a variety of cardiac anomalies (e.g., atrial or ventricular septal defect, patent ductus arteriosus, pulmonary valve stenosis), which can affect blood flow through the heart as a result of holes between chambers or thickened valves (3). While there have been many diagnostic and surgical advancements in detecting and repairing cardiac defects in newborns or fetuses, the causes and mechanisms of these cardiac maldevelopments are still primarily unknown and require further exploration.

The *Nkx2-5* transcription factor is one of the earliest cardiogenic markers expressed during embryonic heart development (4). Inherited mutations of the *Nkx2-5* gene are a common genetic cause of CHD in humans as they can result in an assortment of cardiac malformations, such as right isomerism, atrioventricular (AV) block, ventricular septal defect (VSD), and atrial septal defect (ASD) (5–9). Over 50 *Nkx2-5* mutations have been identified that result in congenital heart defects, many of which have been represented in mutant mouse models (6, 10–12). We have previously developed a heterozygous knock-in mutant mouse model with missense mutation 52Arg → Gly (R52G) in the *Nkx2-5* transcription factor homeodomain (13). Mutant mouse hearts showed varying disease phenotypes among samples, including Ebstein malformation and ventricular and atrial septal defect, and all hearts demonstrated a ventricular noncompaction phenotype. Ventricular noncompaction is a condition in which the trabecular layer of the heart persists into the ventricular walls, causing a spongy rather than compact morphology and in turn limiting the heart's ability to contract and pump blood effectively (9, 14).

The extracellular matrix (ECM) and its remodeling throughout development has been suggested to play a role in ventricular compaction (15) as well as development of other regions of the heart. For example, differential ECM profiles exist throughout postnatal aortic valve maturation in mice (16). Other research has shown some ECM irregularities between diseased and healthy cardiac conditions (17, 18). Increased production and disorganization of aortic valve cushion ECM has been reported in a congenital aortic valve stenosis mouse model (19). Additionally, irregular quantities of ECM components such as hyaluronan may lead to altered cellular behavior and downstream congenital heart defects (20). It is currently unknown whether there is a relationship between the *Nkx2-5* genetic mutation, cardiac ECM, and subsequent heart development. However, matrix composition and stiffness have been suggested to alter embryonic development (21, 22). Furthermore, as the ECM is integral to cell communication, function, and differentiation (23), it could likely play a role in disease development and pathophysiology.

Integrins and gap junction proteins also play a large role in cardiac development, during which they possess dynamic spatiotemporal profiles (17, 24–27). Integrins are transmembrane proteins that mediate cell-matrix interactions by serving on the cell surface as receptors for ECM ligands while connecting to the cytoskeleton and various signaling cascades on the cell interior (25). Modifications of adhesion-related proteins may cause ECM irregularities. For example, removal of integrin-linked-kinase, a protein that binds to beta integrins at focal adhesion complexes, from the neural crest of murine hearts resulted in outflow tract malformations and ventricular septal defects, as well as reduced expression of ECM proteins (28). Cardiac gap junction proteins, such as connexins, allow for ion and small molecule transfer, which is essential for electrical conduction and muscle contraction in the heart (27). Research has suggested some connection between connexin 43 mutations and cardiac malformations (27), and interestingly, between *Nkx2-5* mutations and connexin expression (29). However, it is likely that connexins are not solely the cause of cardiac malformations, and that a more complex cascade of cell-cell and cell-ECM interactions play a role in such development (27). Regardless, there is enough evidence to elicit further exploration into the presence of gap junction protein and integrin irregularities in congenital heart disease.

In this work, we explore disparities in the expression and spatial patterning of extracellular matrix, adhesion, and gap junction proteins between wild type and *Nkx2-5*^+/*R*52*G*^ mutant mice. Awareness of such trends allows us to better understand how key ECM, integrin, and gap junction proteins are differentially expressed in diseased hearts, and elicits further research into their roles in the mechanisms of congenital heart disease development and pathophysiology.

## Materials and Methods

### Animal Models and Tissue Harvest

All animal experiments were performed with approval from the Institutional Animal Care and Use Committee at the University of Florida. 129/SvPasCrl mice were utilized in these studies (Charles River Laboratories, Wilmington, MA) for wild type samples. A subset of the mice were backcrossed with mutant *Nkx2-5*^+/*R*52*G*^ knock-in mice [described previously (13)] for at least 10 generations. *Nkx2-5*^+/*R*52*G*^ male mice were bred with female wild type mice. To determine embryonic staging, mice were plug-checked daily; the morning the vaginal plug was found was considered embryonic day 0.5 (E0.5). On day E13.5, mothers were sacrificed, and embryonic hearts were dissected for histological analysis or Western blotting. Hearts were also harvested from postnatal day 1 (P1) mice on the day of delivery.

### Decellularization Process

A diffusion method of chemical decellularization, adapted from previously published methods (30, 31), was employed. Embryonic hearts were placed in 2 mL low retention tubes (Fisher Scientific 02-681-321), submerged with detergent and/or buffer, and placed on a rotator at 15 rpm (Thermo Fisher) for the following durations. Hearts were washed with ddH_2_O for 8 h, sulfobetaine-10 (SB-10, Sigma Aldrich D4266) for 4 h, 100 mM Na/50 mM phosphate-buffered saline (PBS) for 15 min, 5% sodium deoxycholate (SD, Sigma Aldrich D6750)/sulfobetaine-16 (SB-16, Sigma Aldrich H6883) for 4 h, 100 mM Na/50 mM PBS for three 15-min periods, SB-10 for 1.75 h, 100 mM Na/50 mM PBS for 15 min, 5% SD/SB-16 for 3 h, 50 mM Na/10 mM PBS for two 15-min periods, deoxyribonuclease I (DNase I, Sigma-Aldrich D4527) solution for 12 h (with no rotation), and 50 mM Na/10 mM PBS for 15 min. Decellularized hearts, in fresh 50 mM Na/10 mM PBS solution, were stored at −80°C until further use.

### Mass Spectrometry and Proteomic Analysis

A detailed methodology can be found in the Supplementary Material. Briefly, each E13.5 embryonic mouse heart was submerged in 25 μL RIPA buffer (Millipore Sigma 20-188) containing Halt™ protease and phosphatase inhibitor cocktail (Thermo Scientific Pi78444) and phenylmethylsulfonyl fluoride (PMSF, Sigma Aldrich 10837091001) and sonicated to homogenize into a lysate. To accurately compare between unprocessed and decellularized hearts, and to account for mass and intracellular protein loss during the decellularization process, samples were normalized per whole heart. Lysate samples were submitted to the UF Mass Spectrometry Research and Education Center for processing and analysis. Scaffold (version 4.9.0, Proteome Software Inc., Portland, OR) was used to validate MS/MS based peptide and protein identifications. Peptide identifications were accepted if they could be established at >95.0% probability by the Peptide Prophet algorithm (32) with Scaffold delta-mass correction. Protein identifications were accepted if they could be established at >99.0% probability and contained at least one identified peptide. Protein probabilities were assigned by the Protein Prophet algorithm (33).

### Western Blotting

For both embryonic and neonatal hearts, each heart was immersed in RIPA lysis buffer, as described above, and sonicated to homogenize the sample. After a 15-min incubation on ice, tissue lysates were centrifuged to pellet any insoluble material. Supernatants were collected and total protein content was measured using a Pierce 660 Protein Assay (Thermo Fisher Scientific 22660). For neonatal hearts, SDS-PAGE was performed on samples, and proteins were transferred to 0.22 μm PVDF membranes (Bio-Rad Immun-Blot 1620177). Membranes were blocked in Odyssey Blocking Buffer (LI-COR 927-50000) for an hour, incubated in primary antibody solution overnight at 4°C, washed in tris-buffered saline-Tween 20 solution (TBS-T), incubated in secondary antibody solution for 2 h at room temperature, washed, and imaged using a LICOR Odyssey CL-X. Primary antibodies were diluted in Odyssey Blocking Buffer with 0.2% Tween 20 (Fisher Bioreagents BP337-100) at the following ratios: fibronectin (Abcam ab2413) 1:500, laminin (Sigma L9393) 1:500, collagen IV (Sigma 4200500) 1:500, GAPDH (Abcam ab8245) 1:2000. Secondary antibodies were either goat anti-rabbit IgG IRDye 680RD (LI-COR 926-68071) or goat anti-mouse IgG IRDye 800CW (LI-COR 926-32210) diluted 1:10,000 in Odyssey Blocking Buffer with 0.01% SDS (Bio-Rad 1610301) and 0.2% Tween 20.

Because of the small size and limited sample quantity available from embryonic hearts, a ProteinSimple Wes capillary-based system (Biotechne) was used in lieu of traditional Western blotting, as it requires less total protein per assay (34). The Wes system was also used for experiments in which target proteins were generally of lower abundance to enhance signal detection (for example, integrin β-1). Samples were prepped according to ProteinSimple Wes recommended protocols. For detection of laminin, collagen IV, fibronectin, integrin β-1, integrin α-5, and lamin B1, samples were run on a 66-440 kDa Jess/Wess separation module (ProteinSimple, Biotechne, USA SM-W008). For detection of connexin 43 and GAPDH, samples were run on a 12-230 kDa separation module (ProteinSimple, Biotechne, USA SM-W004). Primary antibody dilutions and sample concentrations are as follows: laminin 1:100, 0.25 μg/μL sample loaded; collagen IV (Abcam ab6586) 1:100, 1.15 μg/μL sample loaded; fibronectin 1:100, 0.005 μg/μL sample loaded; lamin B1 (Cell Signaling Technologies 13435S) 1:100, 1.15 μg/μL sample loaded; GAPDH 1:100, 0.9 μg/μL sample loaded; integrin β-1 (Novus NBP2-16974), 1:100, 1.45 μg/μL for embryonic, 1.8 μg/μL for neonatal loaded; Connexin 43 (Cell Signaling Technologies 3512S) 1:100, 0.45 μg/μL sample loaded; integrin α-5 (Cell Signaling Technologies 4705T) 1:100, 0.2 μg/μL sample loaded.

For traditional Western blotting analysis, LICOR Image Studio Lite software was used to detect band intensities. For capillary-based Westerns, chemiluminescence intensity electropherograms were plotted and analyzed using ProteinSimple's Compass SW software. The area under each curve peak corresponding to the protein's molecular weight was recorded for every sample, and these values were used for statistical comparisons. A standardized exposure time and peak fitting size was used for all of the samples per target protein. For collagen IV Western blot quantification, the prominent bands at 250 kDa and directly below were measured for both traditional and capillary Westerns. Depending on the target protein molecular weight, either lamin B1 or GAPDH was used as a housekeeping protein for normalization (35). To ensure the housekeeping protein expressions did not significantly differ between mutant and wild type hearts, a traditional Western blotting experiment was performed with neonatal heart samples in which membranes were stained for total protein with Ponceau S (Sigma-Aldrich P7170) after transfer, and then probed for both GAPDH (1:2000 dilution) and Lamin B1 (1:500 dilution). GAPDH and Lamin band intensities were quantified and normalized to total protein band intensities (Supplementary Figure 4). No significant differences between neonatal wild type and mutant hearts were detected, hence validating our use of these housekeeping proteins for normalization of target proteins of interest.

### Immunohistochemistry and Other Histological Assessments

Hearts were fixed in 4% paraformaldehyde, soaked in sucrose solution overnight, embedded in OCT Tissue-Tek (Electron Microscopy Sciences 62550-12), and placed at 4°C overnight. Hearts were all placed in the same orientation immediately prior to freezing at −80°C, and cryosectioned into 10-μm sections parallel to the coronal plane, along the sagittal plane, using a Leica CM 1950 Cryostat. Sections in which all four chambers of the heart were visible were used for immunostaining and histological assessment. Slides were warmed at 37°C for 2 h, incubated with blocking buffer [3% goat serum, (Sigma G9023), 0.3% Triton X-100 (Sigma 93443) in 1X PBS] for an hour, incubated in primary antibody solution overnight, then secondary antibody solution overnight, and then DAPI for 10 min, with 1X PBS washes in between each incubation step. Primary antibody solutions consisted of collagen IV (Abcam ab6586) and laminin at 1:200 dilutions in blocking buffer. Secondary antibody solution consisted of goat anti-mouse IgG Alexa Fluor 488 (Thermo Fisher A-11001), goat anti-rabbit IgG Alexa Fluor 647 (Abcam ab150079), or goat anti-mouse IgG Alexa Fluor 568 (Thermo Fisher A-11011) at 1:500 dilution in blocking buffer. Coverslips (1.5 mm, Erie Scientific) were mounted on each slide using Fluoromount-G slide mounting medium (Thermo Fisher OB100-01) and left to dry overnight. Slides were imaged using a Zeiss Axioimager Z2 or a Zeiss 880 Confocal Laser Scanning Microscope.

Neonatal laminin immunohistochemical images were semi-quantified using ImageJ software. Three to five rectangular regions of interest (ROIs), all of the same size (15,000 μm^2^), were assigned in each section. Regions were placed along the ventricular walls because this is where the ventricular non-compaction phenotype of interest persists. Then, all the enclosed pores present in the staining were traced in each region of interest. Please refer to Supplementary Figure 2 for a visual example of analysis. Authors were blinded to the identification of the ROIs during pore tracing. The area and circularity values were obtained for each of the pores traced. Additionally, an ellipse was fit to each pore, and its central angle relative to the horizontal axis was recorded. These data were averaged for each ROI, which were then averaged per heart sample. To obtain angle deviation calculations, the standard deviation values per ROI were obtained for the central angles of fitted ellipses. The average “deviation” value was used as a metric for pore alignment.

For hematoxylin and eosin staining, embryonic hearts were fixed, embedded in paraffin, and sectioned into 5 μm sections using a microtome. Neonatal hearts were processed as described above and cryosectioned. Slides with paraffin sections were deparaffinized and underwent a sequence of rehydration in aqueous ethanol solutions. Cryosections were fixed in 80% methanol and incubated in PBS to remove OCT. Both types of sections underwent washes in Harris hematoxylin (Sigma-Aldrich HHS16), water, bluing solution (Leica 3802915), water, defining solution (Leica 3803590), 80% ethanol, eosin Y (Electron Microscopy Sciences 26051-10), 95 and 100% ethanol, and xylenes (Avantor 8668-16).

### Statistical Analysis

Power analyses were performed using JMP Pro software to determine the sample sizes required to ensure that claims of statistical significance were sufficiently powered (80 percent or higher). The only experiment not sufficiently powered is the neonatal fibronectin Western blot (*n* = 3 per group) because we were unable to obtain a sufficient number of samples for extraneous reasons. All experiments were performed with at least *n* = 3 biological replicates (i.e., individual hearts) per group, and the exact number is specified in each figure caption. When comparing between two groups, a student's *t*-test was performed using GraphPad Prism and significance was considered a two-tailed *p* ≤ 0.05. If data from the two groups possessed unequal variances, a *t*-test with Welch's correction was performed using GraphPad Prism and significance was considered a two-tailed *p* ≤ 0.05. For proteomic analysis, significance threshold is described in the appropriate section that accounts for false detection from multiple comparisons.

## Results

### Decellularization Creates ECM-Rich Hearts for Proteomic Assessment

**Figure 1:**
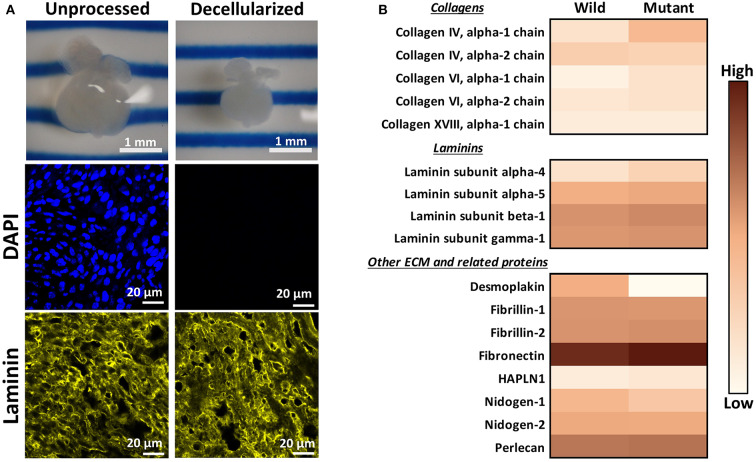
Decellularization creates ECM-rich hearts for proteomic assessment. **(A)** Top: Image of the same embryonic heart before and after decellularization. Middle: DAPI staining demonstrating removal of nuclear materials from tissue post-decellularization. Bottom: Immunostaining demonstrating preservation of laminin throughout the decellularization process. DAPI and Laminin scale bars: 20 μm. Images shown are representative of three biological replicates. Laminin staining used Alexa Fluor 647 dye but was pseudo-colored yellow for ease of comparison across graphics. **(B)** Heat map displaying ECM proteins detected in mass spectrometry analysis with at least one average spectral count per group. Color is based on the average spectral count value of three biological replicates.

An initial proteomic assessment was performed on E13.5 embryonic wild type and *Nkx2-5* mutant mice as a discovery method to identify proteins of interest that differed between the two groups. As ECM proteins were of greatest interest in this study, hearts were decellularized to remove intracellular content and to generate ECM-rich hearts. Analysis of decellularized tissue, rather than unprocessed tissue, allows for better proteomic detection of ECM proteins, since highly abundant cellular proteins (e.g., myosin, actin) can mask the signal of generally lesser-abundant ECM proteins (36, 37). Embryonic hearts underwent a novel method of diffusion-based decellularization [adapted from Hudson et al. (30) and McCrary et al. (31)] utilizing anionic detergent sodium deoxycholate, zwitterionic detergents sulfobetaine-10 and−16, and enzyme deoxyribonuclease (DNase). Immunostaining was used to confirm removal of intracellular content and preservation of extracellular matrix proteins. Figure 1A shows that the heart shrinks and loses its initial pinkish hue after decellularization, demonstrating the loss of cellular content. Additionally, no DAPI staining was detected in decellularized tissue, implying that no nuclear content remained after processing. Finally, laminin immunostaining revealed comparable staining intensities between unprocessed and decellularized tissue. After these preliminary assessments confirmed intracellular content removal and preservation of extracellular matrix proteins, decellularized hearts were homogenized, and submitted for mass spectrometry and proteomic analysis.

Through mass spectrometry, 1,324 total proteins were detected, with 35 being ECM or ECM-related proteins. Figure 1B displays the relative abundance of common ECM and ECM-related proteins, which had at least one average spectral count (out of biological triplicates) detected. The full table of detected proteins with at least one average spectral count is provided in Supplementary Table 1, with the *p*-values of each comparison. The majority of detected ECM proteins appeared to be more abundant in mutant tissue, with the exception of desmoplakin, fibrillin-1, nidogen-1 and - 2, and collagen IV alpha 2 chain. The proteomic data were used primarily as a preliminary “discovery” method to identify proteins of interest, which were further validated with other quantitative and semi-quantitative methods. As most laminins and collagens, as well as fibronectin, were more abundant in mutant hearts according to these data, we focused on laminin, collagen IV, and fibronectin for further assessments.

### Compositional ECM Differences in Wild Type and *Nkx2-5* Mutant Hearts

**Figure 2:**
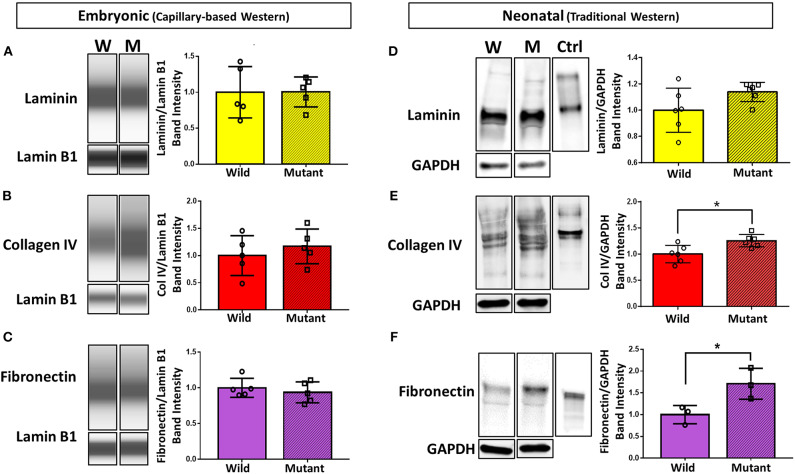
Western blotting detects significant differences in collagen IV and fibronectin at the neonatal age, but no differences in ECM expression between embryonic *Nkx2-5* mutant and wild type mouse hearts. For embryonic heart samples: ECM capillary-based Western band images for embryonic heart samples (left) and subsequent quantification (right) for laminin **(A)**, collagen IV **(B)**, and fibronectin **(C)**. Each lane displays a representative “pseudoblot” band for each group, with *n* = 5 for each group. Each target ECM protein band intensity was normalized to that sample's lamin B1 intensity. For neonatal heart samples: traditional Western blotting representative bands (left) of **(D)** laminin (*n* = 12 total), **(E)** collagen IV (*n* = 12 total), and **(F)** fibronectin (*n* = 6 total) and corresponding quantifications (right). Control (“Ctrl”) lane shows each respective purified protein probed with the same primary antibody as the samples. Each target ECM protein band intensity was normalized to that sample's GAPDH intensity. Graphs display mean ± standard deviation of ECM band intensities normalized to housekeeping protein, relative to wild type group. Significance *p < 0.05. W, wild type; M, mutant.

To further explore differential expression of ECM proteins of interest (based on preliminary proteomics results), Western blotting was performed on E13.5 wild type and mutant hearts for laminin (all subunits), collagen IV (all subunits), and fibronectin (all subtypes). Due to the small size of embryonic heart samples, an automated capillary-based Western blotting method was used to preserve protein and sample. Chemiluminescence intensity profiles of each protein were plotted and analyzed (Figures 2A–C). Expression of laminin, collagen IV, and fibronectin did not significantly differ between groups. Samples were all probed for housekeeping protein lamin B1 in addition to proteins of interest. During analysis, the ECM protein band intensities were normalized to each sample's respective lamin B1 band intensity to account for any sample prep and/or loading variations.

Western blotting was also performed on neonatal (postnatal day 1, P1) wild type and mutant mouse hearts to demonstrate whether trends in ECM differences vary temporally (Figures 2D–F). Trends remained consistent for laminin, where no difference was detected between mutant and wild type samples. Collagen IV, although not significantly different at the embryonic stage, showed a statistically significant 25 percent increase in neonatal mutant hearts compared to neonatal wild type hearts. Furthermore, fibronectin was expressed 71 percent more in neonatal mutant hearts than wild type hearts. Housekeeping protein GAPDH was used for normalization.

### Gross Morphological Differences and Visualization of ECM Distribution and Patterning

**Figure 3:**
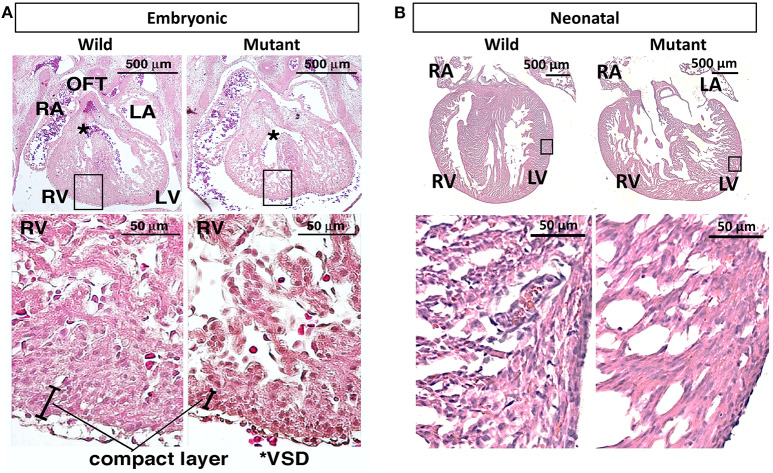
Hematoxylin and eosin staining demonstrates ventricular noncompaction phenotype at both embryonic and neonatal ages in mutant *Nkx2-5* mouse hearts **(A)** Embryonic heart and **(B)** neonatal heart hematoxylin and eosin staining demonstrating the ventricular noncompaction phenotype characteristic of mutant hearts. OFT, outflow tract; RA, right atrium; LA, left atrium; RV, right ventricle; LV, left ventricle; *VSD, ventricular septal defect.

It has been previously published that postnatal *Nkx2-5* mutant mouse hearts possess hypertrabeculations due to ventricular noncompaction phenotype (13). Figure 3A demonstrates that such phenotype is apparent in embryonic as well as neonatal mutant hearts (Figure 3B). It was assumed that mutant hearts may possess different mechanical properties than their wild type counterparts because of this noncompact, spongy morphology. Neonatal heart mechanical properties were determined using indentation. No significant differences in mechanical properties were detected between wild type and mutant hearts. More information and results can be found in Supplementary Figure 1.

**Figure 4:**
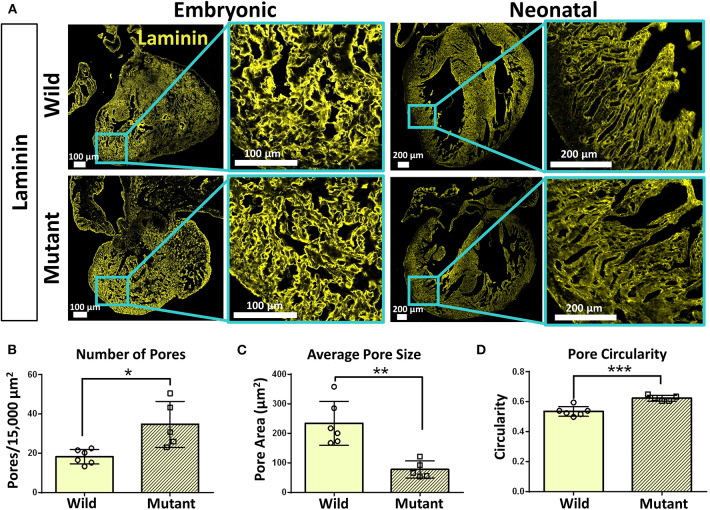
Laminin immunostaining reveals organizational differences between wild type and *Nkx2-5* mutant ECM. **(A)** Laminin (yellow) immunostaining on both embryonic and neonatal wild type and *Nkx2-5* mutant mouse hearts. Embryonic scale bars: 100 μm. Neonatal scale bars: 200 μm. Laminin staining used Alexa Fluor 647 dye but was pseudo-colored yellow for ease of comparison across graphics. **(B)** Average number of pores measured per 15,000 μm^2^ area of tissue. **(C)** Measure of average pore size in μm^2^ across neonatal wild type and mutant laminin staining. **(D)** Measure of average circularity of neonatal pores, where a circularity of “1” is a perfect circle. For quantification, *n* = 6 for wild type and *n* = 5 for mutant hearts. Graphs show mean ± standard deviation with significance *p < 0.05, **p < 0.01, and ***p < 0.001.

Immunostaining was performed on embryonic and neonatal wild type and *Nkx2-5* mutant hearts for laminin and collagen IV to assess any spatial or organizational pattern differences. Particular attention was given to the ventricular wall regions of the hearts, where *Nkx2-5* mutant mice possess the ventricular noncompaction phenotype (13). Since trabeculations are known to persist in mutant hearts compared to wild type hearts, these areas of interest were viewed at higher magnifications. Laminin does not appear to differ greatly between wild type and mutant embryonic hearts, while differences are apparent at the neonatal age (Figure 4A), where the laminin forms shorter, more diffuse networks in mutant neonatal hearts compared to their wild type counterpart. Further, collagen IV appears to be less spatially organized in mutant hearts of the neonatal age compared to wild type hearts (Supplementary Figure 2A). To quantitatively assess such disorganization, the area, circularity, and alignment of pores visualized by laminin staining along the ventricular walls of wild type and *Nkx2-5* mutant hearts were measured. Mutant hearts had a significantly greater number of pores per given area (Figure 4B) and the average pore size of mutant hearts was less than three times that of wild type hearts (Figure 4C). Additionally, mutant laminin pores had a significantly greater circularity value (on a scale of 0–1, where a value of 1 represents a perfect circle) (Figure 4D). The smaller, more abundant round pores are indicative of less organization of the ECM compared to the fewer elongated and larger pores apparent in the wild type hearts. Additionally, although it appears from some images that wild type laminin pores are also more uniformly aligned in a given direction, when quantifying how much the pore angles in each region of interest deviate from each other, we did not see any significant differences between the two genotypes (Supplementary Figure 2C).

### Adhesion and Gap Junction Protein Expression

**Figure 5:**
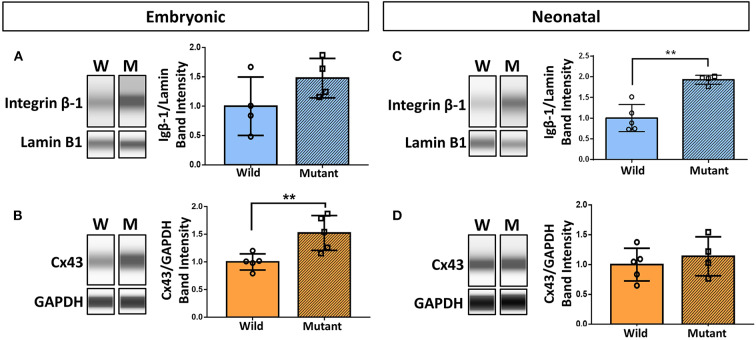
Western blotting demonstrates increased integrin β-1 and connexin 43 expression in neonatal and embryonic mutant *Nkx2-5* hearts, respectively. Capillary-based Western botting images and corresponding quantifications of embryonic wild type and mutant **(A)** integrin β-1 and **(B)** connexin 43 (Cx43), and neonatal wild type and mutant **(C)** integrin β-1 and **(D)** connexin 43. Graphs display mean ± standard deviation of protein band intensities normalized to either lamin B1 or GAPDH housekeeping proteins. Significance **p < 0.01. *n* = 8 total for embryonic and 9 total for neonatal integrin β-1 Westerns. *n* = 10 total for embryonic and 9 total for neonatal connexin 43 Westerns.

Results demonstrating differential abundance of collagen IV and fibronectin in wild type and *Nkx2-5* mutant neonatal hearts motivated interest to explore expression of cell adhesion molecule integrin β-1, which binds to both of these ECM proteins, and integrin α-5, which dimerizes with integrin β-1 to serve as a fibronectin receptor. Connexin 43 (also known as gap junction protein alpha-1), although not directly in contact with the ECM, also plays an essential role in cardiac cell communication, conduction, and contraction (38). Western blotting of these proteins demonstrated a 52 percent statistically significant increase in connexin 43 expression in mutant hearts compared to wild type hearts at the embryonic age (Figures 5B,D). This trend does not persist in neonatal mice, where there are no differences in connexin 43 expression between the two genotypes. Integrin β-1, however, possesses the opposite trend, where no differences are detected between mutant and wild type embryonic hearts, however a 93 percent statistically significant increase in expression is seen in mutant neonatal mouse hearts (Figures 5A,C). Western blotting for integrin α-5 expression in neonatal wild type and *Nkx2-5* mutant hearts did not reveal any significant differences between the two genotypes (Supplementary Figure 3).

## Discussion

One focus of this study was to characterize disparities in the ECM of wild type and *Nkx2-5* mutant mouse hearts. Although this study was not a comprehensive investigation into all potential ECM disparities that may exist, we chose three key ECM proteins, collagen IV, laminin, and fibronectin, to explore based on discovery mass spectrometry data. Two different ages were assessed (embryonic and neonatal), which provided insight into potential ECM dynamics throughout disease development. For target proteins, no significant differences in ECM expression were found until the neonatal age, at which point mutant hearts showed increased expression of collagen IV and fibronectin. Collagen IV is a major component of the basement membrane, which is essential for maintaining tissue structure and regulating cardiac contractility (39). Fibronectin is a structural glycoprotein that can influence cell behavior through its contact with both the basement membrane, fibrillar collagen network, and integrin receptors (40, 41). Fibronectin-integrin interactions can lead to downstream signaling cascades that control cytoskeleton reorganization and cell spreading, migration, and proliferation (40); thus, it is likely that ECM irregularities have some indirect link to cardiac malformations, such as the ventricular noncompaction phenotype seen in these studies.

Hematoxylin and eosin staining demonstrated that similar phenotypes exist in both embryonic and neonatal *Nkx2-5* mutant hearts. Although it has previously been demonstrated that neonatal mutant hearts possess ventricular noncompaction phenotypes (13), it has not been shown before that this phenotype takes form at or before the E13.5 stage. This is feasible, as trabeculation is thought to begin at embryonic day 9–9.5 in mice (42). Furthermore, our histology and ECM immunostaining data agree with previous research on ventricular noncompaction (15), with greater disorganization present in the compact and trabecular layers of the heart. Interestingly, although laminin was not differentially abundant between the two genotypes, immunostaining quantification demonstrated less spatial organization of laminin in neonatal *Nkx2-*5 mutant hearts. This indicates that ECM spatial arrangements can differ despite no change in overall abundance of the ECM protein. While other groups have explored the mechanisms of Notch and Nrg1 signaling in trabeculation (15), there is little existing research regarding the effect of *Nkx2-5* mutations on trabeculation during cardiac development. Here, we have shown that there is some connection between the two.

Assessments of adhesion proteins (integrin β-1 and α-5) and a gap junction protein (connexin 43) were also performed through Western blotting to obtain insight on upstream or downstream effects of ECM irregularities. Integrin β-1 was significantly overexpressed in neonatal mutant mouse hearts, whereas no significant differences were detected at the embryonic age. Integrin β-1 can form a dimer with α integrins 1-8, 10-11, and v to serve as ligands for laminin, collagen, and fibronectin (24, 43); therefore, it is fitting that these data correspond to the significant increase in collagen IV and fibronectin in neonatal mutant mouse hearts. Although integrin β-1 expression was increased in mutant hearts, no significant differences in expression of integrin α-5 were found between genotypes. This differential expression may be due to the specific functional roles that β-1 and α-5 have in cardiac development and myocyte function (44, 45). Integrin β-1 is known to form at least 12 different heterodimers with various α integrins, whereas integrin α-5 only binds to β-1 (43). Furthermore, there are multiple other integrin heterodimers that serve as fibronectin receptors, including α_v_β_1_, α_4_β_1_, and α_9_β_1_ (43, 46), thus it cannot be assumed that increased abundance of fibronectin would linearly correlate specifically to abundance of integrin α-5.

On the contrary, connexin 43 was significantly increased in embryonic mutant mouse hearts, with no trends apparent at the neonatal stage. Interestingly, connexin 43 is primarily present in trabeculated areas of the ventricle during embryonic development until around E15.5, after which point it becomes present throughout the entirety of the ventricular walls (47, 48). Overexpression of connexin 43 can also lead to heart defects such as right ventricle enlargement and non-compaction, trabecular disorganization, conotruncal region enlargement, and outflow tract abnormalities (49). Although the findings of Coppen et al. (48) and Ewart et al. (49) were not related to *Nkx2-5* mutations, they align with the ventricular noncompaction phenotype present in our E13.5 *Nkx2-5* mutant mouse heart samples. Furthermore, as connexin 43 holds major roles in cell signaling, mechanotransduction, ion exchange, and conduction (47, 50), its differential abundance in embryonic hearts potentially has both direct and indirect effects on development. Hence, there are likely indirect relationships between increased connexin 43 expression and downstream ECM production that require further exploration.

It should be noted that some limitations to the assessments performed in this study exist. For example, although tandem mass spectrometry is a powerful tool for proteome characterization, its analysis is restricted to proteins solubilized during tissue homogenization (51). As some ECM proteins are difficult to solubilize, it is possible that some proteins were lost in the sample preparation process. This could be attributed to why other common types of collagen, such as type 1 collagen, were not detected in this study. For this reason, proteomic analysis was used as a qualitative discovery tool, and proteins of interest were selected for further investigation with other methods. Regardless, Western blotting results were aligned with proteomic data, which suggested increased amounts of collagens, laminin, and fibronectin in mutant hearts. Although mass spectrometry data for embryonic hearts aligned more with the neonatal rather than embryonic Westerns, there were no contradicting proteomic and Western blotting data—either no significant differences were detected, or there was significantly increased ECM expression in mutant hearts. This could be attributed to sensitivity differences of the two techniques. Furthermore, perhaps ECM deposition increased over the course of development as a mechanism to provide cells with structural support to counteract the noncompaction phenotype sponginess, at which point the ECM amount and expression difference could be large enough for detection through Western blotting.

While we have investigated three select ECM proteins, there is a need for a more thorough investigation into other potential ECM differences, particularly in fibrillar collagen and lesser abundant ECM proteins not detected in this study. In the future, recently developed ECM solubilization techniques (51, 52), quantitative mass spectrometry methods (53, 54), and targeted mass spectrometry (55) could be applied to obtain more precise details about the ECM composition of wild type and mutant hearts. Additionally, other target proteins could be selected from our preliminary mass spectrometry assessment for further exploration via Western blotting. For example, desmoplakin was not detected in mutant mouse hearts at all, whereas it was present in wild type hearts, and it has also been associated with cardiomyopathies (56).

In summary, we report interesting trends in collagen IV, laminin, fibronectin, and ECM-related proteins of wild type and *Nkx2-5* mutant mouse hearts. This study provides information regarding morphological differences, mechanical properties, ECM spatial distribution, and relative abundance of ECM, connexin, and adhesion proteins in wild type and mutant hearts. One possible application for characterizing such properties in a mouse model would be replicating these parameters in 3D hydrogel ECM-based tissue mimics that serve as scaffolds for healthy and diseased cells. Applying different stimuli or screening therapeutic strategies could allow for greater mechanistic insight of congenital cardiac anomalies.

## Data Availability Statement

All datasets generated for this study are included in the article/Supplementary Material.

## Ethics Statement

The animal study was reviewed and approved by University of Florida Institutional Animal Care and Use Committee.

## Author Contributions

DB designed and performed experiments and prepared the manuscript. CL and NH assisted with experiment planning and execution. FA assisted with experiments. RW assisted in project conception and experimental design. SM assisted in experimental design. CS and HK provided experimental insight and edited manuscript.

## Conflict of Interest

The authors declare that the research was conducted in the absence of any commercial or financial relationships that could be construed as a potential conflict of interest.
