# Supplementary material for: Extracellular Matrix Disparities in an Nkx2-5 Mutant Mouse Model of Congenital Heart Disease
Source: Front Cardiovasc Med. 2020 May 29;7:93. doi: 10.3389/fcvm.2020.00093 (PMC7272573; doi:10.3389/fcvm.2020.00093)
Supplement: Supplementary file 1 [file Data_Sheet_1.docx]

Supplementary Material

# Supplementary Data

**Mass spectrometry and proteomic analysis: extended methodology.**

Each E13.5 embryonic mouse heart was submerged in 25 µL RIPA buffer (Millipore Sigma 20-188) containing Halt^TM^ protease and phosphatase inhibitor cocktail (Thermo Scientific Pi78444) and phenylmethylsulfonyl fluoride (PMSF, Sigma Aldrich 10837091001) and sonicated to homogenize into a lysate. To accurately compare between unprocessed and decellularized hearts, and to account for mass and intracellular protein loss during the decellularization process, samples were normalized per whole heart. Lysate samples were submitted to the UF Mass Spectrometry Research and Education Center for processing and analysis. Samples were separated with sodium dodecyl sulfate-polyacrylamide gel electrophoresis (SDS-PAGE). Gels were digested with sequencing grade trypsin, trimmed into pieces, and exposed to a series of wash, dehydration, and rehydration steps (wash with water and 1:1 v/v methanol:50 mM ammonium bicarbonate, dehydrated with 1:1 v/v acetonitrile:50 mM ammonium bicarbonate, rehydrated in 25 mM dithiothreitol/100 mM ammonium bicarbonate solution and 55 mM Iodoacetamine/100 mM ammonium bicarbonate solution, washed with water, dehydrated with acetronitrile/ammonium bicarobonate). Gel pieces were then rehydrated in trypsin and Protease MAX surfactant (Promega), and the digestion was stopped with 0.5% TFA. MS analysis was performed immediately. Nano-liquid chromatography tandem mass spectrometry (Nano-LC/MS/MS) was performed on a Thermo Scientific Q Exactive HF Orbitrap mass spectrometer equipped with an EASY Spray nanospray source (Thermo Scientific) operated in positive ion mode.  The LC system was an UltiMate™ 3000 RSLCnano system from Thermo Scientific. The total run time was 260 minutes.  The MS/MS was acquired according to standard conditions established in the MS core facility.  The EASY Spray source operated with a spray voltage of 1.5 KV and a capillary temperature of 200°C.  The scan sequence of the mass spectrometer was based on the original TopTen™ method; the analysis was programmed for a full scan recorded between 375 – 1575 Da at 60,000 resolution, and a MS/MS scan at resolution 15,000 to generate product ion spectra to determine amino acid sequence in consecutive instrument scans of the fifteen most abundant peaks in the spectrum. All MS/MS samples were analyzed using Sequest (XCorr Only) (Thermo Fisher Scientific, San Jose, CA, USA; version IseNode in Proteome Discoverer 2.2.0.388). Sequest (XCorr Only) was set up to search Mus musculus assuming the digestion used the enzyme trypsin. Sequest (XCorr Only) was searched with a fragment ion mass tolerance of 0.020 Da and a precursor ion tolerance of 10.0 ppm. Carbamidomethyl of cysteine was specified in Sequest (XCorr Only) as a fixed modification. Deamidation of asparagine and oxidation of methionine were specified in Sequest (XCorr Only) as variable modifications. Scaffold (version Scaffold_4.9.0, Proteome Software Inc., Portland, OR) was used to validate MS/MS based peptide and protein identifications. Peptide identifications were accepted if they could be established at greater than 95.0% probability by the Peptide Prophet algorithm(1) with Scaffold delta-mass correction. Protein identifications were accepted if they could be established at greater than 99.0% probability and contained at least one identified peptide. Protein probabilities were assigned by the Protein Prophet algorithm(2). Proteins that contained similar peptides and could not be differentiated based on MS/MS analysis alone were grouped to satisfy the principles of parsimony. Proteins sharing significant peptide similarity were grouped into clusters.

**Mechanical characterization of neonatal mouse hearts.**

As the extracellular matrix largely contributes to the structural properties of tissue, which can in turn influence cellular behavior(3), it was of interest to measure mechanical properties of the heart tissue to better understand downstream effects of the *Nkx2-5* mutation and potential differential ECM deposition during development. Embryonic heart samples were too small to measure using indentation, hence this assessment was performed only on neonatal hearts. To perform mechanical testing, neonatal hearts were harvested and kept in Hank’s Balanced Salt Solution (HBSS) on ice for 2-4 hours to cease tissue contractions that cause run failure during mechanical testing. Hearts were placed on a microscope slide and thickness was measured with digital calipers. A Hysitron Biosoft *In Situ* Indenter (Bruker) equipped with an 800 µm-diameter spherical probe was used to indent into 5 percent of the thickness of the heart. An analysis method described in Stewart et al.(4) was utilized. Briefly, MATLAB was used to convert force readings from the indenter into effective modulus values using the Hertz contact model equation(5,6). These modulus values relative to time were fit to the Standard Linear Solid model for viscoelastic materials(7) to obtain useful metrics such as steady state modulus (SSM) and characteristic time. The SSM of both mutant and wild type hearts was found to be in the range of 0.5-0.7 kPa, and the characteristic times of both tissues were about 35-40 seconds (**Supplementary Figure 1).** Neither of the mechanical properties differed significantly between wild type and mutant tissues, demonstrating that although gross morphological differences and compositional ECM differences exist, the bulk mechanical properties are not affected.

A limitation to the methods employed is the sensitivity of the mechanical characterization device used. For example, embryonic hearts could not to be indented in this study because they were too small to satisfy common sample requirements of indentation(4,8). Additionally, the smaller the probe size used, the less sensitive the force readings are, which can pose an issue when dealing with soft materials such as these (<1 kPa steady state modulus)(8). It is possible that although the bulk properties remain unchanged, there are local mechanical differences between the two groups. Perhaps these could be measured with other methods, such as atomic force microscopy or other nano-scale indentation techniques.

**References**

1. Keller A, Nesvizhskii AI, Kolker E, Aebersold R. Empirical statistical model to estimate the accuracy of peptide identifications made by MS/MS and database search. *Anal Chem* (2002) doi:10.1021/ac025747h

2. Nesvizhskii AI, Keller A, Kolker E, Aebersold R. A statistical model for identifying proteins by tandem mass spectrometry. *Anal Chem* (2003) doi:10.1021/ac0341261

3. Kular JK, Basu S, Sharma RI. The extracellular matrix: Structure, composition, age-related differences, tools for analysis and applications for tissue engineering. *J Tissue Eng* (2014) doi:10.1177/2041731414557112

4. Stewart DC, Rubiano A, Dyson K, Simmons CS. Mechanical characterization of human brain tumors from patients and comparison to potential surgical phantoms. *PLoS One* (2017) doi:10.1371/journal.pone.0177561

5. Johnson KL. Contact Mechanics. (1989) doi:10.1201/b17110-2

6. G GFF. Hertz’s miscellaneous papers. *Nature* (1896) doi:10.1038/055006f0

7. Cowin SC, Doty SB. *Tissue mechanics*. (2007). doi:10.1007/978-0-387-49985-7

8. Rubiano A, Galitz C, Simmons CS. Mechanical Characterization by Mesoscale Indentation: Advantages and Pitfalls for Tissue and Scaffolds. *Tissue Eng - Part C Methods* (2019) doi:10.1089/ten.tec.2018.0372

**2.1. Supplementary Figures**

**Supplementary Figure 1: Bulk mechanical properties unaffected despite morphological differences.** A) Steady state modulus and C) characteristic time mechanical assessment of neonatal hearts (wild n=9, mutant n=6). Data presented as mean ± S.D. No significant differences were detected in mechanical properties with statistical significance of p<0.05.


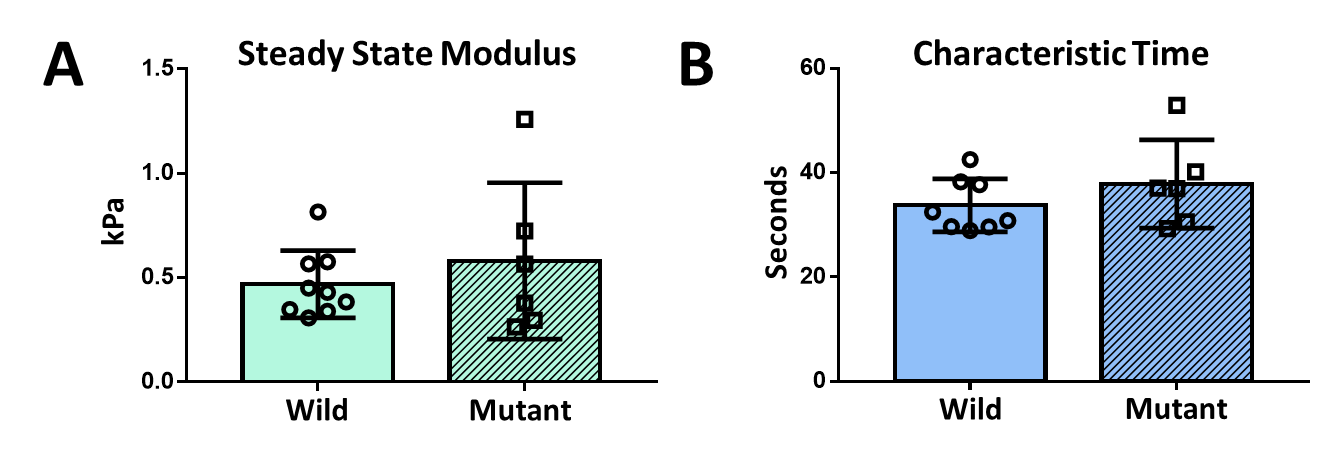


**Supplementary Figure 2: Laminin and collagen IV immunostaining and quantification reveals some organizational differences between wild type and *Nkx2-5* mutant ECM** A) Collagen IV (red) immunostaining on both embryonic and neonatal wild type and *Nkx2-5* mouse hearts. Embryonic scale bars: 100 µm. Neonatal scale bars: 200 µm. B) Example images of semi-quantitative analysis of laminin immunostaining (yellow). Uniformly sized, evenly spaced out regions of interest were selected along the ventricular walls of the heart sections. In each region of interest, pores were traced and assessed for area, circularity, ellipse fitting, etc. Researchers were blinded to ROI identities during quantification. Laminin staining used Alexa Fluor 647 dye but was pseudo-colored yellow for ease of comparison across graphics. C) Quantification of neonatal pore alignment, assessed by fitting ellipses to pores, measuring their angle relative to the horizontal axis, and comparing the standard deviations of angles from each sample. n=11 total. Graph displays mean ± standard deviation. No significant differences detected with p<0.05.

**
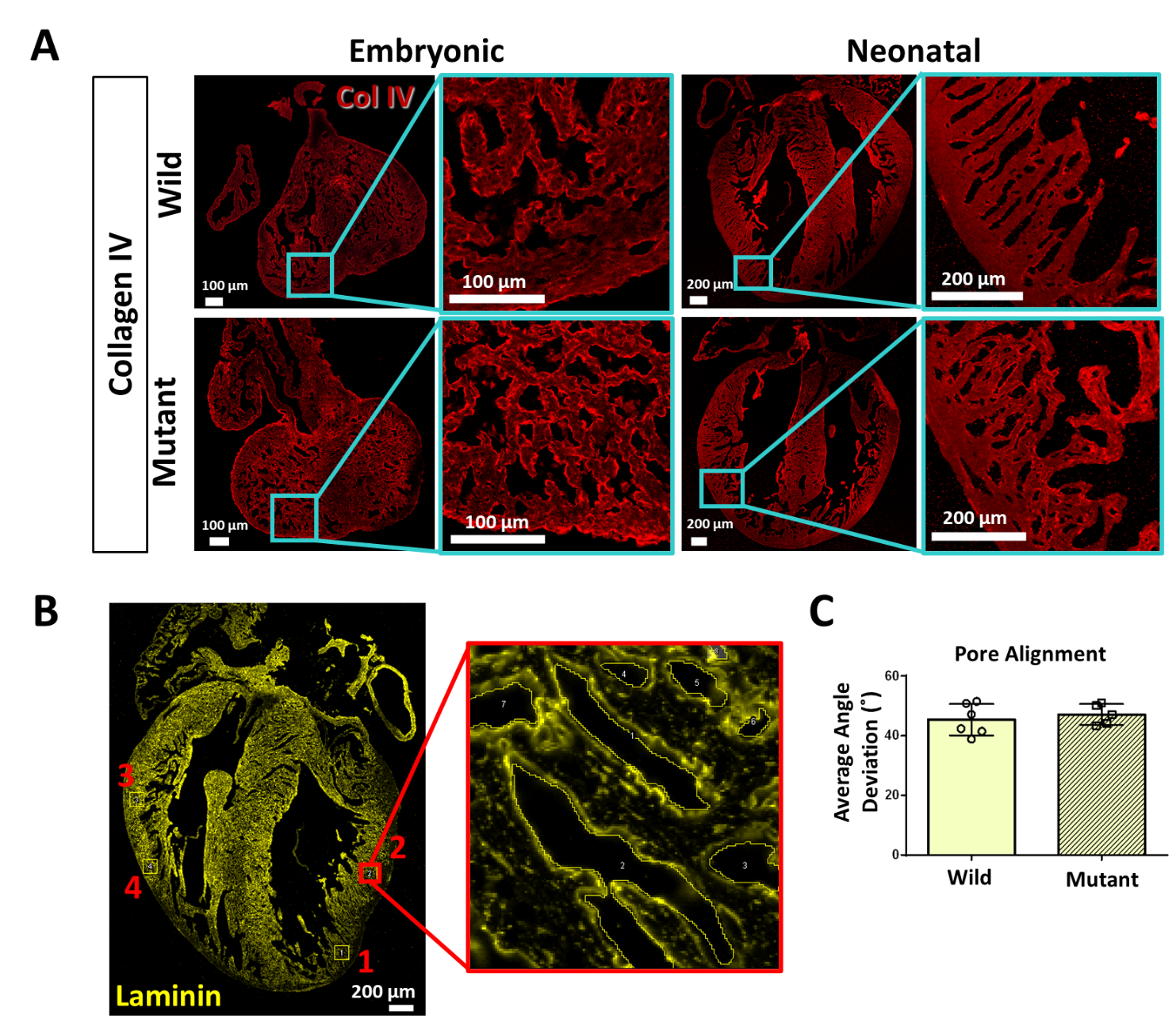
**

**Supplementary Figure 3: Western blotting demonstrates no significant differences in integrin α-5 expression between wild type and mutant neonatal *Nkx2-5* hearts.** Capillary-based Western botting images and corresponding quantifications of neonatal wild type and mutant integrin α-5. Graph displays mean ± standard deviation of protein band intensities normalized to lamin B1 housekeeping protein. n=8 per group. No statistically significant differences were detected between groups with p<0.05.

**
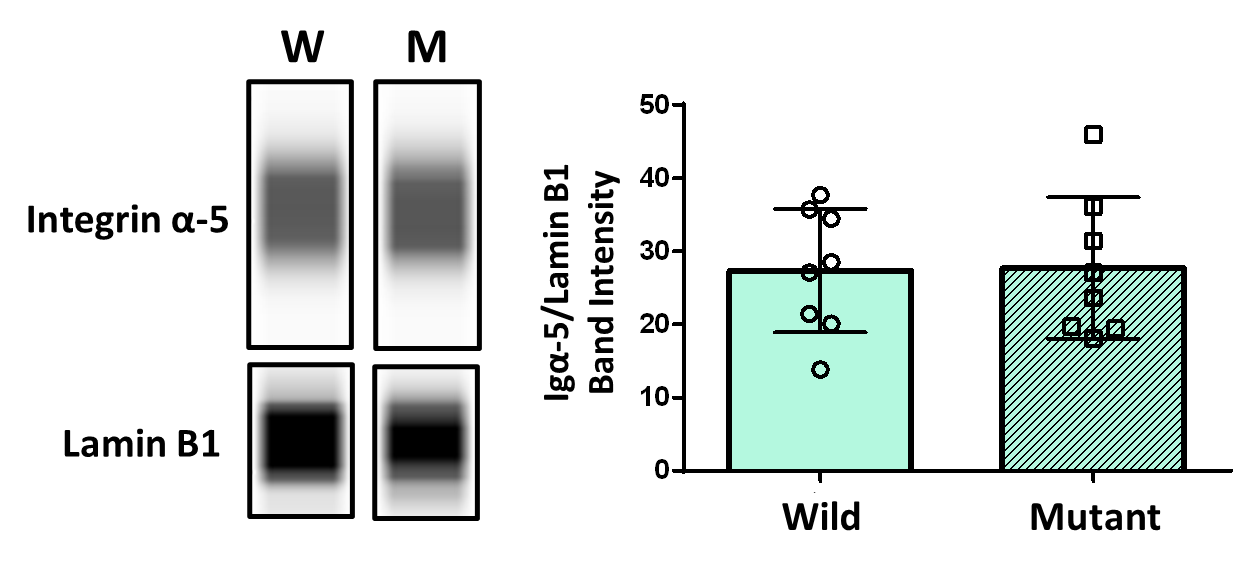
**


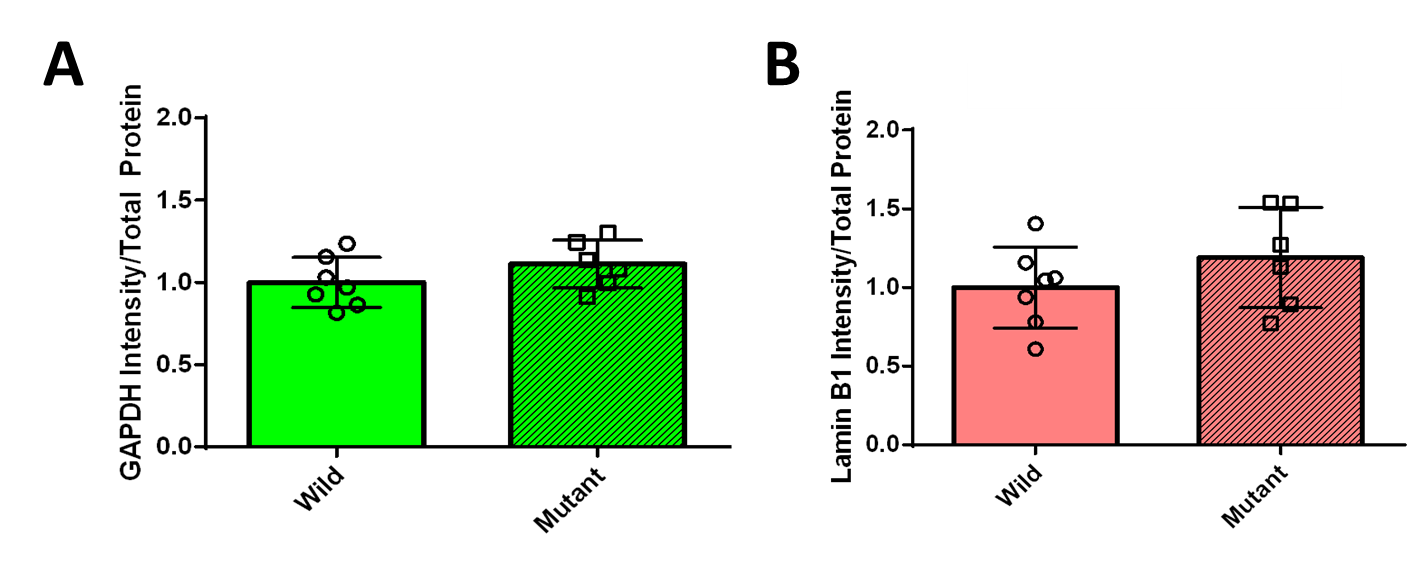
**Supplementary Figure 4: Validation of housekeeping proteins GAPDH and Lamin B1 for Western blot normalization.** Western band intensities of A) GAPDH and B) Lamin B1 normalized to total protein. Data presented as mean ± standard deviation. No significant differences were detected with statistical significance of p<0.05, hence normalizing other target protein band intensities to those of GAPDH or Lamin B1 should not statistically skew any results.

**Supplementary Table 1:** Proteins identified in mass spectrometry on decellularized wild type and *Nkx2-5* mutant mouse hearts (n=3 for each group). Only proteins with at least one average weighted spectra were included.

| Identified Proteins With At Least 1 Average Spectral Count | **Fisher's Exact Test (p-value): *(p < 0.00247)** | **Fold Change by Category (Mutant/Wild)** | **Wild Type 1 Weighted Spectra** | **Wild Type 2 Weighted Spectra** | **Wild Type 3 Weighted Spectra** | **Mutant 1 Weighted Spectra** | **Mutant 2 Weighted Spectra** | **Mutant 3 Weighted Spectra** | **Average Wild Type Spectra** | **Average Mutant Spectra** | **Wild Type S.E.M.** | **Mutant S.E.M.** |
| --- | --- | --- | --- | --- | --- | --- | --- | --- | --- | --- | --- | --- |
| sp\|Q02566\|MYH6_MOUSE Myosin-6 OS=Mus musculus GN=Myh6 PE=1 SV=2 [17] | < 0.00010 | 1.1 | 820 | 549 | 532 | 872 | 532 | 618 | 633.71 | 673.93 | 93.06 | 102.15 |
| Cluster of sp\|A2ASS6\|TITIN_MOUSETitin OS=Mus musculus GN=Ttn PE=1 SV=1 [2] | < 0.00010 | 1 | 432 | 235 | 142 | 367 | 186 | 232 | 269.52 | 261.67 | 85.29 | 54.49 |
| sp\|Q91Z83\|MYH7_MOUSE Myosin-7 OS=Mus musculus GN=Myh7 PE=2 SV=1 | < 0.00010 | 1 | 255 | 191 | 181 | 265 | 179 | 192 | 209.06 | 211.93 | 23.01 | 26.57 |
| Cluster of sp\|P00761\|TRYP_PIG Trypsin OS=Sus scrofa PE=1 SV=1 | < 0.00010 | 1.1 | 137 | 133 | 139 | 141 | 155 | 134 | 136.33 | 143.33 | 1.76 | 6.17 |
| sp\|P00761\|TRYP_PIG Trypsin OS=Sus scrofa PE=1 SV=1 | < 0.00010 | 0.9 | 175 | 186 | 187 | 187 | 201 | 194 | 182.67 | 194.00 | 3.84 | 4.04 |
| Cluster of sp\|Q02566\|MYH6_MOUSE Myosin-6 OS=Mus musculus GN=Myh6 PE=1 SV=2 | < 0.00010 | 0.9 | 118 | 64 | 51 | 124 | 59 | 74 | 77.65 | 85.78 | 20.36 | 19.80 |
| sp\|Q9JHU4\|DYHC1_MOUSE Cytoplasmic dynein 1 heavy chain 1 OS=Mus musculus GN=Dync1h1 PE=1 SV=2 | < 0.00010 | 0.2 | 52 | 13 | 0 | 6 | 4 | 0 | 21.67 | 3.33 | 15.62 | 1.76 |
| Cluster of sp\|O70133\|DHX9_MOUSE ATP-dependent RNA helicase A OS=Mus musculus GN=Dhx9 PE=1 SV=2 | < 0.00010 | 0.1 | 25 | 8 | 2 | 0 | 2 | 3 | 11.61 | 1.74 | 7.11 | 1.00 |
| sp\|P26039\|TLN1_MOUSE Talin-1 OS=Mus musculus GN=Tln1 PE=1 SV=2 | < 0.00010 | 0.1 | 25 | 3 | 1 | 2 | 1 | 0 | 9.67 | 1.00 | 7.69 | 0.58 |
| sp\|Q78PY7\|SND1_MOUSE Staphylococcal nuclease domain-containing protein 1 OS=Mus musculus GN=Snd1 PE=1 SV=1 | < 0.00010 | 0.1 | 23 | 6 | 0 | 1 | 0 | 2 | 9.67 | 1.00 | 6.89 | 0.58 |
| sp\|Q60597\|ODO1_MOUSE 2-oxoglutarate dehydrogenase, mitochondrial OS=Mus musculus GN=Ogdh PE=1 SV=3 | < 0.00010 | 0 | 21 | 1 | 0 | 0 | 0 | 0 | 7.33 | 0.00 | 6.84 | 0.00 |
| sp\|P08779\|K1C16_HUMAN Keratin, type I cytoskeletal 16 OS=Homo sapiens GN=KRT16 PE=1 SV=4 | < 0.00010 | 0 | 8 | 9 | 0 | 0 | 0 | 0 | 5.53 | 0.00 | 2.79 | 0.00 |
| sp\|Q04695\|K1C17_HUMAN Keratin, type I cytoskeletal 17 OS=Homo sapiens GN=KRT17 PE=1 SV=2 | < 0.00010 | 0 | 1 | 17 | 0 | 0 | 0 | 0 | 6.04 | 0.00 | 5.37 | 0.00 |
| Cluster of sp\|E9Q557\|DESP_MOUSE Desmoplakin OS=Mus musculus GN=Dsp PE=1 SV=1 | 0.00011 | 0 | 15 | 1 | 0 | 0 | 0 | 0 | 5.45 | 0.00 | 4.79 | 0.00 |
| sp\|Q02053\|UBA1_MOUSE Ubiquitin-like modifier-activating enzyme 1 OS=Mus musculus GN=Uba1 PE=1 SV=1 | 0.00012 | 0.09 | 23 | 0 | 0 | 1 | 1 | 0 | 7.67 | 0.67 | 7.67 | 0.33 |
| sp\|P68040\|RACK1_MOUSE Receptor of activated protein C kinase 1 OS=Mus musculus GN=Rack1 PE=1 SV=3 | 0.00033 | 0 | 11 | 3 | 0 | 0 | 0 | 0 | 4.67 | 0.00 | 3.28 | 0.00 |
| sp\|P36578\|RL4_HUMAN 60S ribosomal protein L4 OS=Homo sapiens GN=RPL4 PE=1 SV=5 | 0.00034 | INF | 11 | 4 | 0 | 0 | 0 | 0 | 5.00 | 0.00 | 3.21 | 0.00 |
| sp\|P09411\|PGK1_MOUSE Phosphoglycerate kinase 1 OS=Mus musculus GN=Pgk1 PE=1 SV=4 | 0.00038 | 6.2 | 25 | 3 | 3 | 5 | 0 | 0 | 10.33 | 1.67 | 7.33 | 1.67 |
| sp\|P11276\|FINC_MOUSE Fibronectin OS=Mus musculus GN=Fn1 PE=1 SV=4 | 0.00044 | 1.1 | 71 | 36 | 48 | 75 | 50 | 48 | 51.67 | 57.67 | 10.27 | 8.69 |
| sp\|P13645\|K1C10_HUMAN Keratin, type I cytoskeletal 10 OS=Homo sapiens GN=KRT10 PE=1 SV=6 | 0.00048 | 1.5 | 15 | 17 | 10 | 13 | 25 | 25 | 14.14 | 21.00 | 2.12 | 4.00 |
| sp\|P08660\|AK3_ECOLI Lysine-sensitive aspartokinase 3 OS=Escherichia coli (strain K12) GN=lysC PE=1 SV=2 | 0.00056 | INF | 0 | 0 | 0 | 7 | 2 | 0 | 0.00 | 3.00 | 0.00 | 2.08 |
| Cluster of sp\|O35763\|MOES_RAT Moesin OS=Rattus norvegicus GN=Msn PE=1 SV=3 | 0.00059 | 0 | 12 | 1 | 0 | 0 | 0 | 0 | 4.37 | 0.00 | 3.99 | 0.00 |
| sp\|Q61879\|MYH10_MOUSEMyosin-10 OS=Mus musculus GN=Myh10 PE=1 SV=2 | 0.00081 | 1 | 122 | 72 | 73 | 129 | 77 | 65 | 88.93 | 90.10 | 16.43 | 19.80 |
| sp\|Q9WUB3\|PYGM_MOUSE Glycogen phosphorylase, muscle form OS=Mus musculus GN=Pygm PE=1 SV=3 | 0.00085 | 0.3 | 34 | 3 | 2 | 5 | 2 | 3 | 12.67 | 3.33 | 10.43 | 0.88 |
| sp\|Q62188\|DPYL3_MOUSE Dihydropyrimidinase-related protein 3 OS=Mus musculus GN=Dpysl3 PE=1 SV=1 | 0.0012 | 0.3 | 30 | 3 | 2 | 7 | 2 | 0 | 11.55 | 3.00 | 9.06 | 2.08 |
| sp\|P00562\|AK2H_ECOLI Bifunctional aspartokinase/homoserine dehydrogenase 2 OS=Escherichia coli (strain K12) GN=metL PE=1 SV=3 | 0.0013 | INF | 0 | 0 | 0 | 5 | 2 | 1 | 0.00 | 2.67 | 0.00 | 1.20 |
| Cluster of sp\|P31001\|DESM_MOUSE Desmin OS=Mus musculus GN=Des PE=1 SV=3 | 0.0017 | 0.7 | 17 | 9 | 6 | 20 | 11 | 15 | 10.61 | 14.92 | 3.28 | 2.60 |
| sp\|Q6PB66\|LPPRC_MOUSE Leucine-rich PPR motif-containing protein, mitochondrial OS=Mus musculus GN=Lrpprc PE=1 SV=2 | 0.0019 | 0 | 11 | 0 | 0 | 0 | 0 | 0 | 3.67 | 0.00 | 3.67 | 0.00 |
| sp\|P54071\|IDHP_MOUSE Isocitrate dehydrogenase [NADP], mitochondrial OS=Mus musculus GN=Idh2 PE=1 SV=3 | 0.0022 | 0.1 | 14 | 3 | 0 | 0 | 0 | 2 | 5.67 | 0.67 | 4.26 | 0.67 |
| sp\|Q8CI94\|PYGB_MOUSE Glycogen phosphorylase, brain form OS=Mus musculus GN=Pygb PE=1 SV=3 | 0.0024 | 0.3 | 32 | 6 | 2 | 8 | 3 | 2 | 13.83 | 4.33 | 9.40 | 1.85 |
| sp\|P12883\|MYH7_HUMAN Myosin-7 OS=Homo sapiens GN=MYH7 PE=1 SV=5 | 0.0027 | 1.1 | 54 | 37 | 30 | 57 | 39 | 37 | 40.27 | 44.27 | 7.05 | 6.57 |
| sp\|O35501\|GRP75_CRIGR Stress-70 protein, mitochondrial OS=Cricetulus griseus GN=HSPA9 PE=2 SV=1 | 0.0035 | 0.2 | 17 | 3 | 1 | 3 | 1 | 0 | 6.89 | 1.33 | 4.96 | 0.88 |
| sp\|P21981\|TGM2_MOUSE Protein-glutamine gamma-glutamyltransferase 2 OS=Mus musculus GN=Tgm2 PE=1 SV=4 | 0.004 | 0.5 | 7 | 1 | 1 | 7 | 7 | 5 | 3.00 | 6.33 | 2.00 | 0.67 |
| Cluster of sp\|P10126\|EF1A1_MOUSE Elongation factor 1-alpha 1 OS=Mus musculus GN=Eef1a1 PE=1 SV=3 | 0.0048 | 0.4 | 50 | 8 | 3 | 14 | 7 | 6 | 20.62 | 8.70 | 14.82 | 2.58 |
| sp\|P05090\|APOD_HUMAN Apolipoprotein D OS=Homo sapiens GN=APOD PE=1 SV=1 | 0.0049 | 0 | 0 | 0 | 0 | 0 | 0 | 6 | 0.00 | 2.00 | 0.00 | 2.00 |
| Cluster of sp\|Q8VDN2\|AT1A1_MOUSE Sodium/potassium-transporting ATPase subunit alpha-1 OS=Mus musculus GN=Atp1a1 PE=1 SV=1 | 0.0052 | 0.4 | 31 | 9 | 4 | 8 | 4 | 4 | 14.63 | 5.31 | 8.26 | 1.31 |
| Cluster of sp\|P17844\|DDX5_HUMAN Probable ATP-dependent RNA helicase DDX5 OS=Homo sapiens GN=DDX5 PE=1 SV=1 | 0.0057 | 0.4 | 25 | 10 | 4 | 4 | 5 | 5 | 13.30 | 4.79 | 6.07 | 0.17 |
| sp\|P12382\|PFKAL_MOUSE ATP-dependent 6-phosphofructokinase, liver type OS=Mus musculus GN=Pfkl PE=1 SV=4 | 0.0057 | 0.1 | 15 | 0 | 0 | 2 | 0 | 0 | 4.97 | 0.67 | 4.97 | 0.67 |
| sp\|P08003\|PDIA4_MOUSE Protein disulfide-isomerase A4 OS=Mus musculus GN=Pdia4 PE=1 SV=3 | 0.0058 | 0 | 6 | 1 | 2 | 0 | 0 | 0 | 3.00 | 0.00 | 1.53 | 0.00 |
| sp\|Q8VDJ3\|VIGLN_MOUSE Vigilin OS=Mus musculus GN=Hdlbp PE=1 SV=1 | 0.0058 | 0 | 9 | 0 | 0 | 0 | 0 | 0 | 3.00 | 0.00 | 3.00 | 0.00 |
| sp\|Q08945\|SSRP1_HUMAN FACT complex subunit SSRP1 OS=Homo sapiens GN=SSRP1 PE=1 SV=1 | 0.0058 | 0 | 7 | 2 | 0 | 0 | 0 | 0 | 3.00 | 0.00 | 2.08 | 0.00 |
| Cluster of sp\|P16858\|G3P_MOUSE Glyceraldehyde-3-phosphate dehydrogenase OS=Mus musculus GN=Gapdh PE=1 SV=2 | 0.006 | 1.4 | 21 | 12 | 4 | 28 | 12 | 11 | 12.34 | 16.71 | 5.02 | 5.58 |
| sp\|Q64727\|VINC_MOUSE Vinculin OS=Mus musculus GN=Vcl PE=1 SV=4 | 0.0065 | 0.08 | 12 | 0 | 0 | 1 | 0 | 0 | 4.00 | 0.33 | 4.00 | 0.33 |
| sp\|B7L4F3\|DAPB_ECO55 4-hydroxy-tetrahydrodipicolinate reductase OS=Escherichia coli (strain 55989 / EAEC) GN=dapB PE=3 SV=1 | 0.0068 | INF | 0 | 0 | 0 | 3 | 3 | 0 | 0.00 | 2.00 | 0.00 | 1.00 |
| sp\|Q02SZ7\|LYSC_PSEAB Lysyl endopeptidase OS=Pseudomonas aeruginosa (strain UCBPP-PA14) GN=prpL PE=1 SV=1 | 0.0075 | 0.2 | 1 | 1 | 0 | 3 | 3 | 3 | 0.67 | 3.00 | 0.33 | 0.00 |
| sp\|Q9D8N0\|EF1G_MOUSE Elongation factor 1-gamma OS=Mus musculus GN=Eef1g PE=1 SV=3 | 0.0083 | INF | 8 | 1 | 0 | 0 | 0 | 0 | 3.00 | 0.00 | 2.52 | 0.00 |
| sp\|P02768\|ALBU_HUMAN Serum albumin OS=Homo sapiens GN=ALB PE=1 SV=2 | 0.0083 | INF | 0 | 1 | 8 | 0 | 0 | 0 | 3.00 | 0.00 | 2.52 | 0.00 |
| sp\|P57780\|ACTN4_MOUSE Alpha-actinin-4 OS=Mus musculus GN=Actn4 PE=1 SV=1 | 0.0086 | 0.2 | 18 | 1 | 3 | 0 | 2 | 3 | 7.19 | 1.60 | 5.23 | 0.90 |
| sp\|P09405\|NUCL_MOUSE Nucleolin OS=Mus musculus GN=Ncl PE=1 SV=2 | 0.009 | 0.3 | 19 | 6 | 4 | 5 | 2 | 2 | 9.67 | 3.00 | 4.70 | 1.00 |
| sp\|P36578\|RL4_HUMAN 60S ribosomal protein L4 OS=Homo sapiens GN=RPL4 PE=1 SV=5 | 0.009 | 0.1 | 12 | 2 | 0 | 1 | 1 | 0 | 4.67 | 0.67 | 3.71 | 0.33 |
| sp\|Q8CIE6\|COPA_MOUSE Coatomer subunit alpha OS=Mus musculus GN=Copa PE=1 SV=2 | 0.009 | 0.1 | 13 | 1 | 0 | 2 | 0 | 0 | 4.67 | 0.67 | 4.18 | 0.67 |
| sp\|Q8CGC7\|SYEP_MOUSE Bifunctional glutamate/proline--tRNA ligase OS=Mus musculus GN=Eprs PE=1 SV=4 | 0.009 | 0.1 | 13 | 1 | 0 | 1 | 1 | 0 | 4.67 | 0.67 | 4.18 | 0.33 |
| sp\|P51991\|ROA3_HUMAN Heterogeneous nuclear ribonucleoprotein A3 OS=Homo sapiens GN=HNRNPA3 PE=1 SV=2 | 0.0097 | 5 | 14 | 5 | 1 | 3 | 1 | 0 | 6.67 | 1.33 | 3.84 | 0.88 |
| Cluster of sp\|P70414\|NAC1_MOUSE Sodium/calcium exchanger 1 OS=Mus musculus GN=Slc8a1 PE=1 SV=1 | 0.01 | 0 | 4 | 4 | 0 | 0 | 0 | 0 | 2.67 | 0.00 | 1.33 | 0.00 |
| sp\|Q9WUA3\|PFKAP_MOUSE ATP-dependent 6-phosphofructokinase, platelet type OS=Mus musculus GN=Pfkp PE=1 SV=1 | 0.01 | 0 | 8 | 0 | 0 | 0 | 0 | 0 | 2.51 | 0.00 | 2.51 | 0.00 |
| Cluster of sp\|Q9CQN1\|TRAP1_MOUSE Heat shock protein 75 kDa, mitochondrial OS=Mus musculus GN=Trap1 PE=1 SV=1) | 0.01 | 0 | 8 | 0 | 0 | 0 | 0 | 0 | 2.81 | 0.00 | 2.81 | 0.00 |
| sp\|Q2PQA9\|KINH_RAT Kinesin-1 heavy chain OS=Rattus norvegicus GN=Kif5b PE=1 SV=1 | 0.01 | 0 | 8 | 0 | 0 | 0 | 0 | 0 | 2.67 | 0.00 | 2.67 | 0.00 |
| sp\|P97310\|MCM2_MOUSE DNA replication licensing factor MCM2 OS=Mus musculus GN=Mcm2 PE=1 SV=3 | 0.011 | 0.09 | 9 | 2 | 0 | 1 | 0 | 0 | 3.67 | 0.33 | 2.73 | 0.33 |
| Cluster of sp\|P04256\|ROA1_RAT Heterogeneous nuclear ribonucleoprotein A1 OS=Rattus norvegicus GN=Hnrnpa1 PE=1 SV=3 | 0.012 | 6 | 11 | 6 | 0 | 3 | 0 | 0 | 5.74 | 0.96 | 3.31 | 0.96 |
| sp\|P02463\|CO4A1_MOUSE Collagen alpha-1(IV) chain OS=Mus musculus GN=Col4a1 PE=1 SV=4 | 0.013 | 2.6 | 2 | 1 | 2 | 7 | 4 | 2 | 1.67 | 4.33 | 0.33 | 1.45 |
| sp\|P16546\|SPTN1_MOUSE Spectrin alpha chain, non-erythrocytic 1 OS=Mus musculus GN=Sptan1 PE=1 SV=4 | 0.013 | 0.4 | 28 | 12 | 11 | 6 | 9 | 7 | 17.00 | 7.33 | 5.51 | 0.88 |
| sp\|Q91VD9\|NDUS1_MOUSE NADH-ubiquinone oxidoreductase 75 kDa subunit, mitochondrial OS=Mus musculus GN=Ndufs1 PE=1 SV=2 | 0.013 | 0.3 | 14 | 7 | 1 | 4 | 1 | 1 | 7.33 | 2.00 | 3.76 | 1.00 |
| Cluster of sp\|P42208\|SEPT2_MOUSE Septin-2 OS=Mus musculus GN=Sept2 PE=1 SV=2 | 0.014 | INF | 8 | 0 | 0 | 0 | 0 | 0 | 2.67 | 0.00 | 2.67 | 0.00 |
| sp\|Q99KI0\|ACON_MOUSE Aconitate hydratase, mitochondrial OS=Mus musculus GN=Aco2 PE=1 SV=1 | 0.015 | 0.4 | 32 | 9 | 6 | 12 | 3 | 5 | 15.67 | 6.67 | 8.21 | 2.73 |
| sp\|Q9JLT0\|MYH10_RAT Myosin-10 OS=Rattus norvegicus GN=Myh10 PE=1 SV=1 | 0.015 | 0.2 | 2 | 0 | 0 | 6 | 2 | 0 | 0.67 | 2.67 | 0.67 | 1.76 |
| sp\|P02469\|LAMB1_MOUSE Laminin subunit beta-1 OS=Mus musculus GN=Lamb1 PE=1 SV=3 | 0.017 | 1.2 | 22 | 14 | 11 | 24 | 18 | 14 | 15.67 | 18.67 | 3.28 | 2.91 |
| sp\|Q68FD5\|CLH1_MOUSE Clathrin heavy chain 1 OS=Mus musculus GN=Cltc PE=1 SV=3 | 0.018 | 0.5 | 43 | 15 | 3 | 13 | 7 | 9 | 20.33 | 9.67 | 11.85 | 1.76 |
| sp\|Q8BTM8\|FLNA_MOUSE Filamin-A OS=Mus musculus GN=Flna PE=1 SV=5 | 0.018 | 0.3 | 21 | 0 | 0 | 5 | 1 | 0 | 6.84 | 2.09 | 6.84 | 1.61 |
| Cluster of sp\|P47942\|DPYL2_RAT Dihydropyrimidinase-related protein 2 OS=Rattus norvegicus GN=Dpysl2 PE=1 SV=1 | 0.018 | 0.3 | 21 | 0 | 0 | 6 | 0 | 0 | 7.11 | 2.00 | 7.11 | 2.00 |
| sp\|Q62167\|DDX3X_MOUSE ATP-dependent RNA helicase DDX3X OS=Mus musculus GN=Ddx3x PE=1 SV=3 | 0.018 | 0.2 | 12 | 4 | 1 | 1 | 2 | 1 | 5.67 | 1.33 | 3.28 | 0.33 |
| Cluster of sp\|Q64399\|TOP2B_CRILO DNA topoisomerase 2-beta OS=Cricetulus longicaudatus GN=TOP2B PE=2 SV=1 | 0.018 | 0.1 | 9 | 2 | 0 | 0 | 0 | 1 | 3.44 | 0.33 | 2.60 | 0.33 |
| sp\|P46935\|NEDD4_MOUSE E3 ubiquitin-protein ligase NEDD4 OS=Mus musculus GN=Nedd4 PE=1 SV=3 | 0.018 | 0 | 6 | 1 | 0 | 0 | 0 | 0 | 2.33 | 0.00 | 1.86 | 0.00 |
| sp\|P07355\|ANXA2_HUMAN Annexin A2 OS=Homo sapiens GN=ANXA2 PE=1 SV=2 | 0.018 | 0 | 0 | 7 | 0 | 0 | 0 | 0 | 2.33 | 0.00 | 2.33 | 0.00 |
| sp\|O35142\|COPB2_RAT Coatomer subunit beta' OS=Rattus norvegicus GN=Copb2 PE=1 SV=3 | 0.018 | 0 | 7 | 0 | 0 | 0 | 0 | 0 | 2.33 | 0.00 | 2.33 | 0.00 |
| sp\|P24527\|LKHA4_MOUSE Leukotriene A-4 hydrolase OS=Mus musculus GN=Lta4h PE=1 SV=4 | 0.018 | 0 | 7 | 0 | 0 | 0 | 0 | 0 | 2.33 | 0.00 | 2.33 | 0.00 |
| Cluster of sp\|P50719\|TBA_HAECO Tubulin alpha chain OS=Haemonchus contortus PE=2 SV=1 | 0.021 | 2.5 | 31 | 11 | 2 | 8 | 9 | 0 | 14.55 | 5.79 | 8.80 | 2.91 |
| sp\|P11499\|HS90B_MOUSEHeat shock protein HSP 90-beta OS=Mus musculus GN=Hsp90ab1 PE=1 SV=3 [5] | 0.022 | 0.6 | 148 | 41 | 24 | 67 | 32 | 32 | 70.90 | 43.73 | 38.91 | 11.76 |
| sp\|Q8VEK3\|HNRPU_MOUSE Heterogeneous nuclear ribonucleoprotein U OS=Mus musculus GN=Hnrnpu PE=1 SV=1 | 0.024 | 0.4 | 30 | 10 | 5 | 9 | 4 | 7 | 15.00 | 6.67 | 7.64 | 1.45 |
| Cluster of sp\|P20152\|VIME_MOUSE Vimentin OS=Mus musculus GN=Vim PE=1 SV=3 | 0.024 | 0.9 | 18 | 7 | 8 | 15 | 12 | 11 | 11.08 | 12.73 | 3.42 | 1.41 |
| sp\|Q62188\|DPYL3_MOUSE Dihydropyrimidinase-related protein 3 OS=Mus musculus GN=Dpysl3 PE=1 SV=1 | 0.024 | INF | 7 | 0 | 0 | 0 | 0 | 0 | 2.33 | 0.00 | 2.33 | 0.00 |
| sp\|P12970\|RL7A_MOUSE 60S ribosomal protein L7a OS=Mus musculus GN=Rpl7a PE=1 SV=2 | 0.024 | INF | 7 | 0 | 0 | 0 | 0 | 0 | 2.33 | 0.00 | 2.33 | 0.00 |
| sp\|Q61001\|LAMA5_MOUSE Laminin subunit alpha-5 OS=Mus musculus GN=Lama5 PE=1 SV=4 | 0.026 | 1.5 | 8 | 6 | 2 | 14 | 3 | 7 | 5.33 | 8.00 | 1.76 | 3.21 |
| sp\|Q05793\|PGBM_MOUSE Basement membrane-specific heparan sulfate proteoglycan core protein OS=Mus musculus GN=Hspg2 PE=1 SV=1 | 0.026 | 1.1 | 38 | 21 | 16 | 41 | 24 | 15 | 25.00 | 26.67 | 6.66 | 7.62 |
| sp\|P00974\|BPT1_BOVIN Pancreatic trypsin inhibitor OS=Bos taurus PE=1 SV=2 | 0.028 | 0.6 | 4 | 1 | 3 | 4 | 6 | 4 | 2.67 | 4.67 | 0.88 | 0.67 |
| sp\|P35059\|H4_ACRFO Histone H4 OS=Acropora formosa PE=3 SV=2 | 0.028 | 0.6 | 7 | 0 | 0 | 6 | 5 | 2 | 2.42 | 4.16 | 2.42 | 1.28 |
| sp\|P07901\|HS90A_MOUSEHeat shock protein HSP 90-alpha OS=Mus musculus GN=Hsp90aa1 PE=1 SV=4 | 0.029 | 0.5 | 45 | 11 | 5 | 16 | 9 | 6 | 20.69 | 10.31 | 12.45 | 3.07 |
| sp\|O88342\|WDR1_MOUSE WD repeat-containing protein 1 OS=Mus musculus GN=Wdr1 PE=1 SV=3 | 0.029 | 0.1 | 9 | 0 | 0 | 1 | 0 | 0 | 3.00 | 0.33 | 3.00 | 0.33 |
| sp\|Q61881\|MCM7_MOUSE DNA replication licensing factor MCM7 OS=Mus musculus GN=Mcm7 PE=1 SV=1 | 0.029 | 0.1 | 9 | 0 | 0 | 1 | 0 | 0 | 3.00 | 0.33 | 3.00 | 0.33 |
| sp\|P13020\|GELS_MOUSE Gelsolin OS=Mus musculus GN=Gsn PE=1 SV=3 | 0.029 | 0.1 | 9 | 0 | 0 | 1 | 0 | 0 | 3.00 | 0.33 | 3.00 | 0.33 |
| Cluster of sp\|Q9DC70\|NDUS7_MOUSE NADH dehydrogenase [ubiquinone] iron-sulfur protein 7, mitochondrial OS=Mus musculus GN=Ndufs7 PE=1 SV=1 | 0.029 | 0 | 0 | 0 | 0 | 2 | 2 | 0 | 0.00 | 1.19 | 0.00 | 0.59 |
| sp\|P35527\|K1C9_HUMAN Keratin, type I cytoskeletal 9 OS=Homo sapiens GN=KRT9 PE=1 SV=3 | 0.031 | 1.9 | 39 | 75 | 26 | 17 | 24 | 34 | 46.67 | 25.00 | 14.66 | 4.93 |
| sp\|Q6P2Q9\|PRP8_HUMAN Pre-mRNA-processing-splicing factor 8 OS=Homo sapiens GN=PRPF8 PE=1 SV=2 | 0.032 | 0 | 5 | 1 | 0 | 0 | 0 | 0 | 2.00 | 0.00 | 1.53 | 0.00 |
| sp\|Q6P5F9\|XPO1_MOUSE Exportin-1 OS=Mus musculus GN=Xpo1 PE=1 SV=1 | 0.032 | 0 | 5 | 1 | 0 | 0 | 0 | 0 | 2.00 | 0.00 | 1.53 | 0.00 |
| sp\|P97311\|MCM6_MOUSE DNA replication licensing factor MCM6 OS=Mus musculus GN=Mcm6 PE=1 SV=1 | 0.032 | 0 | 6 | 0 | 0 | 0 | 0 | 0 | 2.00 | 0.00 | 2.00 | 0.00 |
| sp\|Q0IIK5\|DDX1_BOVIN ATP-dependent RNA helicase DDX1 OS=Bos taurus GN=DDX1 PE=2 SV=1 | 0.032 | 0 | 5 | 1 | 0 | 0 | 0 | 0 | 2.00 | 0.00 | 1.53 | 0.00 |
| sp\|Q8BIJ6\|SYIM_MOUSE Isoleucine--tRNA ligase, mitochondrial OS=Mus musculus GN=Iars2 PE=1 SV=1 | 0.032 | 0 | 6 | 0 | 0 | 0 | 0 | 0 | 2.00 | 0.00 | 2.00 | 0.00 |
| Cluster of sp\|P11940\|PABP1_HUMAN Polyadenylate-binding protein 1 OS=Homo sapiens GN=PABPC1 PE=1 SV=2 | 0.032 | 0 | 6 | 0 | 0 | 0 | 0 | 0 | 2.00 | 0.00 | 2.00 | 0.00 |
| sp\|O54734\|OST48_MOUSE Dolichyl-diphosphooligosaccharide--protein glycosyltransferase 48 kDa subunit OS=Mus musculus GN=Ddost PE=1 SV=2 | 0.032 | 0 | 4 | 2 | 0 | 0 | 0 | 0 | 2.00 | 0.00 | 1.15 | 0.00 |
| sp\|Q922D8\|C1TC_MOUSE C-1-tetrahydrofolate synthase, cytoplasmic OS=Mus musculus GN=Mthfd1 PE=1 SV=4 | 0.032 | 0 | 6 | 0 | 0 | 0 | 0 | 0 | 2.00 | 0.00 | 2.00 | 0.00 |
| sp\|Q9Z2I8\|SUCB2_MOUSE Succinate--CoA ligase [GDP-forming] subunit beta, mitochondrial OS=Mus musculus GN=Suclg2 PE=1 SV=3 | 0.032 | 6 | 12 | 0 | 0 | 2 | 0 | 0 | 4.00 | 0.67 | 4.00 | 0.67 |
| Cluster of sp\|P17710\|HXK1_MOUSE Hexokinase-1 OS=Mus musculus GN=Hk1 PE=1 SV=3 | 0.033 | 0.3 | 16 | 4 | 3 | 2 | 3 | 3 | 7.63 | 2.65 | 4.30 | 0.50 |
| Cluster of sp\|P54071\|IDHP_MOUSE Isocitrate dehydrogenase [NADP], mitochondrial OS=Mus musculus GN=Idh2 PE=1 SV=3 | 0.033 | 2.3 | 38 | 5 | 8 | 10 | 4 | 9 | 16.92 | 7.42 | 10.71 | 1.81 |
| sp\|P46462\|TERA_RAT Transitional endoplasmic reticulum ATPase OS=Rattus norvegicus GN=Vcp PE=1 SV=3 | 0.036 | 0.3 | 15 | 2 | 2 | 2 | 2 | 2 | 6.33 | 2.00 | 4.33 | 0.00 |
| Cluster of sp\|A5A6N4\|IF4A1_PANTR Eukaryotic initiation factor 4A-I OS=Pan troglodytes GN=EIF4A1 PE=2 SV=1 | 0.036 | 0.3 | 9 | 2 | 2 | 3 | 0 | 1 | 4.12 | 1.14 | 2.42 | 0.81 |
| sp\|P97807\|FUMH_MOUSE Fumarate hydratase, mitochondrial OS=Mus musculus GN=Fh PE=1 SV=3 | 0.036 | 0.2 | 13 | 0 | 0 | 1 | 1 | 1 | 4.33 | 1.00 | 4.33 | 0.00 |
| sp\|P21796\|VDAC1_HUMAN Voltage-dependent anion-selective channel protein 1 OS=Homo sapiens GN=VDAC1 PE=1 SV=2 | 0.036 | INF | 0 | 0 | 0 | 4 | 0 | 0 | 0.00 | 1.33 | 0.00 | 1.33 |
| Cluster of sp\|P16858\|G3P_MOUSE Glyceraldehyde-3-phosphate dehydrogenase OS=Mus musculus GN=Gapdh PE=1 SV=2 | 0.036 | 1.1 | 57 | 21 | 12 | 53 | 17 | 14 | 29.66 | 27.76 | 13.73 | 12.42 |
| sp\|P14824\|ANXA6_MOUSE Annexin A6 OS=Mus musculus GN=Anxa6 PE=1 SV=3 | 0.037 | 0.3 | 16 | 1 | 0 | 3 | 2 | 0 | 5.67 | 1.67 | 5.17 | 0.88 |
| sp\|Q04447\|KCRB_MOUSE Creatine kinase B-type OS=Mus musculus GN=Ckb PE=1 SV=1 | 0.037 | 4.7 | 12 | 2 | 0 | 1 | 1 | 1 | 4.67 | 1.00 | 3.71 | 0.00 |
| sp\|Q9DC69\|NDUA9_MOUSE NADH dehydrogenase [ubiquinone] 1 alpha subcomplex subunit 9, mitochondrial OS=Mus musculus GN=Ndufa9 PE=1 SV=2 | 0.039 | 9 | 6 | 2 | 1 | 1 | 0 | 0 | 3.00 | 0.33 | 1.53 | 0.33 |
| sp\|P97807\|FUMH_MOUSE Fumarate hydratase, mitochondrial OS=Mus musculus GN=Fh PE=1 SV=3 | 0.039 | 9 | 9 | 0 | 0 | 1 | 0 | 0 | 3.00 | 0.33 | 3.00 | 0.33 |
| Cluster of sp\|P52480\|KPYM_MOUSE Pyruvate kinase PKM OS=Mus musculus GN=Pkm PE=1 SV=4 | 0.041 | 0.6 | 69 | 23 | 12 | 27 | 17 | 15 | 34.76 | 19.98 | 17.21 | 3.77 |
| sp\|P05197\|EF2_RAT Elongation factor 2 OS=Rattus norvegicus GN=Eef2 PE=1 SV=4 | 0.041 | 0.5 | 50 | 15 | 9 | 16 | 12 | 12 | 24.67 | 13.33 | 12.78 | 1.33 |
| sp\|P05197\|EF2_RAT Elongation factor 2 OS=Rattus norvegicus GN=Eef2 PE=1 SV=4 | 0.041 | INF | 5 | 1 | 0 | 0 | 0 | 0 | 2.00 | 0.00 | 1.53 | 0.00 |
| sp\|P52272\|HNRPM_HUMAN Heterogeneous nuclear ribonucleoprotein M OS=Homo sapiens GN=HNRNPM PE=1 SV=3 | 0.041 | INF | 6 | 0 | 0 | 0 | 0 | 0 | 2.00 | 0.00 | 2.00 | 0.00 |
| sp\|P86203\|NDUS1_MESAU NADH-ubiquinone oxidoreductase 75 kDa subunit, mitochondrial (Fragments) OS=Mesocricetus auratus GN=NDUFS1 PE=1 SV=1 | 0.041 | INF | 6 | 0 | 0 | 0 | 0 | 0 | 2.00 | 0.00 | 2.00 | 0.00 |
| sp\|Q63065\|PDK1_RAT [Pyruvate dehydrogenase (acetyl-transferring)] kinase isozyme 1, mitochondrial OS=Rattus norvegicus GN=Pdk1 PE=1 SV=1 | 0.041 | INF | 5 | 1 | 0 | 0 | 0 | 0 | 2.00 | 0.00 | 1.53 | 0.00 |
| Cluster of sp\|P02104\|HBE_MOUSE Hemoglobin subunit epsilon-Y2 OS=Mus musculus GN=Hbb-y PE=1 SV=2 | 0.042 | 0.7 | 13 | 2 | 0 | 8 | 8 | 4 | 4.97 | 6.68 | 4.11 | 1.56 |
| sp\|P50752\|TNNT2_MOUSE Troponin T, cardiac muscle OS=Mus musculus GN=Tnnt2 PE=1 SV=2 | 0.042 | 0.5 | 2 | 2 | 1 | 2 | 5 | 3 | 1.67 | 3.33 | 0.33 | 0.88 |
| Cluster of sp\|P41340\|ACT3_LIMPO Actin-3 OS=Limulus polyphemus PE=1 SV=1 | 0.043 | 1.7 | 175 | 36 | 23 | 56 | 39 | 41 | 77.78 | 45.19 | 48.54 | 5.33 |
| Cluster of sp\|P05064\|ALDOA_MOUSE Fructose-bisphosphate aldolase A OS=Mus musculus GN=Aldoa PE=1 SV=2 | 0.043 | 3.3 | 22 | 0 | 0 | 7 | 0 | 0 | 7.43 | 2.25 | 7.43 | 2.25 |
| Cluster of sp\|P14733\|LMNB1_MOUSE Lamin-B1 OS=Mus musculus GN=Lmnb1 PE=1 SV=3 (sp\|P14733\|LMNB1_MOUSELamin-B1 OS=Mus musculus GN=Lmnb1 PE=1 SV=3) | 0.046 | 0.1 | 6 | 2 | 0 | 1 | 0 | 0 | 2.57 | 0.33 | 1.73 | 0.33 |
| sp\|P13645\|K1C10_HUMAN Keratin, type I cytoskeletal 10 OS=Homo sapiens GN=KRT10 PE=1 SV=6 | 0.046 | 1.1 | 31 | 48 | 23 | 30 | 26 | 36 | 34.00 | 30.67 | 7.37 | 2.91 |
| Cluster of sp\|Q8VDD5\|MYH9_MOUSE Myosin-9 OS=Mus musculus GN=Myh9 PE=1 SV=4 | 0.047 | 1 | 57 | 33 | 23 | 57 | 26 | 25 | 37.47 | 36.16 | 9.92 | 10.30 |
| sp\|Q7TPR4\|ACTN1_MOUSE Alpha-actinin-1 OS=Mus musculus GN=Actn1 PE=1 SV=1 | 0.049 | 0.3 | 14 | 3 | 2 | 2 | 0 | 3 | 6.28 | 1.85 | 3.75 | 0.95 |
| sp\|P02772\|FETA_MOUSE Alpha-fetoprotein OS=Mus musculus GN=Afp PE=2 SV=1 | 0.055 | 0.4 | 18 | 3 | 2 | 4 | 5 | 0 | 7.67 | 3.00 | 5.17 | 1.53 |
| sp\|Q61555\|FBN2_MOUSE Fibrillin-2 OS=Mus musculus GN=Fbn2 PE=1 SV=2 | 0.056 | 1.1 | 24 | 14 | 8 | 21 | 13 | 15 | 15.37 | 16.51 | 4.87 | 2.57 |
| sp\|Q02257\|PLAK_MOUSE Junction plakoglobin OS=Mus musculus GN=Jup PE=1 SV=3 | 0.057 | 0 | 4 | 2 | 0 | 0 | 0 | 0 | 1.71 | 0.00 | 1.03 | 0.00 |
| Cluster of sp\|P31001\|DESM_MOUSE Desmin OS=Mus musculus GN=Des PE=1 SV=3 | 0.057 | 1.1 | 18 | 8 | 8 | 18 | 9 | 11 | 11.16 | 12.60 | 3.21 | 2.73 |
| sp\|Q8BMF4\|ODP2_MOUSE Dihydrolipoyllysine-residue acetyltransferase component of pyruvate dehydrogenase complex, mitochondrial OS=Mus musculus GN=Dlat PE=1 SV=2 | 0.057 | 0 | 5 | 0 | 0 | 0 | 0 | 0 | 1.67 | 0.00 | 1.67 | 0.00 |
| sp\|Q8CAQ8\|MIC60_MOUSE MICOS complex subunit Mic60 OS=Mus musculus GN=Immt PE=1 SV=1 | 0.057 | 0 | 5 | 0 | 0 | 0 | 0 | 0 | 1.67 | 0.00 | 1.67 | 0.00 |
| sp\|Q9Z1E4\|GYS1_MOUSE Glycogen [starch] synthase, muscle OS=Mus musculus GN=Gys1 PE=1 SV=2 | 0.057 | 0 | 5 | 0 | 0 | 0 | 0 | 0 | 1.67 | 0.00 | 1.67 | 0.00 |
| sp\|P27659\|RL3_MOUSE 60S ribosomal protein L3 OS=Mus musculus GN=Rpl3 PE=1 SV=3 | 0.057 | 0 | 5 | 0 | 0 | 0 | 0 | 0 | 1.67 | 0.00 | 1.67 | 0.00 |
| Cluster of sp\|P13929\|ENOB_HUMAN Beta-enolase OS=Homo sapiens GN=ENO3 PE=1 SV=5 | 0.057 | 0 | 4 | 0 | 1 | 0 | 0 | 0 | 1.64 | 0.00 | 1.13 | 0.00 |
| Cluster of sp\|P49718\|MCM5_MOUSE DNA replication licensing factor MCM5 OS=Mus musculus GN=Mcm5 PE=1 SV=1 | 0.057 | 0 | 5 | 0 | 0 | 0 | 0 | 0 | 1.65 | 0.00 | 1.65 | 0.00 |
| sp\|Q3UPL0\|SC31A_MOUSE Protein transport protein Sec31A OS=Mus musculus GN=Sec31a PE=1 SV=2 | 0.057 | 0 | 5 | 0 | 0 | 0 | 0 | 0 | 1.67 | 0.00 | 1.67 | 0.00 |
| sp\|Q9D6Z1\|NOP56_MOUSE Nucleolar protein 56 OS=Mus musculus GN=Nop56 PE=1 SV=2 | 0.057 | 0 | 5 | 0 | 0 | 0 | 0 | 0 | 1.67 | 0.00 | 1.67 | 0.00 |
| sp\|Q8BH59\|CMC1_MOUSE Calcium-binding mitochondrial carrier protein Aralar1 OS=Mus musculus GN=Slc25a12 PE=1 SV=1 | 0.057 | 0 | 5 | 0 | 0 | 0 | 0 | 0 | 1.76 | 0.00 | 1.76 | 0.00 |
| sp\|A5A6M4\|EIF3L_PANTR Eukaryotic translation initiation factor 3 subunit L OS=Pan troglodytes GN=EIF3L PE=2 SV=1 | 0.057 | 0 | 5 | 0 | 0 | 0 | 0 | 0 | 1.67 | 0.00 | 1.67 | 0.00 |
| sp\|P50544\|ACADV_MOUSE Very long-chain specific acyl-CoA dehydrogenase, mitochondrial OS=Mus musculus GN=Acadvl PE=1 SV=3 | 0.057 | 0 | 5 | 0 | 0 | 0 | 0 | 0 | 1.67 | 0.00 | 1.67 | 0.00 |
| sp\|Q8BMK4\|CKAP4_MOUSE Cytoskeleton-associated protein 4 OS=Mus musculus GN=Ckap4 PE=1 SV=2 | 0.057 | 0 | 5 | 0 | 0 | 0 | 0 | 0 | 1.67 | 0.00 | 1.67 | 0.00 |
| sp\|P47934\|CACP_MOUSE Carnitine O-acetyltransferase OS=Mus musculus GN=Crat PE=1 SV=3 | 0.057 | 0 | 5 | 0 | 0 | 0 | 0 | 0 | 1.67 | 0.00 | 1.67 | 0.00 |
| sp\|Q91YQ5\|RPN1_MOUSE Dolichyl-diphosphooligosaccharide--protein glycosyltransferase subunit 1 OS=Mus musculus GN=Rpn1 PE=1 SV=1 | 0.057 | 0 | 5 | 0 | 0 | 0 | 0 | 0 | 1.67 | 0.00 | 1.67 | 0.00 |
| sp\|Q9CPN8\|IF2B3_MOUSE Insulin-like growth factor 2 mRNA-binding protein 3 OS=Mus musculus GN=Igf2bp3 PE=1 SV=1 | 0.057 | 0 | 5 | 0 | 0 | 0 | 0 | 0 | 1.82 | 0.00 | 1.82 | 0.00 |
| sp\|Q8BHN3\|GANAB_MOUSE Neutral alpha-glucosidase AB OS=Mus musculus GN=Ganab PE=1 SV=1 | 0.057 | 0 | 5 | 0 | 0 | 0 | 0 | 0 | 1.67 | 0.00 | 1.67 | 0.00 |
| sp\|Q9QXX4\|CMC2_MOUSE Calcium-binding mitochondrial carrier protein Aralar2 OS=Mus musculus GN=Slc25a13 PE=1 SV=1 | 0.057 | 0 | 5 | 0 | 0 | 0 | 0 | 0 | 1.57 | 0.00 | 1.57 | 0.00 |
| sp\|Q9DB77\|QCR2_MOUSE Cytochrome b-c1 complex subunit 2, mitochondrial OS=Mus musculus GN=Uqcrc2 PE=1 SV=1 | 0.057 | 3 | 17 | 3 | 1 | 4 | 2 | 1 | 7.00 | 2.33 | 5.03 | 0.88 |
| Cluster of sp\|P10126\|EF1A1_MOUSE Elongation factor 1-alpha 1 OS=Mus musculus GN=Eef1a1 PE=1 SV=3 | 0.058 | 2.1 | 44 | 10 | 5 | 11 | 11 | 7 | 19.88 | 9.64 | 12.38 | 1.39 |
| sp\|P19324\|SERPH_MOUSE Serpin H1 OS=Mus musculus GN=Serpinh1 PE=1 SV=3 | 0.068 | 0.5 | 28 | 10 | 10 | 12 | 4 | 9 | 16.00 | 8.33 | 6.00 | 2.33 |
| sp\|Q99KI0\|ACON_MOUSE Aconitate hydratase, mitochondrial OS=Mus musculus GN=Aco2 PE=1 SV=1 | 0.068 | 2.6 | 19 | 3 | 4 | 5 | 2 | 3 | 8.67 | 3.33 | 5.17 | 0.88 |
| sp\|A2C4U6\|EFG_PROM1 Elongation factor G OS=Prochlorococcus marinus (strain NATL1A) GN=fusA PE=3 SV=1 | 0.069 | 0.5 | 2 | 1 | 1 | 1 | 3 | 4 | 1.33 | 2.67 | 0.33 | 0.88 |
| sp\|P07310\|KCRM_MOUSE Creatine kinase M-type OS=Mus musculus GN=Ckm PE=1 SV=1 | 0.07 | INF | 5 | 0 | 0 | 0 | 0 | 0 | 1.67 | 0.00 | 1.67 | 0.00 |
| sp\|P29758\|OAT_MOUSE Ornithine aminotransferase, mitochondrial OS=Mus musculus GN=Oat PE=1 SV=1 | 0.07 | INF | 5 | 0 | 0 | 0 | 0 | 0 | 1.67 | 0.00 | 1.67 | 0.00 |
| sp\|Q68FX0\|IDH3B_RAT Isocitrate dehydrogenase [NAD] subunit beta, mitochondrial OS=Rattus norvegicus GN=Idh3B PE=2 SV=1 | 0.07 | INF | 5 | 0 | 0 | 0 | 0 | 0 | 1.67 | 0.00 | 1.67 | 0.00 |
| sp\|P14602\|HSPB1_MOUSE Heat shock protein beta-1 OS=Mus musculus GN=Hspb1 PE=1 SV=3 | 0.07 | 0 | 0 | 0 | 0 | 3 | 0 | 0 | 0.00 | 1.00 | 0.00 | 1.00 |
| sp\|Q9DCL9\|PUR6_MOUSE Multifunctional protein ADE2 OS=Mus musculus GN=Paics PE=1 SV=4 | 0.07 | INF | 5 | 0 | 0 | 0 | 0 | 0 | 1.67 | 0.00 | 1.67 | 0.00 |
| sp\|P54311\|GBB1_RAT Guanine nucleotide-binding protein G(I)/G(S)/G(T) subunit beta-1 OS=Rattus norvegicus GN=Gnb1 PE=1 SV=4 | 0.07 | INF | 5 | 0 | 0 | 0 | 0 | 0 | 1.53 | 0.00 | 1.53 | 0.00 |
| sp\|P25311\|ZA2G_HUMAN Zinc-alpha-2-glycoprotein OS=Homo sapiens GN=AZGP1 PE=1 SV=2 | 0.07 | 0 | 0 | 0 | 0 | 0 | 0 | 3 | 0.00 | 1.00 | 0.00 | 1.00 |
| sp\|Q8K310\|MATR3_MOUSE Matrin-3 OS=Mus musculus GN=Matr3 PE=1 SV=1 | 0.074 | 0.1 | 5 | 2 | 0 | 1 | 0 | 0 | 2.33 | 0.33 | 1.45 | 0.33 |
| sp\|Q5E958\|RS8_BOVIN 40S ribosomal protein S8 OS=Bos taurus GN=RPS8 PE=2 SV=3 | 0.074 | 0.1 | 6 | 1 | 0 | 0 | 0 | 1 | 2.33 | 0.33 | 1.86 | 0.33 |
| sp\|Q8CGY6\|UN45B_MOUSE Protein unc-45 homolog B OS=Mus musculus GN=Unc45b PE=1 SV=1 | 0.075 | 0.3 | 12 | 1 | 0 | 2 | 1 | 1 | 4.33 | 1.33 | 3.84 | 0.33 |
| sp\|A6YRY8\|RSSA_SHEEP 40S ribosomal protein SA OS=Ovis aries GN=RPSA PE=2 SV=1 | 0.075 | 4 | 10 | 1 | 1 | 2 | 1 | 0 | 4.00 | 1.00 | 3.00 | 0.58 |
| sp\|P02468\|LAMC1_MOUSE Laminin subunit gamma-1 OS=Mus musculus GN=Lamc1 PE=1 SV=2 | 0.076 | 1.1 | 19 | 10 | 13 | 19 | 16 | 11 | 13.88 | 15.20 | 2.65 | 2.32 |
| sp\|P26284\|ODPA_RAT Pyruvate dehydrogenase E1 component subunit alpha, somatic form, mitochondrial OS=Rattus norvegicus GN=Pdha1 PE=1 SV=2 | 0.077 | 3.2 | 11 | 3 | 2 | 3 | 0 | 2 | 5.33 | 1.67 | 2.85 | 0.88 |
| sp\|Q9D0E1\|HNRPM_MOUSE Heterogeneous nuclear ribonucleoprotein M OS=Mus musculus GN=Hnrnpm PE=1 SV=3 | 0.078 | 0.3 | 7 | 4 | 0 | 2 | 0 | 1 | 3.67 | 1.00 | 2.03 | 0.58 |
| Cluster of sp\|Q61598\|GDIB_MOUSE Rab GDP dissociation inhibitor beta OS=Mus musculus GN=Gdi2 PE=1 SV=1 | 0.078 | 0.2 | 8 | 1 | 0 | 0 | 1 | 1 | 2.92 | 0.67 | 2.44 | 0.33 |
| Cluster of sp\|P17182\|ENOA_MOUSE Alpha-enolase OS=Mus musculus GN=Eno1 PE=1 SV=3 | 0.081 | 1.1 | 32 | 3 | 4 | 24 | 10 | 8 | 13.15 | 13.96 | 9.24 | 5.11 |
| sp\|A9UMV8\|H2AJ_RAT Histone H2A.J OS=Rattus norvegicus GN=H2afj PE=2 SV=1 | 0.082 | INF | 0 | 0 | 0 | 3 | 0 | 0 | 0.00 | 1.00 | 0.00 | 1.00 |
| sp\|P49702\|ARF5_CHICK ADP-ribosylation factor 5 OS=Gallus gallus GN=ARF5 PE=2 SV=2 | 0.082 | INF | 0 | 0 | 0 | 3 | 0 | 0 | 0.00 | 1.00 | 0.00 | 1.00 |
| Cluster of sp\|Q03265\|ATPA_MOUSE ATP synthase subunit alpha, mitochondrial OS=Mus musculus GN=Atp5a1 PE=1 SV=1 | 0.092 | 0.9 | 60 | 31 | 22 | 50 | 26 | 31 | 38.07 | 35.39 | 11.51 | 7.21 |
| sp\|Q8BWT1\|THIM_MOUSE 3-ketoacyl-CoA thiolase, mitochondrial OS=Mus musculus GN=Acaa2 PE=1 SV=3 | 0.093 | 2.6 | 17 | 3 | 1 | 4 | 1 | 3 | 7.00 | 2.67 | 5.03 | 0.88 |
| sp\|Q921F2\|TADBP_MOUSE TAR DNA-binding protein 43 OS=Mus musculus GN=Tardbp PE=1 SV=1 | 0.094 | 7 | 7 | 0 | 0 | 1 | 0 | 0 | 2.33 | 0.33 | 2.33 | 0.33 |
| sp\|Q7M0E3\|DEST_RAT Destrin OS=Rattus norvegicus GN=Dstn PE=1 SV=3 | 0.094 | 7 | 5 | 1 | 1 | 1 | 0 | 0 | 2.33 | 0.33 | 1.33 | 0.33 |
| sp\|Q8BMF4\|ODP2_MOUSE Dihydrolipoyllysine-residue acetyltransferase component of pyruvate dehydrogenase complex, mitochondrial OS=Mus musculus GN=Dlat PE=1 SV=2 | 0.094 | 7 | 6 | 0 | 1 | 1 | 0 | 0 | 2.33 | 0.33 | 1.86 | 0.33 |
| Cluster of sp\|P68139\|ACTS_CHICK Actin, alpha skeletal muscle OS=Gallus gallus GN=ACTA1 PE=1 SV=1 [27] | 0.096 | 1 | 41 | 28 | 11 | 39 | 17 | 20 | 26.49 | 25.29 | 8.46 | 7.00 |
| sp\|O13019\|RS12_ORENI 40S ribosomal protein S12 OS=Oreochromis niloticus GN=rps12 PE=2 SV=3 | 0.097 | 0.2 | 1 | 0 | 0 | 2 | 0 | 2 | 0.33 | 1.33 | 0.33 | 0.67 |
| sp\|Q9CZ13\|QCR1_MOUSE Cytochrome b-c1 complex subunit 1, mitochondrial OS=Mus musculus GN=Uqcrc1 PE=1 SV=2 | 0.098 | 0.4 | 12 | 1 | 1 | 2 | 1 | 2 | 4.67 | 1.67 | 3.67 | 0.33 |
| Cluster of sp\|O55143\|AT2A2_MOUSE Sarcoplasmic/endoplasmic reticulum calcium ATPase 2 OS=Mus musculus GN=Atp2a2 PE=1 SV=2 | 0.1 | 0.6 | 32 | 8 | 4 | 12 | 7 | 6 | 14.44 | 8.15 | 8.61 | 1.85 |
| sp\|Q01205\|ODO2_RAT Dihydrolipoyllysine-residue succinyltransferase component of 2-oxoglutarate dehydrogenase complex, mitochondrial OS=Rattus norvegicus GN=Dlst PE=1 SV=2 | 0.1 | 1.6 | 4 | 0 | 3 | 6 | 3 | 2 | 2.33 | 3.67 | 1.20 | 1.20 |
| sp\|P23116\|EIF3A_MOUSE Eukaryotic translation initiation factor 3 subunit A OS=Mus musculus GN=Eif3a PE=1 SV=5 | 0.1 | 0 | 4 | 0 | 0 | 0 | 0 | 0 | 1.33 | 0.00 | 1.33 | 0.00 |
| Cluster of sp\|P47857\|PFKAM_MOUSE ATP-dependent 6-phosphofructokinase, muscle type OS=Mus musculus GN=Pfkm PE=1 SV=3 | 0.1 | 0 | 4 | 0 | 0 | 0 | 0 | 0 | 1.50 | 0.00 | 1.50 | 0.00 |
| sp\|F1LQ48\|HNRPL_RAT Heterogeneous nuclear ribonucleoprotein L OS=Rattus norvegicus GN=Hnrnpl PE=1 SV=2 | 0.1 | 0 | 4 | 0 | 0 | 0 | 0 | 0 | 1.33 | 0.00 | 1.33 | 0.00 |
| sp\|F1LNJ2\|U520_RAT U5 small nuclear ribonucleoprotein 200 kDa helicase OS=Rattus norvegicus GN=Snrnp200 PE=1 SV=1 | 0.1 | 0 | 4 | 0 | 0 | 0 | 0 | 0 | 1.33 | 0.00 | 1.33 | 0.00 |
| sp\|O88477\|IF2B1_MOUSE Insulin-like growth factor 2 mRNA-binding protein 1 OS=Mus musculus GN=Igf2bp1 PE=1 SV=1 | 0.1 | 0 | 4 | 0 | 0 | 0 | 0 | 0 | 1.33 | 0.00 | 1.33 | 0.00 |
| sp\|Q6AYK8\|EIF3D_RAT Eukaryotic translation initiation factor 3 subunit D OS=Rattus norvegicus GN=Eif3d PE=1 SV=1 | 0.1 | 0 | 3 | 1 | 0 | 0 | 0 | 0 | 1.33 | 0.00 | 0.88 | 0.00 |
| sp\|Q6ZQ73\|CAND2_MOUSE Cullin-associated NEDD8-dissociated protein 2 OS=Mus musculus GN=Cand2 PE=1 SV=2 | 0.1 | 0 | 4 | 0 | 0 | 0 | 0 | 0 | 1.33 | 0.00 | 1.33 | 0.00 |
| sp\|P05202\|AATM_MOUSE Aspartate aminotransferase, mitochondrial OS=Mus musculus GN=Got2 PE=1 SV=1 | 0.1 | 0 | 4 | 0 | 0 | 0 | 0 | 0 | 1.33 | 0.00 | 1.33 | 0.00 |
| sp\|P26231\|CTNA1_MOUSE Catenin alpha-1 OS=Mus musculus GN=Ctnna1 PE=1 SV=1 | 0.1 | 0 | 4 | 0 | 0 | 0 | 0 | 0 | 1.33 | 0.00 | 1.33 | 0.00 |
| sp\|Q8CGK3\|LONM_MOUSE Lon protease homolog, mitochondrial OS=Mus musculus GN=Lonp1 PE=1 SV=2 | 0.1 | 0 | 4 | 0 | 0 | 0 | 0 | 0 | 1.33 | 0.00 | 1.33 | 0.00 |
| sp\|Q8BFR5\|EFTU_MOUSE Elongation factor Tu, mitochondrial OS=Mus musculus GN=Tufm PE=1 SV=1 | 0.1 | 0 | 4 | 0 | 0 | 0 | 0 | 0 | 1.33 | 0.00 | 1.33 | 0.00 |
| sp\|Q8K2B3\|SDHA_MOUSE Succinate dehydrogenase [ubiquinone] flavoprotein subunit, mitochondrial OS=Mus musculus GN=Sdha PE=1 SV=1 | 0.1 | 0 | 4 | 0 | 0 | 0 | 0 | 0 | 1.33 | 0.00 | 1.33 | 0.00 |
| sp\|P35222\|CTNB1_HUMAN Catenin beta-1 OS=Homo sapiens GN=CTNNB1 PE=1 SV=1 | 0.1 | 0 | 4 | 0 | 0 | 0 | 0 | 0 | 1.48 | 0.00 | 1.48 | 0.00 |
| sp\|P31151\|S10A7_HUMAN Protein S100-A7 OS=Homo sapiens GN=S100A7 PE=1 SV=4 | 0.1 | 0 | 0 | 4 | 0 | 0 | 0 | 0 | 1.33 | 0.00 | 1.33 | 0.00 |
| sp\|Q8BH04\|PCKGM_MOUSE Phosphoenolpyruvate carboxykinase [GTP], mitochondrial OS=Mus musculus GN=Pck2 PE=1 SV=1 | 0.1 | 0 | 4 | 0 | 0 | 0 | 0 | 0 | 1.33 | 0.00 | 1.33 | 0.00 |
| sp\|Q9CWJ9\|PUR9_MOUSE Bifunctional purine biosynthesis protein PURH OS=Mus musculus GN=Atic PE=1 SV=2 | 0.1 | 0 | 4 | 0 | 0 | 0 | 0 | 0 | 1.33 | 0.00 | 1.33 | 0.00 |
| Cluster of sp\|P49717\|MCM4_MOUSE DNA replication licensing factor MCM4 OS=Mus musculus GN=Mcm4 PE=1 SV=1 | 0.1 | 0 | 4 | 0 | 0 | 0 | 0 | 0 | 1.33 | 0.00 | 1.33 | 0.00 |
| sp\|P61158\|ARP3_HUMAN Actin-related protein 3 OS=Homo sapiens GN=ACTR3 PE=1 SV=3 | 0.1 | 0 | 4 | 0 | 0 | 0 | 0 | 0 | 1.33 | 0.00 | 1.33 | 0.00 |
| sp\|Q8CG48\|SMC2_MOUSE Structural maintenance of chromosomes protein 2 OS=Mus musculus GN=Smc2 PE=1 SV=2 | 0.1 | 0 | 4 | 0 | 0 | 0 | 0 | 0 | 1.33 | 0.00 | 1.33 | 0.00 |
| sp\|O95782\|AP2A1_HUMAN AP-2 complex subunit alpha-1 OS=Homo sapiens GN=AP2A1 PE=1 SV=3 | 0.1 | 0 | 4 | 0 | 0 | 0 | 0 | 0 | 1.33 | 0.00 | 1.33 | 0.00 |
| sp\|Q9DB77\|QCR2_MOUSE Cytochrome b-c1 complex subunit 2, mitochondrial OS=Mus musculus GN=Uqcrc2 PE=1 SV=1 | 0.1 | 0 | 4 | 0 | 0 | 0 | 0 | 0 | 1.33 | 0.00 | 1.33 | 0.00 |
| sp\|P00558\|PGK1_HUMAN Phosphoglycerate kinase 1 OS=Homo sapiens GN=PGK1 PE=1 SV=3 | 0.1 | 0 | 4 | 0 | 0 | 0 | 0 | 0 | 1.33 | 0.00 | 1.33 | 0.00 |
| sp\|O60506\|HNRPQ_HUMAN Heterogeneous nuclear ribonucleoprotein Q OS=Homo sapiens GN=SYNCRIP PE=1 SV=2 | 0.1 | 0 | 4 | 0 | 0 | 0 | 0 | 0 | 1.30 | 0.00 | 1.30 | 0.00 |
| sp\|P05109\|S10A8_HUMAN Protein S100-A8 OS=Homo sapiens GN=S100A8 PE=1 SV=1 | 0.1 | 0 | 0 | 4 | 0 | 0 | 0 | 0 | 1.33 | 0.00 | 1.33 | 0.00 |
| sp\|Q8BFR5\|EFTU_MOUSE Elongation factor Tu, mitochondrial OS=Mus musculus GN=Tufm PE=1 SV=1 | 0.1 | 4.5 | 7 | 1 | 1 | 1 | 0 | 1 | 3.00 | 0.67 | 2.00 | 0.33 |
| sp\|P50213\|IDH3A_HUMAN Isocitrate dehydrogenase [NAD] subunit alpha, mitochondrial OS=Homo sapiens GN=IDH3A PE=1 SV=1 | 0.1 | 4.5 | 8 | 1 | 0 | 0 | 1 | 1 | 3.00 | 0.67 | 2.52 | 0.33 |
| sp\|P11499\|HS90B_MOUSEHeat shock protein HSP 90-beta OS=Mus musculus GN=Hsp90ab1 PE=1 SV=3 | 0.11 | 0.7 | 103 | 30 | 18 | 51 | 23 | 25 | 50.20 | 33.05 | 26.46 | 9.12 |
| Cluster of sp\|P06761\|GRP78_RAT 78 kDa glucose-regulated protein OS=Rattus norvegicus GN=Hspa5 PE=1 SV=1 | 0.11 | 1 | 24 | 7 | 9 | 21 | 10 | 7 | 13.18 | 12.71 | 5.57 | 4.30 |
| sp\|P08113\|ENPL_MOUSE Endoplasmin OS=Mus musculus GN=Hsp90b1 PE=1 SV=2 | 0.11 | 0.7 | 5 | 2 | 1 | 7 | 1 | 3 | 2.65 | 3.79 | 1.18 | 1.63 |
| sp\|P13413\|TNNI1_RAT Troponin I, slow skeletal muscle OS=Rattus norvegicus GN=Tnni1 PE=1 SV=2 | 0.11 | 0.7 | 2 | 2 | 2 | 3 | 3 | 3 | 2.00 | 3.00 | 0.00 | 0.00 |
| sp\|P35700\|PRDX1_MOUSE Peroxiredoxin-1 OS=Mus musculus GN=Prdx1 PE=1 SV=1 | 0.11 | 0.6 | 3 | 0 | 0 | 5 | 0 | 0 | 1.10 | 1.77 | 1.10 | 1.77 |
| sp\|A4ITJ9\|GLYA_GEOTN Serine hydroxymethyltransferase OS=Geobacillus thermodenitrificans (strain NG80-2) GN=glyA PE=3 SV=1 | 0.11 | 0.4 | 1 | 0 | 1 | 1 | 2 | 2 | 0.67 | 1.67 | 0.33 | 0.33 |
| sp\|P36604\|GRP78_SCHPO 78 kDa glucose-regulated protein homolog OS=Schizosaccharomyces pombe (strain 972 / ATCC 24843) GN=bip1 PE=3 SV=2 | 0.11 | 0.5 | 1 | 1 | 0 | 0 | 3 | 1 | 0.78 | 1.45 | 0.39 | 0.93 |
| sp\|P80317\|TCPZ_MOUSE T-complex protein 1 subunit zeta OS=Mus musculus GN=Cct6a PE=1 SV=3 | 0.12 | 0.4 | 9 | 3 | 3 | 3 | 1 | 2 | 5.00 | 2.00 | 2.00 | 0.58 |
| sp\|Q8BU30\|SYIC_MOUSE Isoleucine--tRNA ligase, cytoplasmic OS=Mus musculus GN=Iars PE=1 SV=2 | 0.12 | 0.2 | 6 | 0 | 0 | 0 | 0 | 1 | 2.00 | 0.33 | 2.00 | 0.33 |
| sp\|Q91YT0\|NDUV1_MOUSE NADH dehydrogenase [ubiquinone] flavoprotein 1, mitochondrial OS=Mus musculus GN=Ndufv1 PE=1 SV=1 | 0.12 | 0.2 | 6 | 0 | 0 | 1 | 0 | 0 | 2.00 | 0.33 | 2.00 | 0.33 |
| sp\|P14094\|AT1B1_MOUSE Sodium/potassium-transporting ATPase subunit beta-1 OS=Mus musculus GN=Atp1b1 PE=1 SV=1 | 0.12 | 0.2 | 4 | 1 | 1 | 0 | 1 | 0 | 2.00 | 0.33 | 1.00 | 0.33 |
| sp\|A3M7X8\|PUR5_ACIBT Phosphoribosylformylglycinamidine cyclo-ligase OS=Acinetobacter baumannii (strain ATCC 17978 / CIP 53.77 / LMG 1025 / NCDC KC755 / 5377) GN=purM PE=3 SV=2 | 0.12 | 0.6 | 0 | 1 | 3 | 2 | 2 | 3 | 1.33 | 2.33 | 0.88 | 0.33 |
| sp\|O08807\|PRDX4_MOUSE Peroxiredoxin-4 OS=Mus musculus GN=Prdx4 PE=1 SV=1 | 0.12 | 0.5 | 2 | 1 | 0 | 4 | 1 | 1 | 1.23 | 2.36 | 0.68 | 1.01 |
| sp\|Q07417\|ACADS_MOUSE Short-chain specific acyl-CoA dehydrogenase, mitochondrial OS=Mus musculus GN=Acads PE=1 SV=2 | 0.12 | INF | 3 | 0 | 1 | 0 | 0 | 0 | 1.33 | 0.00 | 0.88 | 0.00 |
| sp\|O35501\|GRP75_CRIGR Stress-70 protein, mitochondrial OS=Cricetulus griseus GN=HSPA9 PE=2 SV=1 | 0.12 | INF | 4 | 0 | 0 | 0 | 0 | 0 | 1.33 | 0.00 | 1.33 | 0.00 |
| sp\|Q91YT0\|NDUV1_MOUSE NADH dehydrogenase [ubiquinone] flavoprotein 1, mitochondrial OS=Mus musculus GN=Ndufv1 PE=1 SV=1 | 0.12 | INF | 4 | 0 | 0 | 0 | 0 | 0 | 1.33 | 0.00 | 1.33 | 0.00 |
| sp\|Q9DBH5\|LMAN2_MOUSE Vesicular integral-membrane protein VIP36 OS=Mus musculus GN=Lman2 PE=1 SV=2 | 0.12 | INF | 4 | 0 | 0 | 0 | 0 | 0 | 1.33 | 0.00 | 1.33 | 0.00 |
| sp\|P27659\|RL3_MOUSE 60S ribosomal protein L3 OS=Mus musculus GN=Rpl3 PE=1 SV=3 | 0.12 | INF | 4 | 0 | 0 | 0 | 0 | 0 | 1.33 | 0.00 | 1.33 | 0.00 |
| sp\|P46638\|RB11B_MOUSE Ras-related protein Rab-11B OS=Mus musculus GN=Rab11b PE=1 SV=3 | 0.12 | INF | 4 | 0 | 0 | 0 | 0 | 0 | 1.33 | 0.00 | 1.33 | 0.00 |
| sp\|O55126\|NIPS2_MOUSE Protein NipSnap homolog 2 OS=Mus musculus GN=Nipsnap2 PE=1 SV=1 | 0.12 | INF | 4 | 0 | 0 | 0 | 0 | 0 | 1.33 | 0.00 | 1.33 | 0.00 |
| sp\|Q04857\|CO6A1_MOUSE Collagen alpha-1(VI) chain OS=Mus musculus GN=Col6a1 PE=1 SV=1 | 0.13 | 2.5 | 2 | 0 | 0 | 2 | 1 | 2 | 0.67 | 1.67 | 0.67 | 0.33 |
| Cluster of sp\|P04264\|K2C1_HUMAN Keratin, type II cytoskeletal 1 OS=Homo sapiens GN=KRT1 PE=1 SV=6 | 0.13 | 0.9 | 56 | 47 | 65 | 40 | 53 | 55 | 55.74 | 49.09 | 5.16 | 4.62 |
| Cluster of sp\|Q9JMH9\|MY18A_MOUSE Unconventional myosin-XVIIIa OS=Mus musculus GN=Myo18a PE=1 SV=2 | 0.13 | 0.4 | 9 | 5 | 4 | 7 | 0 | 1 | 5.93 | 2.63 | 1.50 | 2.16 |
| sp\|O54724\|PTRF_MOUSE Polymerase I and transcript release factor OS=Mus musculus GN=Ptrf PE=1 SV=1 | 0.13 | 2.5 | 2 | 0 | 0 | 3 | 2 | 0 | 0.67 | 1.67 | 0.67 | 0.88 |
| Cluster of sp\|P35908\|K22E_HUMAN Keratin, type II cytoskeletal 2 epidermal OS=Homo sapiens GN=KRT2 PE=1 SV=2 | 0.14 | 1 | 19 | 39 | 4 | 13 | 15 | 31 | 20.68 | 19.67 | 10.11 | 5.52 |
| sp\|Q5E958\|RS8_BOVIN 40S ribosomal protein S8 OS=Bos taurus GN=RPS8 PE=2 SV=3 | 0.14 | 2.2 | 20 | 6 | 2 | 4 | 5 | 4 | 9.33 | 4.33 | 5.46 | 0.33 |
| sp\|P45952\|ACADM_MOUSE Medium-chain specific acyl-CoA dehydrogenase, mitochondrial OS=Mus musculus GN=Acadm PE=1 SV=1 | 0.14 | 3 | 10 | 2 | 0 | 3 | 1 | 0 | 4.00 | 1.33 | 3.06 | 0.88 |
| sp\|Q8QZT1\|THIL_MOUSE Acetyl-CoA acetyltransferase, mitochondrial OS=Mus musculus GN=Acat1 PE=1 SV=1 | 0.14 | 6 | 6 | 0 | 0 | 1 | 0 | 0 | 2.00 | 0.33 | 2.00 | 0.33 |
| sp\|Q7VKZ4\|GLNE_HAEDU Bifunctional glutamine synthetase adenylyltransferase/adenylyl-removing enzyme OS=Haemophilus ducreyi (strain 35000HP / ATCC 700724) GN=glnE PE=3 SV=1 | 0.14 | 6 | 1 | 2 | 3 | 0 | 0 | 1 | 2.00 | 0.33 | 0.58 | 0.33 |
| sp\|P97927\|LAMA4_MOUSE Laminin subunit alpha-4 OS=Mus musculus GN=Lama4 PE=1 SV=2 | 0.15 | 1.6 | 2 | 2 | 1 | 4 | 2 | 2 | 1.67 | 2.67 | 0.33 | 0.67 |
| sp\|P51881\|ADT2_MOUSE ADP/ATP translocase 2 OS=Mus musculus GN=Slc25a5 PE=1 SV=3 | 0.15 | 0.5 | 19 | 7 | 1 | 4 | 5 | 5 | 9.13 | 4.51 | 5.23 | 0.33 |
| sp\|P14211\|CALR_MOUSE Calreticulin OS=Mus musculus GN=Calr PE=1 SV=1 | 0.15 | 1.4 | 6 | 0 | 2 | 7 | 2 | 2 | 2.67 | 3.67 | 1.76 | 1.67 |
| sp\|P00762\|TRY1_RAT Anionic trypsin-1 OS=Rattus norvegicus GN=Prss1 PE=1 SV=1 | 0.15 | 0.8 | 2 | 3 | 4 | 4 | 3 | 4 | 3.00 | 3.67 | 0.58 | 0.33 |
| sp\|Q9XSI3\|RL10_BOVIN 60S ribosomal protein L10 OS=Bos taurus GN=RPL10 PE=2 SV=4 | 0.15 | 0.8 | 7 | 3 | 0 | 7 | 2 | 3 | 3.33 | 4.00 | 2.03 | 1.53 |
| sp\|O60814\|H2B1K_HUMAN Histone H2B type 1-K OS=Homo sapiens GN=HIST1H2BK PE=1 SV=3 | 0.15 | 0.8 | 7 | 0 | 3 | 7 | 4 | 1 | 3.32 | 3.98 | 2.02 | 1.73 |
| Cluster of sp\|P17844\|DDX5_HUMAN Probable ATP-dependent RNA helicase DDX5 OS=Homo sapiens GN=DDX5 PE=1 SV=1 | 0.15 | 5.1 | 6 | 3 | 0 | 2 | 0 | 0 | 2.90 | 0.57 | 1.73 | 0.57 |
| sp\|P49242\|RS3A_RAT 40S ribosomal protein S3a OS=Rattus norvegicus GN=Rps3a PE=1 SV=2 | 0.15 | 3.3 | 9 | 0 | 1 | 1 | 1 | 1 | 3.33 | 1.00 | 2.85 | 0.00 |
| sp\|P11983\|TCPA_MOUSE T-complex protein 1 subunit alpha OS=Mus musculus GN=Tcp1 PE=1 SV=3 | 0.16 | 0.5 | 18 | 8 | 5 | 8 | 4 | 5 | 10.33 | 5.67 | 3.93 | 1.20 |
| sp\|P80318\|TCPG_MOUSE T-complex protein 1 subunit gamma OS=Mus musculus GN=Cct3 PE=1 SV=1 | 0.16 | 0.5 | 16 | 4 | 2 | 4 | 4 | 3 | 7.33 | 3.67 | 4.37 | 0.33 |
| sp\|P02533\|K1C14_HUMAN Keratin, type I cytoskeletal 14 OS=Homo sapiens GN=KRT14 PE=1 SV=4 | 0.16 | 0.4 | 4 | 4 | 1 | 1 | 0 | 2 | 2.83 | 1.10 | 0.76 | 0.67 |
| sp\|Q9DCT2\|NDUS3_MOUSE NADH dehydrogenase [ubiquinone] iron-sulfur protein 3, mitochondrial OS=Mus musculus GN=Ndufs3 PE=1 SV=2 | 0.17 | 0.8 | 4 | 2 | 1 | 4 | 3 | 2 | 2.33 | 3.00 | 0.88 | 0.58 |
| sp\|P57044\|ILK_CAVPO Integrin-linked protein kinase OS=Cavia porcellus GN=ILK PE=2 SV=1 | 0.18 | 0 | 3 | 0 | 0 | 0 | 0 | 0 | 1.00 | 0.00 | 1.00 | 0.00 |
| Cluster of sp\|Q9JI91\|ACTN2_MOUSE Alpha-actinin-2 OS=Mus musculus GN=Actn2 PE=1 SV=2 (sp\|Q9JI91\|ACTN2_MOUSEAlpha-actinin-2 OS=Mus musculus GN=Actn2 PE=1 SV=2) | 0.18 | 0.7 | 71 | 25 | 15 | 30 | 19 | 25 | 37.27 | 24.54 | 17.34 | 3.10 |
| sp\|P60122\|RUVB1_MOUSE RuvB-like 1 OS=Mus musculus GN=Ruvbl1 PE=1 SV=1 | 0.18 | 0.2 | 5 | 0 | 0 | 1 | 0 | 0 | 1.67 | 0.33 | 1.67 | 0.33 |
| sp\|Q5I0G4\|GARS_RAT Glycine--tRNA ligase (Fragment) OS=Rattus norvegicus GN=Gars PE=1 SV=1 | 0.18 | 0.2 | 4 | 1 | 0 | 1 | 0 | 0 | 1.67 | 0.33 | 1.20 | 0.33 |
| sp\|Q9ULV4\|COR1C_HUMAN Coronin-1C OS=Homo sapiens GN=CORO1C PE=1 SV=1 | 0.18 | 0.2 | 4 | 1 | 0 | 0 | 1 | 0 | 1.67 | 0.33 | 1.20 | 0.33 |
| sp\|P14685\|PSMD3_MOUSE 26S proteasome non-ATPase regulatory subunit 3 OS=Mus musculus GN=Psmd3 PE=1 SV=3 | 0.18 | 0.2 | 5 | 0 | 0 | 1 | 0 | 0 | 1.67 | 0.33 | 1.67 | 0.33 |
| sp\|P19096\|FAS_MOUSE Fatty acid synthase OS=Mus musculus GN=Fasn PE=1 SV=2 | 0.18 | 0 | 3 | 0 | 0 | 0 | 0 | 0 | 1.00 | 0.00 | 1.00 | 0.00 |
| sp\|Q8BMS1\|ECHA_MOUSE Trifunctional enzyme subunit alpha, mitochondrial OS=Mus musculus GN=Hadha PE=1 SV=1 | 0.18 | 0 | 3 | 0 | 0 | 0 | 0 | 0 | 1.00 | 0.00 | 1.00 | 0.00 |
| sp\|P06702\|S10A9_HUMAN Protein S100-A9 OS=Homo sapiens GN=S100A9 PE=1 SV=1 | 0.18 | 0 | 0 | 3 | 0 | 0 | 0 | 0 | 1.00 | 0.00 | 1.00 | 0.00 |
| sp\|Q61316\|HSP74_MOUSE Heat shock 70 kDa protein 4 OS=Mus musculus GN=Hspa4 PE=1 SV=1 | 0.18 | 0 | 3 | 0 | 0 | 0 | 0 | 0 | 1.00 | 0.00 | 1.00 | 0.00 |
| sp\|P30999\|CTND1_MOUSE Catenin delta-1 OS=Mus musculus GN=Ctnnd1 PE=1 SV=2 | 0.18 | 0 | 2 | 1 | 0 | 0 | 0 | 0 | 1.00 | 0.00 | 0.58 | 0.00 |
| sp\|Q64737\|PUR2_MOUSE Trifunctional purine biosynthetic protein adenosine-3 OS=Mus musculus GN=Gart PE=1 SV=3 | 0.18 | 0 | 3 | 0 | 0 | 0 | 0 | 0 | 1.00 | 0.00 | 1.00 | 0.00 |
| sp\|O09106\|HDAC1_MOUSE Histone deacetylase 1 OS=Mus musculus GN=Hdac1 PE=1 SV=1 | 0.18 | 0 | 3 | 0 | 0 | 0 | 0 | 0 | 1.00 | 0.00 | 1.00 | 0.00 |
| sp\|P70698\|PYRG1_MOUSE CTP synthase 1 OS=Mus musculus GN=Ctps1 PE=1 SV=2 | 0.18 | 0.2 | 5 | 0 | 0 | 1 | 0 | 0 | 1.67 | 0.33 | 1.67 | 0.33 |
| sp\|Q9DC61\|MPPA_MOUSE Mitochondrial-processing peptidase subunit alpha OS=Mus musculus GN=Pmpca PE=1 SV=1 | 0.18 | 0 | 3 | 0 | 0 | 0 | 0 | 0 | 1.00 | 0.00 | 1.00 | 0.00 |
| sp\|B2RQC6\|PYR1_MOUSE CAD protein OS=Mus musculus GN=Cad PE=1 SV=1 | 0.18 | 0 | 3 | 0 | 0 | 0 | 0 | 0 | 1.00 | 0.00 | 1.00 | 0.00 |
| sp\|Q11011\|PSA_MOUSE Puromycin-sensitive aminopeptidase OS=Mus musculus GN=Npepps PE=1 SV=2 | 0.18 | 0 | 3 | 0 | 0 | 0 | 0 | 0 | 1.00 | 0.00 | 1.00 | 0.00 |
| sp\|Q99MN1\|SYK_MOUSE Lysine--tRNA ligase OS=Mus musculus GN=Kars PE=1 SV=1 | 0.18 | 0 | 3 | 0 | 0 | 0 | 0 | 0 | 1.00 | 0.00 | 1.00 | 0.00 |
| sp\|O08629\|TIF1B_RAT Transcription intermediary factor 1-beta OS=Rattus norvegicus GN=Trim28 PE=1 SV=2 | 0.18 | 0 | 3 | 0 | 0 | 0 | 0 | 0 | 1.00 | 0.00 | 1.00 | 0.00 |
| sp\|P0CE43\|GRB10_RAT Growth factor receptor-bound protein 10 OS=Rattus norvegicus GN=Grb10 PE=3 SV=1 | 0.18 | 0 | 3 | 0 | 0 | 0 | 0 | 0 | 1.00 | 0.00 | 1.00 | 0.00 |
| sp\|P53395\|ODB2_MOUSE Lipoamide acyltransferase component of branched-chain alpha-keto acid dehydrogenase complex, mitochondrial OS=Mus musculus GN=Dbt PE=1 SV=2 | 0.18 | 0 | 3 | 0 | 0 | 0 | 0 | 0 | 1.00 | 0.00 | 1.00 | 0.00 |
| sp\|Q9JLI8\|SART3_MOUSE Squamous cell carcinoma antigen recognized by T-cells 3 OS=Mus musculus GN=Sart3 PE=1 SV=1 | 0.18 | 0 | 3 | 0 | 0 | 0 | 0 | 0 | 1.00 | 0.00 | 1.00 | 0.00 |
| sp\|Q9WUA2\|SYFB_MOUSE Phenylalanine--tRNA ligase beta subunit OS=Mus musculus GN=Farsb PE=1 SV=2 | 0.18 | 0 | 3 | 0 | 0 | 0 | 0 | 0 | 1.00 | 0.00 | 1.00 | 0.00 |
| sp\|O97594\|SMC3_BOVIN Structural maintenance of chromosomes protein 3 OS=Bos taurus GN=SMC3 PE=1 SV=1 | 0.18 | 0 | 3 | 0 | 0 | 0 | 0 | 0 | 1.00 | 0.00 | 1.00 | 0.00 |
| sp\|Q61941\|NNTM_MOUSE NAD(P) transhydrogenase, mitochondrial OS=Mus musculus GN=Nnt PE=1 SV=2 | 0.18 | 0 | 3 | 0 | 0 | 0 | 0 | 0 | 1.00 | 0.00 | 1.00 | 0.00 |
| sp\|O88685\|PRS6A_MOUSE 26S protease regulatory subunit 6A OS=Mus musculus GN=Psmc3 PE=1 SV=2 | 0.18 | 0 | 3 | 0 | 0 | 0 | 0 | 0 | 1.00 | 0.00 | 1.00 | 0.00 |
| sp\|Q60749\|KHDR1_MOUSE KH domain-containing, RNA-binding, signal transduction-associated protein 1 OS=Mus musculus GN=Khdrbs1 PE=1 SV=2 | 0.18 | 0 | 2 | 1 | 0 | 0 | 0 | 0 | 1.00 | 0.00 | 0.58 | 0.00 |
| sp\|P18298\|METK2_RAT S-adenosylmethionine synthase isoform type-2 OS=Rattus norvegicus GN=Mat2a PE=1 SV=1 | 0.18 | 0 | 3 | 0 | 0 | 0 | 0 | 0 | 1.00 | 0.00 | 1.00 | 0.00 |
| sp\|Q3V3R1\|C1TM_MOUSE Monofunctional C1-tetrahydrofolate synthase, mitochondrial OS=Mus musculus GN=Mthfd1l PE=1 SV=2 | 0.18 | 0 | 3 | 0 | 0 | 0 | 0 | 0 | 1.00 | 0.00 | 1.00 | 0.00 |
| sp\|Q01469\|FABP5_HUMAN Fatty acid-binding protein, epidermal OS=Homo sapiens GN=FABP5 PE=1 SV=3 | 0.18 | 0 | 1 | 2 | 0 | 0 | 0 | 0 | 1.00 | 0.00 | 0.58 | 0.00 |
| sp\|P43034\|LIS1_HUMAN Platelet-activating factor acetylhydrolase IB subunit alpha OS=Homo sapiens GN=PAFAH1B1 PE=1 SV=2 | 0.18 | 0 | 3 | 0 | 0 | 0 | 0 | 0 | 1.00 | 0.00 | 1.00 | 0.00 |
| sp\|O08528\|HXK2_MOUSE Hexokinase-2 OS=Mus musculus GN=Hk2 PE=1 SV=1 | 0.18 | 0 | 3 | 0 | 0 | 0 | 0 | 0 | 1.16 | 0.00 | 1.16 | 0.00 |
| sp\|O35814\|STIP1_RAT Stress-induced-phosphoprotein 1 OS=Rattus norvegicus GN=Stip1 PE=1 SV=1 | 0.18 | 0 | 3 | 0 | 0 | 0 | 0 | 0 | 1.00 | 0.00 | 1.00 | 0.00 |
| sp\|Q5XHY5\|SYTC_RAT Threonine--tRNA ligase, cytoplasmic OS=Rattus norvegicus GN=Tars PE=1 SV=1 | 0.18 | 0 | 3 | 0 | 0 | 0 | 0 | 0 | 1.00 | 0.00 | 1.00 | 0.00 |
| sp\|Q8R2Y2\|MUC18_MOUSE Cell surface glycoprotein MUC18 OS=Mus musculus GN=Mcam PE=1 SV=1 | 0.18 | 0 | 3 | 0 | 0 | 0 | 0 | 0 | 1.00 | 0.00 | 1.00 | 0.00 |
| sp\|Q8JZN5\|ACAD9_MOUSE Acyl-CoA dehydrogenase family member 9, mitochondrial OS=Mus musculus GN=Acad9 PE=1 SV=2 | 0.18 | 0 | 3 | 0 | 0 | 0 | 0 | 0 | 1.00 | 0.00 | 1.00 | 0.00 |
| sp\|P42558\|RAN_CHICK GTP-binding nuclear protein Ran OS=Gallus gallus GN=RAN PE=2 SV=1 | 0.18 | 0 | 3 | 0 | 0 | 0 | 0 | 0 | 1.00 | 0.00 | 1.00 | 0.00 |
| sp\|P15650\|ACADL_RAT Long-chain specific acyl-CoA dehydrogenase, mitochondrial OS=Rattus norvegicus GN=Acadl PE=1 SV=1 | 0.18 | 0 | 3 | 0 | 0 | 0 | 0 | 0 | 1.00 | 0.00 | 1.00 | 0.00 |
| sp\|Q99K48\|NONO_MOUSE Non-POU domain-containing octamer-binding protein OS=Mus musculus GN=Nono PE=1 SV=3 | 0.18 | 0 | 3 | 0 | 0 | 0 | 0 | 0 | 1.00 | 0.00 | 1.00 | 0.00 |
| sp\|O08663\|MAP2_MOUSE Methionine aminopeptidase 2 OS=Mus musculus GN=Metap2 PE=1 SV=1 | 0.18 | 0 | 3 | 0 | 0 | 0 | 0 | 0 | 1.00 | 0.00 | 1.00 | 0.00 |
| Cluster of sp\|P63018\|HSP7C_RAT Heat shock cognate 71 kDa protein OS=Rattus norvegicus GN=Hspa8 PE=1 SV=1 | 0.18 | 1.2 | 50 | 15 | 7 | 24 | 21 | 14 | 24.16 | 19.84 | 13.08 | 3.10 |
| Cluster of sp\|P17751\|TPIS_MOUSE Triosephosphate isomerase OS=Mus musculus GN=Tpi1 PE=1 SV=4 | 0.18 | 2.4 | 12 | 0 | 1 | 4 | 0 | 1 | 4.33 | 1.78 | 3.84 | 1.31 |
| sp\|P83731\|RL24_HUMAN 60S ribosomal protein L24 OS=Homo sapiens GN=RPL24 PE=1 SV=1 | 0.18 | 0.7 | 2 | 1 | 2 | 3 | 3 | 1 | 1.67 | 2.33 | 0.33 | 0.67 |
| sp\|Q5RC11\|RL11_PONAB 60S ribosomal protein L11 OS=Pongo abelii GN=RPL11 PE=2 SV=3 | 0.18 | 0.7 | 4 | 1 | 0 | 3 | 1 | 3 | 1.67 | 2.33 | 1.20 | 0.67 |
| Cluster of sp\|A9UMV8\|H2AJ_RAT Histone H2A.J OS=Rattus norvegicus GN=H2afj PE=2 SV=1 | 0.18 | 0.7 | 5 | 0 | 0 | 7 | 0 | 0 | 1.67 | 2.33 | 1.67 | 2.33 |
| sp\|P08113\|ENPL_MOUSE Endoplasmin OS=Mus musculus GN=Hsp90b1 PE=1 SV=2 | 0.19 | 0.6 | 37 | 19 | 14 | 16 | 12 | 17 | 23.30 | 14.93 | 7.12 | 1.44 |
| Cluster of sp\|P35908\|K22E_HUMAN Keratin, type II cytoskeletal 2 epidermal OS=Homo sapiens GN=KRT2 PE=1 SV=2 | 0.19 | 1.7 | 23 | 47 | 22 | 17 | 14 | 24 | 30.51 | 18.34 | 8.06 | 2.84 |
| sp\|O19048\|PCBP1_RABIT Poly(rC)-binding protein 1 OS=Oryctolagus cuniculus GN=PCBP1 PE=2 SV=1 | 0.19 | 0.6 | 3 | 0 | 0 | 4 | 0 | 1 | 1.00 | 1.67 | 1.00 | 1.20 |
| sp\|P39656\|OST48_HUMAN Dolichyl-diphosphooligosaccharide--protein glycosyltransferase 48 kDa subunit OS=Homo sapiens GN=DDOST PE=1 SV=4 | 0.19 | 0.6 | 3 | 0 | 0 | 2 | 3 | 0 | 1.00 | 1.67 | 1.00 | 0.88 |
| sp\|Q9QX73\|ARHG9_RAT Rho guanine nucleotide exchange factor 9 OS=Rattus norvegicus GN=Arhgef9 PE=1 SV=1 | 0.19 | 0.3 | 0 | 0 | 1 | 1 | 2 | 0 | 0.33 | 1.00 | 0.33 | 0.58 |
| sp\|Q9DCS9\|NDUBA_MOUSE NADH dehydrogenase [ubiquinone] 1 beta subcomplex subunit 10 OS=Mus musculus GN=Ndufb10 PE=1 SV=3 | 0.19 | 0.3 | 1 | 0 | 0 | 1 | 1 | 1 | 0.33 | 1.00 | 0.33 | 0.00 |
| sp\|P23396\|RS3_HUMAN 40S ribosomal protein S3 OS=Homo sapiens GN=RPS3 PE=1 SV=2 | 0.2 | 0.4 | 6 | 4 | 0 | 2 | 2 | 0 | 3.33 | 1.33 | 1.76 | 0.67 |
| Cluster of sp\|P69897\|TBB5_RAT Tubulin beta-5 chain OS=Rattus norvegicus GN=Tubb5 PE=1 SV=1 | 0.2 | 1.7 | 50 | 16 | 4 | 20 | 14 | 8 | 23.42 | 13.74 | 13.81 | 3.42 |
| sp\|O88569\|ROA2_MOUSE Heterogeneous nuclear ribonucleoproteins A2/B1 OS=Mus musculus GN=Hnrnpa2b1 PE=1 SV=2 | 0.2 | 2 | 14 | 5 | 2 | 3 | 2 | 5 | 6.87 | 3.48 | 3.59 | 0.87 |
| sp\|Q9DB20\|ATPO_MOUSE ATP synthase subunit O, mitochondrial OS=Mus musculus GN=Atp5o PE=1 SV=1 | 0.2 | 0.9 | 8 | 3 | 0 | 5 | 2 | 5 | 3.67 | 4.00 | 2.33 | 1.00 |
| sp\|A5A6N4\|IF4A1_PANTR Eukaryotic initiation factor 4A-I OS=Pan troglodytes GN=EIF4A1 PE=2 SV=1 | 0.2 | 3 | 6 | 2 | 1 | 2 | 0 | 1 | 3.00 | 1.00 | 1.53 | 0.58 |
| sp\|A2ASS6\|TITIN_MOUSE Titin OS=Mus musculus GN=Ttn PE=1 SV=1 | 0.2 | 0.5 | 1 | 1 | 0 | 2 | 0 | 2 | 0.67 | 1.33 | 0.33 | 0.67 |
| sp\|P48774\|GSTM5_MOUSE Glutathione S-transferase Mu 5 OS=Mus musculus GN=Gstm5 PE=1 SV=1 | 0.2 | INF | 3 | 0 | 0 | 0 | 0 | 0 | 1.00 | 0.00 | 1.00 | 0.00 |
| sp\|P11442\|CLH1_RAT Clathrin heavy chain 1 OS=Rattus norvegicus GN=Cltc PE=1 SV=3 | 0.2 | INF | 3 | 0 | 0 | 0 | 0 | 0 | 1.00 | 0.00 | 1.00 | 0.00 |
| sp\|P40142\|TKT_MOUSE Transketolase OS=Mus musculus GN=Tkt PE=1 SV=1 | 0.2 | INF | 3 | 0 | 0 | 0 | 0 | 0 | 1.00 | 0.00 | 1.00 | 0.00 |
| sp\|P06493\|CDK1_HUMAN Cyclin-dependent kinase 1 OS=Homo sapiens GN=CDK1 PE=1 SV=3 | 0.2 | INF | 3 | 0 | 0 | 0 | 0 | 0 | 1.00 | 0.00 | 1.00 | 0.00 |
| sp\|O14979\|HNRDL_HUMAN Heterogeneous nuclear ribonucleoprotein D-like OS=Homo sapiens GN=HNRNPDL PE=1 SV=3 | 0.2 | INF | 3 | 0 | 0 | 0 | 0 | 0 | 1.00 | 0.00 | 1.00 | 0.00 |
| sp\|P32233\|DRG1_MOUSE Developmentally-regulated GTP-binding protein 1 OS=Mus musculus GN=Drg1 PE=1 SV=1 | 0.2 | INF | 3 | 0 | 0 | 0 | 0 | 0 | 1.00 | 0.00 | 1.00 | 0.00 |
| sp\|P50580\|PA2G4_MOUSE Proliferation-associated protein 2G4 OS=Mus musculus GN=Pa2g4 PE=1 SV=3 | 0.2 | INF | 3 | 0 | 0 | 0 | 0 | 0 | 1.00 | 0.00 | 1.00 | 0.00 |
| sp\|Q8BK64\|AHSA1_MOUSE Activator of 90 kDa heat shock protein ATPase homolog 1 OS=Mus musculus GN=Ahsa1 PE=1 SV=2 | 0.2 | INF | 3 | 0 | 0 | 0 | 0 | 0 | 1.00 | 0.00 | 1.00 | 0.00 |
| sp\|P06745\|G6PI_MOUSE Glucose-6-phosphate isomerase OS=Mus musculus GN=Gpi PE=1 SV=4 | 0.2 | INF | 3 | 0 | 0 | 0 | 0 | 0 | 1.00 | 0.00 | 1.00 | 0.00 |
| sp\|P16036\|MPCP_RAT Phosphate carrier protein, mitochondrial OS=Rattus norvegicus GN=Slc25a3 PE=1 SV=1 | 0.2 | INF | 3 | 0 | 0 | 0 | 0 | 0 | 1.00 | 0.00 | 1.00 | 0.00 |
| sp\|P80317\|TCPZ_MOUSE T-complex protein 1 subunit zeta OS=Mus musculus GN=Cct6a PE=1 SV=3 | 0.2 | INF | 3 | 0 | 0 | 0 | 0 | 0 | 1.00 | 0.00 | 1.00 | 0.00 |
| sp\|O35094\|TIM44_RAT Mitochondrial import inner membrane translocase subunit TIM44 OS=Rattus norvegicus GN=Timm44 PE=1 SV=1 | 0.2 | INF | 3 | 0 | 0 | 0 | 0 | 0 | 1.00 | 0.00 | 1.00 | 0.00 |
| sp\|P49813\|TMOD1_MOUSE Tropomodulin-1 OS=Mus musculus GN=Tmod1 PE=1 SV=2 | 0.2 | INF | 3 | 0 | 0 | 0 | 0 | 0 | 1.00 | 0.00 | 1.00 | 0.00 |
| sp\|Q641Y2\|NDUS2_RAT NADH dehydrogenase [ubiquinone] iron-sulfur protein 2, mitochondrial OS=Rattus norvegicus GN=Ndufs2 PE=1 SV=1 | 0.2 | INF | 3 | 0 | 0 | 0 | 0 | 0 | 1.00 | 0.00 | 1.00 | 0.00 |
| sp\|P36552\|HEM6_MOUSE Oxygen-dependent coproporphyrinogen-III oxidase, mitochondrial OS=Mus musculus GN=Cpox PE=1 SV=2 | 0.2 | INF | 3 | 0 | 0 | 0 | 0 | 0 | 1.00 | 0.00 | 1.00 | 0.00 |
| sp\|Q9QZQ8\|H2AY_MOUSE Core histone macro-H2A.1 OS=Mus musculus GN=H2afy PE=1 SV=3 | 0.2 | INF | 3 | 0 | 0 | 0 | 0 | 0 | 1.00 | 0.00 | 1.00 | 0.00 |
| sp\|P01876\|IGHA1_HUMAN Immunoglobulin heavy constant alpha 1 OS=Homo sapiens GN=IGHA1 PE=1 SV=2 | 0.2 | INF | 1 | 2 | 0 | 0 | 0 | 0 | 1.00 | 0.00 | 0.58 | 0.00 |
| sp\|P05201\|AATC_MOUSE Aspartate aminotransferase, cytoplasmic OS=Mus musculus GN=Got1 PE=1 SV=3 | 0.2 | INF | 3 | 0 | 0 | 0 | 0 | 0 | 1.00 | 0.00 | 1.00 | 0.00 |
| sp\|O88712\|CTBP1_MOUSE C-terminal-binding protein 1 OS=Mus musculus GN=Ctbp1 PE=1 SV=2 | 0.2 | INF | 3 | 0 | 0 | 0 | 0 | 0 | 1.00 | 0.00 | 1.00 | 0.00 |
| sp\|Q8VEK3\|HNRPU_MOUSE Heterogeneous nuclear ribonucleoprotein U OS=Mus musculus GN=Hnrnpu PE=1 SV=1 | 0.21 | 2.3 | 10 | 4 | 0 | 5 | 0 | 1 | 4.67 | 2.00 | 2.91 | 1.53 |
| sp\|Q9CX86\|ROA0_MOUSE Heterogeneous nuclear ribonucleoprotein A0 OS=Mus musculus GN=Hnrnpa0 PE=1 SV=1 | 0.21 | 2.3 | 11 | 2 | 1 | 3 | 1 | 2 | 4.67 | 2.00 | 3.18 | 0.58 |
| sp\|Q255Y6\|NQRF_CHLFF Na(+)-translocating NADH-quinone reductase subunit F OS=Chlamydia felis (strain Fe/C-56) GN=nqrF PE=3 SV=1 | 0.21 | 5 | 0 | 2 | 3 | 0 | 0 | 1 | 1.67 | 0.33 | 0.88 | 0.33 |
| sp\|P16125\|LDHB_MOUSE L-lactate dehydrogenase B chain OS=Mus musculus GN=Ldhb PE=1 SV=2 | 0.21 | 5 | 5 | 0 | 0 | 1 | 0 | 0 | 1.67 | 0.33 | 1.67 | 0.33 |
| sp\|P06761\|GRP78_RAT 78 kDa glucose-regulated protein OS=Rattus norvegicus GN=Hspa5 PE=1 SV=1 | 0.22 | 0.7 | 40 | 10 | 11 | 16 | 13 | 11 | 20.42 | 13.46 | 9.86 | 1.45 |
| Cluster of sp\|Q61554\|FBN1_MOUSE Fibrillin-1 OS=Mus musculus GN=Fbn1 PE=1 SV=2 | 0.22 | 0.9 | 22 | 11 | 11 | 22 | 11 | 9 | 14.62 | 13.81 | 3.48 | 3.96 |
| sp\|Q61554\|FBN1_MOUSE Fibrillin-1 OS=Mus musculus GN=Fbn1 PE=1 SV=2 | 0.22 | 0.9 | 22 | 11 | 11 | 22 | 11 | 9 | 14.62 | 13.81 | 3.48 | 3.96 |
| sp\|A7VJC2\|ROA2_RAT Heterogeneous nuclear ribonucleoproteins A2/B1 OS=Rattus norvegicus GN=Hnrnpa2b1 PE=1 SV=1 | 0.22 | 3 | 0 | 1 | 0 | 3 | 0 | 0 | 0.33 | 1.00 | 0.33 | 1.00 |
| sp\|Q6ZWN5\|RS9_MOUSE 40S ribosomal protein S9 OS=Mus musculus GN=Rps9 PE=1 SV=3 | 0.22 | 2.1 | 12 | 5 | 2 | 5 | 0 | 4 | 6.33 | 3.00 | 2.96 | 1.53 |
| sp\|Q91ZJ5\|UGPA_MOUSE UTP--glucose-1-phosphate uridylyltransferase OS=Mus musculus GN=Ugp2 PE=1 SV=3 | 0.23 | 1.5 | 4 | 0 | 0 | 2 | 2 | 2 | 1.33 | 2.00 | 1.33 | 0.00 |
| sp\|Q7TQ48\|SRCA_MOUSE Sarcalumenin OS=Mus musculus GN=Srl PE=1 SV=1 | 0.23 | 1.5 | 3 | 1 | 0 | 2 | 2 | 2 | 1.33 | 2.00 | 0.88 | 0.00 |
| sp\|P46935\|NEDD4_MOUSE E3 ubiquitin-protein ligase NEDD4 OS=Mus musculus GN=Nedd4 PE=1 SV=3 | 0.23 | 0.9 | 7 | 1 | 0 | 3 | 3 | 3 | 2.67 | 3.00 | 2.19 | 0.00 |
| sp\|P41565\|IDHG1_RAT Isocitrate dehydrogenase [NAD] subunit gamma 1, mitochondrial OS=Rattus norvegicus GN=Idh3g PE=1 SV=2 | 0.23 | 2.4 | 12 | 0 | 0 | 2 | 1 | 2 | 4.00 | 1.67 | 4.00 | 0.33 |
| sp\|A5JST6\|RS18_CAPHI 40S ribosomal protein S18 OS=Capra hircus GN=RPS18 PE=2 SV=1 | 0.23 | 0.9 | 3 | 3 | 2 | 3 | 3 | 3 | 2.67 | 3.00 | 0.33 | 0.00 |
| sp\|Q90339\|MYSS_CYPCA Myosin heavy chain, fast skeletal muscle OS=Cyprinus carpio PE=2 SV=2 | 0.24 | 0.9 | 13 | 5 | 10 | 21 | 0 | 5 | 9.34 | 8.83 | 2.51 | 6.45 |
| Cluster of sp\|O70468\|MYPC3_MOUSE Myosin-binding protein C, cardiac-type OS=Mus musculus GN=Mybpc3 PE=1 SV=1 | 0.24 | 0.9 | 7 | 0 | 0 | 6 | 0 | 2 | 2.34 | 2.67 | 2.34 | 1.68 |
| sp\|P35435\|ATPG_RAT ATP synthase subunit gamma, mitochondrial OS=Rattus norvegicus GN=Atp5c1 PE=1 SV=2 | 0.24 | 2 | 9 | 6 | 5 | 3 | 3 | 4 | 6.67 | 3.33 | 1.20 | 0.33 |
| sp\|P00405\|COX2_MOUSE Cytochrome c oxidase subunit 2 OS=Mus musculus GN=Mtco2 PE=1 SV=1 | 0.24 | 0.9 | 4 | 2 | 1 | 2 | 2 | 4 | 2.33 | 2.67 | 0.88 | 0.67 |
| sp\|P30668\|TBB_SCHCO Tubulin beta chain OS=Schizophyllum commune GN=TUB-2 PE=3 SV=1 | 0.25 | 0.4 | 3 | 1 | 2 | 0 | 0 | 2 | 2.02 | 0.77 | 0.32 | 0.77 |
| sp\|Q8VEM8\|MPCP_MOUSE Phosphate carrier protein, mitochondrial OS=Mus musculus GN=Slc25a3 PE=1 SV=1 | 0.25 | 0.3 | 5 | 1 | 0 | 1 | 0 | 1 | 2.00 | 0.67 | 1.53 | 0.33 |
| Cluster of sp\|Q9D0F9\|PGM1_MOUSE Phosphoglucomutase-1 OS=Mus musculus GN=Pgm1 PE=1 SV=4 | 0.25 | 0.3 | 6 | 0 | 0 | 2 | 0 | 0 | 2.00 | 0.60 | 2.00 | 0.60 |
| sp\|P08733\|MLRV_RAT Myosin regulatory light chain 2, ventricular/cardiac muscle isoform OS=Rattus norvegicus GN=Myl2 PE=1 SV=2 | 0.25 | 1 | 6 | 1 | 5 | 7 | 3 | 2 | 4.00 | 4.00 | 1.53 | 1.53 |
| sp\|Q62234\|MYOM1_MOUSE Myomesin-1 OS=Mus musculus GN=Myom1 PE=1 SV=2 | 0.26 | 0.4 | 6 | 3 | 0 | 2 | 1 | 1 | 3.00 | 1.33 | 1.73 | 0.33 |
| Cluster of sp\|Q3T147\|DX39B_BOVIN Spliceosome RNA helicase DDX39B OS=Bos taurus GN=DDX39B PE=2 SV=1 | 0.26 | 0.5 | 7 | 2 | 0 | 2 | 2 | 0 | 2.90 | 1.35 | 2.11 | 0.70 |
| sp\|P30050\|RL12_HUMAN 60S ribosomal protein L12 OS=Homo sapiens GN=RPL12 PE=1 SV=1 | 0.27 | 2.7 | 5 | 2 | 1 | 1 | 2 | 0 | 2.67 | 1.00 | 1.20 | 0.58 |
| Cluster of sp\|P19324\|SERPH_MOUSE Serpin H1 OS=Mus musculus GN=Serpinh1 PE=1 SV=3 | 0.27 | 1.2 | 33 | 12 | 0 | 17 | 11 | 9 | 15.06 | 12.31 | 9.81 | 2.50 |
| sp\|P09895\|RL5_RAT 60S ribosomal protein L5 OS=Rattus norvegicus GN=Rpl5 PE=1 SV=3 | 0.27 | 2.7 | 7 | 1 | 0 | 2 | 0 | 1 | 2.67 | 1.00 | 2.19 | 0.58 |
| sp\|P51174\|ACADL_MOUSE Long-chain specific acyl-CoA dehydrogenase, mitochondrial OS=Mus musculus GN=Acadl PE=1 SV=2 | 0.27 | 2.2 | 9 | 3 | 1 | 4 | 2 | 0 | 4.33 | 2.00 | 2.40 | 1.15 |
| sp\|Q14103\|HNRPD_HUMAN Heterogeneous nuclear ribonucleoprotein D0 OS=Homo sapiens GN=HNRNPD PE=1 SV=1 | 0.27 | 2.5 | 7 | 1 | 0 | 1 | 2 | 0 | 2.54 | 1.00 | 2.06 | 0.58 |
| sp\|Q63009\|ANM1_RAT Protein arginine N-methyltransferase 1 OS=Rattus norvegicus GN=Prmt1 PE=1 SV=1 | 0.27 | 2.7 | 6 | 1 | 1 | 2 | 0 | 1 | 2.67 | 1.00 | 1.67 | 0.58 |
| sp\|P35527\|K1C9_HUMAN Keratin, type I cytoskeletal 9 OS=Homo sapiens GN=KRT9 PE=1 SV=3 | 0.28 | 0.9 | 39 | 25 | 31 | 26 | 31 | 24 | 31.67 | 27.00 | 4.06 | 2.08 |
| sp\|A2ASQ1\|AGRIN_MOUSE Agrin OS=Mus musculus GN=Agrn PE=1 SV=1 | 0.28 | 1.1 | 3 | 2 | 4 | 5 | 4 | 1 | 2.97 | 3.30 | 0.58 | 1.19 |
| sp\|P48678\|LMNA_MOUSE Prelamin-A/C OS=Mus musculus GN=Lmna PE=1 SV=2 | 0.28 | 0.2 | 4 | 0 | 0 | 1 | 0 | 0 | 1.33 | 0.33 | 1.33 | 0.33 |
| sp\|P00639\|DNAS1_BOVIN Deoxyribonuclease-1 OS=Bos taurus GN=DNASE1 PE=1 SV=3 | 0.28 | 1.1 | 6 | 5 | 4 | 6 | 5 | 3 | 5.00 | 4.67 | 0.58 | 0.88 |
| Cluster of sp\|P07477\|TRY1_HUMAN Trypsin-1 OS=Homo sapiens GN=PRSS1 PE=1 SV=1 | 0.28 | 0.8 | 1 | 2 | 2 | 3 | 2 | 1 | 1.52 | 2.00 | 0.29 | 0.58 |
| sp\|P57776\|EF1D_MOUSE Elongation factor 1-delta OS=Mus musculus GN=Eef1d PE=1 SV=3 | 0.28 | 0.8 | 2 | 1 | 2 | 3 | 1 | 2 | 1.67 | 2.00 | 0.33 | 0.58 |
| Cluster of sp\|P69897\|TBB5_RAT Tubulin beta-5 chain OS=Rattus norvegicus GN=Tubb5 PE=1 SV=1 | 0.29 | 0.8 | 75 | 15 | 17 | 55 | 20 | 14 | 35.78 | 29.84 | 19.67 | 12.81 |
| sp\|P63039\|CH60_RAT 60 kDa heat shock protein, mitochondrial OS=Rattus norvegicus GN=Hspd1 PE=1 SV=1 | 0.29 | 0.6 | 30 | 2 | 1 | 18 | 1 | 2 | 11.00 | 7.00 | 9.50 | 5.51 |
| sp\|Q9CR62\|M2OM_MOUSE Mitochondrial 2-oxoglutarate/malate carrier protein OS=Mus musculus GN=Slc25a11 PE=1 SV=3 | 0.29 | 3 | 5 | 1 | 0 | 1 | 1 | 0 | 2.00 | 0.67 | 1.53 | 0.33 |
| sp\|Q9D0M3\|CY1_MOUSE Cytochrome c1, heme protein, mitochondrial OS=Mus musculus GN=Cyc1 PE=1 SV=1 | 0.29 | 0.8 | 4 | 0 | 0 | 2 | 2 | 1 | 1.33 | 1.67 | 1.33 | 0.33 |
| sp\|Q5RFJ1\|ILF2_PONAB Interleukin enhancer-binding factor 2 OS=Pongo abelii GN=ILF2 PE=2 SV=1 | 0.29 | 3 | 6 | 0 | 0 | 1 | 1 | 0 | 2.00 | 0.67 | 2.00 | 0.33 |
| sp\|Q6MG61\|CLIC1_RAT Chloride intracellular channel protein 1 OS=Rattus norvegicus GN=Clic1 PE=1 SV=1 | 0.29 | 0.8 | 3 | 1 | 0 | 4 | 0 | 1 | 1.33 | 1.67 | 0.88 | 1.20 |
| sp\|Q8JZQ9\|EIF3B_MOUSE Eukaryotic translation initiation factor 3 subunit B OS=Mus musculus GN=Eif3b PE=1 SV=1 | 0.3 | 0.5 | 8 | 3 | 2 | 2 | 1 | 4 | 4.33 | 2.33 | 1.86 | 0.88 |
| sp\|P10860\|DHE3_RAT Glutamate dehydrogenase 1, mitochondrial OS=Rattus norvegicus GN=Glud1 PE=1 SV=2 | 0.3 | 0.5 | 8 | 2 | 0 | 3 | 0 | 2 | 3.33 | 1.67 | 2.40 | 0.88 |
| sp\|Q07646\|MEST_MOUSE Mesoderm-specific transcript protein OS=Mus musculus GN=Mest PE=2 SV=1 | 0.31 | 2 | 9 | 3 | 2 | 4 | 1 | 2 | 4.67 | 2.33 | 2.19 | 0.88 |
| sp\|Q9QUP5\|HPLN1_MOUSE Hyaluronan and proteoglycan link protein 1 OS=Mus musculus GN=Hapln1 PE=1 SV=1 | 0.31 | 0.8 | 2 | 1 | 0 | 2 | 2 | 0 | 1.00 | 1.33 | 0.58 | 0.67 |
| sp\|P19783\|COX41_MOUSE Cytochrome c oxidase subunit 4 isoform 1, mitochondrial OS=Mus musculus GN=Cox4i1 PE=1 SV=2 | 0.31 | 0.8 | 3 | 0 | 0 | 2 | 1 | 1 | 1.00 | 1.33 | 1.00 | 0.33 |
| sp\|P19253\|RL13A_MOUSE 60S ribosomal protein L13a OS=Mus musculus GN=Rpl13a PE=1 SV=4 | 0.31 | 0.8 | 2 | 1 | 0 | 2 | 0 | 2 | 1.00 | 1.33 | 0.58 | 0.67 |
| Cluster of sp\|P04264\|K2C1_HUMAN Keratin, type II cytoskeletal 1 OS=Homo sapiens GN=KRT1 PE=1 SV=6 | 0.32 | 1.4 | 86 | 120 | 68 | 68 | 58 | 74 | 91.10 | 66.81 | 15.21 | 4.77 |
| Cluster of sp\|P02533\|K1C14_HUMAN Keratin, type I cytoskeletal 14 OS=Homo sapiens GN=KRT14 PE=1 SV=4 | 0.32 | 1.9 | 7 | 7 | 3 | 3 | 2 | 5 | 5.76 | 3.03 | 1.58 | 0.87 |
| sp\|P52480\|KPYM_MOUSE Pyruvate kinase PKM OS=Mus musculus GN=Pkm PE=1 SV=4 | 0.32 | 1.8 | 14 | 4 | 2 | 5 | 5 | 1 | 6.67 | 3.67 | 3.71 | 1.33 |
| sp\|P47961\|RS4_CRIGR 40S ribosomal protein S4 OS=Cricetulus griseus GN=RPS4 PE=2 SV=2 | 0.32 | 1.9 | 12 | 3 | 2 | 4 | 1 | 4 | 5.67 | 3.00 | 3.18 | 1.00 |
| sp\|P05712\|RAB2A_RAT Ras-related protein Rab-2A OS=Rattus norvegicus GN=Rab2a PE=2 SV=1 | 0.32 | 1 | 5 | 2 | 1 | 2 | 3 | 3 | 2.67 | 2.67 | 1.20 | 0.33 |
| sp\|Q8VHF5\|CISY_RAT Citrate synthase, mitochondrial OS=Rattus norvegicus GN=Cs PE=1 SV=1 | 0.32 | 1 | 4 | 1 | 3 | 4 | 3 | 1 | 2.67 | 2.67 | 0.88 | 0.88 |
| sp\|P47754\|CAZA2_MOUSE F-actin-capping protein subunit alpha-2 OS=Mus musculus GN=Capza2 PE=1 SV=3 | 0.32 | 2.4 | 3 | 0 | 0 | 1 | 0 | 0 | 1.13 | 0.47 | 1.13 | 0.47 |
| sp\|P35980\|RL18_MOUSE 60S ribosomal protein L18 OS=Mus musculus GN=Rpl18 PE=1 SV=3 | 0.32 | 2.2 | 3 | 3 | 3 | 1 | 1 | 2 | 3.00 | 1.33 | 0.00 | 0.33 |
| sp\|Q5E988\|RS5_BOVIN 40S ribosomal protein S5 OS=Bos taurus GN=RPS5 PE=2 SV=3 | 0.32 | 4 | 2 | 2 | 0 | 1 | 0 | 0 | 1.33 | 0.33 | 0.67 | 0.33 |
| sp\|Q29380\|VDAC3_PIG Voltage-dependent anion-selective channel protein 3 OS=Sus scrofa GN=VDAC3 PE=2 SV=2 | 0.32 | 4.4 | 4 | 0 | 0 | 0 | 1 | 0 | 1.46 | 0.33 | 1.46 | 0.33 |
| Cluster of sp\|Q61555\|FBN2_MOUSE Fibrillin-2 OS=Mus musculus GN=Fbn2 PE=1 SV=2 | 0.32 | 3.7 | 2 | 1 | 1 | 0 | 1 | 0 | 1.23 | 0.33 | 0.23 | 0.33 |
| Cluster of sp\|P70296\|PEBP1_MOUSE Phosphatidylethanolamine-binding protein 1 OS=Mus musculus GN=Pebp1 PE=1 SV=3 | 0.32 | 3.9 | 4 | 0 | 0 | 1 | 0 | 0 | 1.30 | 0.33 | 1.30 | 0.33 |
| sp\|Q9DCH4\|EIF3F_MOUSE Eukaryotic translation initiation factor 3 subunit F OS=Mus musculus GN=Eif3f PE=1 SV=2 | 0.32 | 4 | 4 | 0 | 0 | 1 | 0 | 0 | 1.33 | 0.33 | 1.33 | 0.33 |
| sp\|E9PAV3\|NACAM_HUMAN Nascent polypeptide-associated complex subunit alpha, muscle-specific form OS=Homo sapiens GN=NACA PE=1 SV=1 | 0.32 | 4 | 4 | 0 | 0 | 1 | 0 | 0 | 1.33 | 0.33 | 1.33 | 0.33 |
| sp\|Q9D8N0\|EF1G_MOUSE Elongation factor 1-gamma OS=Mus musculus GN=Eef1g PE=1 SV=3 | 0.33 | 1 | 9 | 2 | 1 | 6 | 3 | 3 | 4.00 | 4.00 | 2.52 | 1.00 |
| sp\|Q9CZS1\|AL1B1_MOUSE Aldehyde dehydrogenase X, mitochondrial OS=Mus musculus GN=Aldh1b1 PE=1 SV=1 | 0.33 | 1.2 | 4 | 0 | 1 | 4 | 0 | 2 | 1.67 | 2.00 | 1.20 | 1.15 |
| sp\|P63039\|CH60_RAT 60 kDa heat shock protein, mitochondrial OS=Rattus norvegicus GN=Hspd1 PE=1 SV=1 | 0.33 | 1.1 | 10 | 2 | 0 | 6 | 3 | 2 | 4.00 | 3.67 | 3.06 | 1.20 |
| sp\|O19049\|HNRPK_RABIT Heterogeneous nuclear ribonucleoprotein K OS=Oryctolagus cuniculus GN=HNRNPK PE=2 SV=1 | 0.33 | 1.1 | 7 | 4 | 1 | 5 | 5 | 1 | 4.00 | 3.67 | 1.73 | 1.33 |
| sp\|P42558\|RAN_CHICK GTP-binding nuclear protein Ran OS=Gallus gallus GN=RAN PE=2 SV=1 | 0.33 | 2 | 10 | 2 | 0 | 5 | 0 | 1 | 4.00 | 2.00 | 3.06 | 1.53 |
| sp\|Q02788\|CO6A2_MOUSE Collagen alpha-2(VI) chain OS=Mus musculus GN=Col6a2 PE=1 SV=3 | 0.34 | 1.2 | 2 | 0 | 2 | 0 | 2 | 3 | 1.33 | 1.67 | 0.67 | 0.88 |
| sp\|P40142\|TKT_MOUSE Transketolase OS=Mus musculus GN=Tkt PE=1 SV=1 | 0.34 | 0.5 | 8 | 0 | 0 | 3 | 1 | 0 | 2.67 | 1.33 | 2.67 | 0.88 |
| sp\|Q9D0I9\|SYRC_MOUSE Arginine--tRNA ligase, cytoplasmic OS=Mus musculus GN=Rars PE=1 SV=2 | 0.34 | 0.5 | 7 | 1 | 0 | 3 | 0 | 1 | 2.67 | 1.33 | 2.19 | 0.88 |
| sp\|P17225\|PTBP1_MOUSE Polypyrimidine tract-binding protein 1 OS=Mus musculus GN=Ptbp1 PE=1 SV=2 | 0.34 | 1.2 | 4 | 0 | 0 | 4 | 1 | 0 | 1.33 | 1.67 | 1.33 | 1.20 |
| sp\|P14115\|RL27A_MOUSE 60S ribosomal protein L27a OS=Mus musculus GN=Rpl27a PE=1 SV=5 | 0.34 | 1 | 3 | 2 | 2 | 4 | 1 | 2 | 2.33 | 2.33 | 0.33 | 0.88 |
| sp\|O54724\|PTRF_MOUSE Polymerase I and transcript release factor OS=Mus musculus GN=Ptrf PE=1 SV=1 | 0.34 | 0.7 | 2 | 0 | 0 | 3 | 0 | 0 | 0.67 | 1.00 | 0.67 | 1.00 |
| sp\|Q9D6J6\|NDUV2_MOUSE NADH dehydrogenase [ubiquinone] flavoprotein 2, mitochondrial OS=Mus musculus GN=Ndufv2 PE=1 SV=2 | 0.34 | 0.7 | 2 | 0 | 0 | 3 | 0 | 0 | 0.67 | 1.00 | 0.67 | 1.00 |
| sp\|Q9R0Y5\|KAD1_MOUSE Adenylate kinase isoenzyme 1 OS=Mus musculus GN=Ak1 PE=1 SV=1 | 0.34 | 0.7 | 2 | 0 | 0 | 3 | 0 | 0 | 0.67 | 1.00 | 0.67 | 1.00 |
| sp\|O09061\|PSB1_MOUSE Proteasome subunit beta type-1 OS=Mus musculus GN=Psmb1 PE=1 SV=1 | 0.34 | 0.7 | 2 | 0 | 0 | 3 | 0 | 0 | 0.67 | 1.00 | 0.67 | 1.00 |
| sp\|Q5XFW8\|SEC13_RAT Protein SEC13 homolog OS=Rattus norvegicus GN=Sec13 PE=1 SV=1 | 0.34 | 0.7 | 2 | 0 | 0 | 2 | 0 | 1 | 0.67 | 1.00 | 0.67 | 0.58 |
| sp\|P62899\|RL31_HUMAN 60S ribosomal protein L31 OS=Homo sapiens GN=RPL31 PE=1 SV=1 | 0.34 | 0.7 | 1 | 0 | 1 | 1 | 1 | 1 | 0.67 | 1.00 | 0.33 | 0.00 |
| sp\|O70435\|PSA3_MOUSE Proteasome subunit alpha type-3 OS=Mus musculus GN=Psma3 PE=1 SV=3 | 0.34 | 0.7 | 2 | 0 | 0 | 1 | 2 | 0 | 0.67 | 1.00 | 0.67 | 0.58 |
| sp\|O88322\|NID2_MOUSE Nidogen-2 OS=Mus musculus GN=Nid2 PE=1 SV=2 | 0.35 | 1 | 11 | 6 | 6 | 11 | 6 | 5 | 7.21 | 6.87 | 1.67 | 1.86 |
| sp\|P35615\|ERF1_XENLA Eukaryotic peptide chain release factor subunit 1 OS=Xenopus laevis GN=etf1 PE=2 SV=1 | 0.35 | 0.4 | 4 | 1 | 0 | 2 | 0 | 0 | 1.67 | 0.67 | 1.20 | 0.67 |
| Cluster of sp\|Q03265\|ATPA_MOUSE ATP synthase subunit alpha, mitochondrial OS=Mus musculus GN=Atp5a1 PE=1 SV=1 | 0.35 | 1.3 | 57 | 28 | 17 | 33 | 19 | 24 | 33.79 | 25.24 | 12.09 | 4.03 |
| sp\|P62907\|RL10A_RAT 60S ribosomal protein L10a OS=Rattus norvegicus GN=Rpl10a PE=1 SV=2 | 0.35 | 2.3 | 6 | 1 | 0 | 1 | 1 | 1 | 2.33 | 1.00 | 1.86 | 0.00 |
| sp\|P30668\|TBB_SCHCO Tubulin beta chain OS=Schizophyllum commune GN=TUB-2 PE=3 SV=1 | 0.35 | 2.4 | 5 | 2 | 0 | 3 | 0 | 0 | 2.40 | 1.00 | 1.43 | 1.00 |
| sp\|Q99LC5\|ETFA_MOUSE Electron transfer flavoprotein subunit alpha, mitochondrial OS=Mus musculus GN=Etfa PE=1 SV=2 | 0.35 | 2.3 | 7 | 0 | 0 | 3 | 0 | 0 | 2.33 | 1.00 | 2.33 | 1.00 |
| sp\|P19123\|TNNC1_MOUSE Troponin C, slow skeletal and cardiac muscles OS=Mus musculus GN=Tnnc1 PE=1 SV=1 | 0.35 | 2.3 | 5 | 1 | 1 | 2 | 0 | 1 | 2.33 | 1.00 | 1.33 | 0.58 |
| Cluster of sp\|P56480\|ATPB_MOUSE ATP synthase subunit beta, mitochondrial OS=Mus musculus GN=Atp5b PE=1 SV=2 | 0.36 | 1.3 | 47 | 13 | 12 | 21 | 14 | 19 | 23.76 | 17.76 | 11.44 | 2.06 |
| sp\|P35232\|PHB_HUMAN Prohibitin OS=Homo sapiens GN=PHB PE=1 SV=1 | 0.37 | 1.2 | 11 | 5 | 6 | 6 | 6 | 6 | 7.33 | 6.00 | 1.86 | 0.00 |
| sp\|O18789\|RS2_BOVIN 40S ribosomal protein S2 OS=Bos taurus GN=RPS2 PE=2 SV=2 | 0.37 | 1.9 | 7 | 4 | 2 | 2 | 2 | 3 | 4.33 | 2.33 | 1.45 | 0.33 |
| sp\|Q8VHX6\|FLNC_MOUSE Filamin-C OS=Mus musculus GN=Flnc PE=1 SV=3 | 0.38 | 0.8 | 92 | 30 | 22 | 53 | 28 | 35 | 48.08 | 38.58 | 22.32 | 7.36 |
| sp\|P27773\|PDIA3_MOUSE Protein disulfide-isomerase A3 OS=Mus musculus GN=Pdia3 PE=1 SV=2 | 0.38 | 0.9 | 12 | 1 | 1 | 10 | 1 | 2 | 4.67 | 4.33 | 3.67 | 2.85 |
| sp\|Q02SZ7\|LYSC_PSEAB Lysyl endopeptidase OS=Pseudomonas aeruginosa (strain UCBPP-PA14) GN=prpL PE=1 SV=1 | 0.38 | 1.5 | 0 | 1 | 1 | 0 | 3 | 0 | 0.67 | 1.00 | 0.33 | 1.00 |
| Cluster of sp\|P04692\|TPM1_RAT Tropomyosin alpha-1 chain OS=Rattus norvegicus GN=Tpm1 PE=1 SV=3 | 0.38 | 1.2 | 14 | 0 | 0 | 7 | 4 | 1 | 4.56 | 3.96 | 4.56 | 1.73 |
| Cluster of sp\|P19378\|HSP7C_CRIGR Heat shock cognate 71 kDa protein OS=Cricetulus griseus GN=HSPA8 PE=2 SV=1 | 0.39 | 0.7 | 48 | 15 | 17 | 20 | 22 | 16 | 26.81 | 19.24 | 10.84 | 1.77 |
| sp\|Q922R8\|PDIA6_MOUSE Protein disulfide-isomerase A6 OS=Mus musculus GN=Pdia6 PE=1 SV=3 | 0.39 | 1 | 8 | 0 | 0 | 8 | 0 | 0 | 2.67 | 2.67 | 2.67 | 2.67 |
| sp\|Q922Q8\|LRC59_MOUSE Leucine-rich repeat-containing protein 59 OS=Mus musculus GN=Lrrc59 PE=1 SV=1 | 0.39 | 2.5 | 4 | 1 | 0 | 2 | 0 | 0 | 1.67 | 0.67 | 1.20 | 0.67 |
| sp\|B5DF91\|ELAV1_RAT ELAV-like protein 1 OS=Rattus norvegicus GN=Elavl1 PE=1 SV=1 | 0.39 | 2.5 | 5 | 0 | 0 | 2 | 0 | 0 | 1.67 | 0.67 | 1.67 | 0.67 |
| Cluster of sp\|P20152\|VIME_MOUSE Vimentin OS=Mus musculus GN=Vim PE=1 SV=3 | 0.4 | 0.8 | 34 | 13 | 11 | 28 | 9 | 9 | 19.13 | 15.48 | 7.49 | 6.50 |
| sp\|P05202\|AATM_MOUSE Aspartate aminotransferase, mitochondrial OS=Mus musculus GN=Got2 PE=1 SV=1 | 0.4 | 1.6 | 22 | 6 | 4 | 9 | 5 | 6 | 10.67 | 6.67 | 5.70 | 1.20 |
| sp\|P63330\|PP2AA_MOUSE Serine/threonine-protein phosphatase 2A catalytic subunit alpha isoform OS=Mus musculus GN=Ppp2ca PE=1 SV=1 | 0.4 | 2 | 7 | 1 | 0 | 2 | 2 | 0 | 2.67 | 1.33 | 2.19 | 0.67 |
| sp\|Q9DCT8\|CRIP2_MOUSE Cysteine-rich protein 2 OS=Mus musculus GN=Crip2 PE=1 SV=1 | 0.4 | 2 | 4 | 2 | 2 | 2 | 0 | 2 | 2.67 | 1.33 | 0.67 | 0.67 |
| sp\|P51410\|RL9_MOUSE 60S ribosomal protein L9 OS=Mus musculus GN=Rpl9 PE=2 SV=2 | 0.4 | 2 | 7 | 1 | 0 | 3 | 1 | 0 | 2.67 | 1.33 | 2.19 | 0.88 |
| Cluster of sp\|O70468\|MYPC3_MOUSE Myosin-binding protein C, cardiac-type OS=Mus musculus GN=Mybpc3 PE=1 SV=1 | 0.41 | 0.8 | 75 | 48 | 21 | 53 | 27 | 35 | 47.86 | 38.36 | 15.59 | 7.49 |
| sp\|P80315\|TCPD_MOUSE T-complex protein 1 subunit delta OS=Mus musculus GN=Cct4 PE=1 SV=3 | 0.41 | 1 | 6 | 1 | 0 | 5 | 1 | 1 | 2.33 | 2.33 | 1.86 | 1.33 |
| Cluster of sp\|P06745\|G6PI_MOUSE Glucose-6-phosphate isomerase OS=Mus musculus GN=Gpi PE=1 SV=4 | 0.41 | 1 | 7 | 0 | 0 | 7 | 0 | 0 | 2.33 | 2.33 | 2.33 | 2.33 |
| sp\|P06151\|LDHA_MOUSE L-lactate dehydrogenase A chain OS=Mus musculus GN=Ldha PE=1 SV=3 | 0.41 | 1.7 | 13 | 2 | 2 | 6 | 2 | 2 | 5.67 | 3.33 | 3.67 | 1.33 |
| sp\|P47911\|RL6_MOUSE 60S ribosomal protein L6 OS=Mus musculus GN=Rpl6 PE=1 SV=3 | 0.41 | 1.7 | 10 | 3 | 1 | 3 | 3 | 2 | 4.67 | 2.67 | 2.73 | 0.33 |
| sp\|P49432\|ODPB_RAT Pyruvate dehydrogenase E1 component subunit beta, mitochondrial OS=Rattus norvegicus GN=Pdhb PE=1 SV=2 | 0.41 | 1.7 | 10 | 4 | 0 | 4 | 1 | 3 | 4.67 | 2.67 | 2.91 | 0.88 |
| sp\|P14851\|PPIA_CRIGR Peptidyl-prolyl cis-trans isomerase A OS=Cricetulus griseus GN=PPIA PE=2 SV=2 | 0.41 | 1.7 | 11 | 1 | 2 | 7 | 1 | 0 | 4.67 | 2.67 | 3.18 | 2.19 |
| sp\|B2GV06\|SCOT1_RAT Succinyl-CoA:3-ketoacid coenzyme A transferase 1, mitochondrial OS=Rattus norvegicus GN=Oxct1 PE=1 SV=1 | 0.42 | 0.3 | 2 | 1 | 0 | 1 | 0 | 0 | 1.00 | 0.33 | 0.58 | 0.33 |
| sp\|Q99JY0\|ECHB_MOUSE Trifunctional enzyme subunit beta, mitochondrial OS=Mus musculus GN=Hadhb PE=1 SV=1 | 0.42 | 0.3 | 3 | 0 | 0 | 0 | 0 | 1 | 1.00 | 0.33 | 1.00 | 0.33 |
| sp\|Q9EQP2\|EHD4_MOUSE EH domain-containing protein 4 OS=Mus musculus GN=Ehd4 PE=1 SV=1 | 0.42 | 0.3 | 3 | 0 | 0 | 1 | 0 | 0 | 1.00 | 0.33 | 1.00 | 0.33 |
| sp\|P36604\|GRP78_SCHPO 78 kDa glucose-regulated protein homolog OS=Schizosaccharomyces pombe (strain 972 / ATCC 24843) GN=bip1 PE=3 SV=2 | 0.42 | 0.3 | 0 | 2 | 1 | 0 | 1 | 0 | 1.09 | 0.36 | 0.63 | 0.36 |
| sp\|P35564\|CALX_MOUSE Calnexin OS=Mus musculus GN=Canx PE=1 SV=1 | 0.43 | 1 | 5 | 1 | 0 | 5 | 1 | 0 | 2.00 | 2.00 | 1.53 | 1.53 |
| sp\|P09103\|PDIA1_MOUSE Protein disulfide-isomerase OS=Mus musculus GN=P4hb PE=1 SV=2 | 0.43 | 1 | 6 | 0 | 0 | 4 | 2 | 0 | 2.00 | 2.00 | 2.00 | 1.15 |
| sp\|P48036\|ANXA5_MOUSE Annexin A5 OS=Mus musculus GN=Anxa5 PE=1 SV=1 | 0.43 | 1.1 | 8 | 0 | 0 | 7 | 0 | 0 | 2.67 | 2.33 | 2.67 | 2.33 |
| sp\|P09542\|MYL3_MOUSE Myosin light chain 3 OS=Mus musculus GN=Myl3 PE=1 SV=4 | 0.43 | 1 | 6 | 2 | 0 | 7 | 0 | 1 | 2.56 | 2.56 | 1.70 | 2.08 |
| Cluster of sp\|P62262\|1433E_SHEEP 14-3-3 protein epsilon OS=Ovis aries GN=YWHAE PE=1 SV=1 | 0.44 | 1.7 | 10 | 1 | 1 | 4 | 2 | 1 | 3.94 | 2.33 | 2.94 | 0.88 |
| sp\|P14148\|RL7_MOUSE 60S ribosomal protein L7 OS=Mus musculus GN=Rpl7 PE=1 SV=2 | 0.44 | 1.7 | 7 | 3 | 2 | 1 | 3 | 3 | 4.00 | 2.33 | 1.53 | 0.67 |
| sp\|Q61425\|HCDH_MOUSE Hydroxyacyl-coenzyme A dehydrogenase, mitochondrial OS=Mus musculus GN=Hadh PE=1 SV=2 | 0.44 | 1 | 4 | 0 | 0 | 2 | 1 | 1 | 1.33 | 1.33 | 1.33 | 0.33 |
| sp\|P17074\|RS19_RAT 40S ribosomal protein S19 OS=Rattus norvegicus GN=Rps19 PE=2 SV=3 | 0.44 | 1 | 4 | 0 | 0 | 1 | 2 | 1 | 1.33 | 1.33 | 1.33 | 0.33 |
| sp\|P62852\|RS25_MOUSE 40S ribosomal protein S25 OS=Mus musculus GN=Rps25 PE=1 SV=1 | 0.44 | 1 | 3 | 0 | 1 | 2 | 1 | 1 | 1.33 | 1.33 | 0.88 | 0.33 |
| sp\|P07477\|TRY1_HUMAN Trypsin-1 OS=Homo sapiens GN=PRSS1 PE=1 SV=1 | 0.45 | 0.9 | 4 | 3 | 3 | 3 | 4 | 2 | 3.33 | 3.00 | 0.33 | 0.58 |
| sp\|Q9QVP4\|MLRA_MOUSE Myosin regulatory light chain 2, atrial isoform OS=Mus musculus GN=Myl7 PE=1 SV=1 | 0.45 | 1.3 | 11 | 3 | 4 | 3 | 5 | 6 | 6.00 | 4.67 | 2.52 | 0.88 |
| sp\|P41123\|RL13_RAT 60S ribosomal protein L13 OS=Rattus norvegicus GN=Rpl13 PE=1 SV=2 | 0.45 | 2 | 3 | 2 | 1 | 2 | 0 | 1 | 2.00 | 1.00 | 0.58 | 0.58 |
| sp\|P62250\|RS16_RAT 40S ribosomal protein S16 OS=Rattus norvegicus GN=Rps16 PE=1 SV=2 | 0.45 | 1.8 | 6 | 2 | 1 | 3 | 1 | 1 | 3.00 | 1.67 | 1.53 | 0.67 |
| sp\|P09405\|NUCL_MOUSE Nucleolin OS=Mus musculus GN=Ncl PE=1 SV=2 | 0.45 | 2 | 6 | 0 | 0 | 1 | 2 | 0 | 2.00 | 1.00 | 2.00 | 0.58 |
| sp\|P50247\|SAHH_MOUSE Adenosylhomocysteinase OS=Mus musculus GN=Ahcy PE=1 SV=3 | 0.45 | 3 | 3 | 0 | 0 | 1 | 0 | 0 | 1.00 | 0.33 | 1.00 | 0.33 |
| sp\|P27773\|PDIA3_MOUSE Protein disulfide-isomerase A3 OS=Mus musculus GN=Pdia3 PE=1 SV=2 | 0.45 | 3 | 3 | 0 | 0 | 1 | 0 | 0 | 1.00 | 0.33 | 1.00 | 0.33 |
| sp\|P59998\|ARPC4_HUMAN Actin-related protein 2/3 complex subunit 4 OS=Homo sapiens GN=ARPC4 PE=1 SV=3 | 0.45 | 2 | 2 | 2 | 2 | 1 | 1 | 1 | 2.00 | 1.00 | 0.00 | 0.00 |
| sp\|Q99020\|ROAA_MOUSE Heterogeneous nuclear ribonucleoprotein A/B OS=Mus musculus GN=Hnrnpab PE=1 SV=1 | 0.45 | 3 | 3 | 0 | 0 | 1 | 0 | 0 | 1.00 | 0.33 | 1.00 | 0.33 |
| sp\|O43396\|TXNL1_HUMAN Thioredoxin-like protein 1 OS=Homo sapiens GN=TXNL1 PE=1 SV=3 | 0.45 | 3 | 3 | 0 | 0 | 1 | 0 | 0 | 1.00 | 0.33 | 1.00 | 0.33 |
| sp\|Q9WUM5\|SUCA_MOUSE Succinate--CoA ligase [ADP/GDP-forming] subunit alpha, mitochondrial OS=Mus musculus GN=Suclg1 PE=1 SV=4 | 0.45 | 3 | 2 | 1 | 0 | 1 | 0 | 0 | 1.00 | 0.33 | 0.58 | 0.33 |
| sp\|P20108\|PRDX3_MOUSE Thioredoxin-dependent peroxide reductase, mitochondrial OS=Mus musculus GN=Prdx3 PE=1 SV=1 | 0.45 | 3 | 3 | 0 | 0 | 1 | 0 | 0 | 1.00 | 0.33 | 1.00 | 0.33 |
| sp\|P47955\|RLA1_MOUSE 60S acidic ribosomal protein P1 OS=Mus musculus GN=Rplp1 PE=1 SV=1 | 0.45 | 3 | 3 | 0 | 0 | 1 | 0 | 0 | 1.00 | 0.33 | 1.00 | 0.33 |
| sp\|P04897\|GNAI2_RAT Guanine nucleotide-binding protein G(i) subunit alpha-2 OS=Rattus norvegicus GN=Gnai2 PE=1 SV=3 | 0.45 | 3 | 3 | 0 | 0 | 1 | 0 | 0 | 1.00 | 0.33 | 1.00 | 0.33 |
| sp\|A0PFK7\|CAPZB_PIG F-actin-capping protein subunit beta OS=Sus scrofa GN=CAPZB PE=2 SV=1 | 0.45 | 3 | 3 | 0 | 0 | 1 | 0 | 0 | 1.00 | 0.33 | 1.00 | 0.33 |
| sp\|P80314\|TCPB_MOUSE T-complex protein 1 subunit beta OS=Mus musculus GN=Cct2 PE=1 SV=4 | 0.45 | 3 | 2 | 0 | 1 | 0 | 1 | 0 | 1.00 | 0.33 | 0.58 | 0.33 |
| sp\|P50461\|CSRP3_HUMAN Cysteine and glycine-rich protein 3 OS=Homo sapiens GN=CSRP3 PE=1 SV=1 | 0.45 | 3 | 2 | 1 | 0 | 1 | 0 | 0 | 1.00 | 0.33 | 0.58 | 0.33 |
| sp\|Q75Q40\|TOM40_RAT Mitochondrial import receptor subunit TOM40 homolog OS=Rattus norvegicus GN=Tomm40 PE=1 SV=1 | 0.45 | 3 | 2 | 1 | 0 | 1 | 0 | 0 | 1.00 | 0.33 | 0.58 | 0.33 |
| sp\|P46792\|RS22_AGABI 40S ribosomal protein S22 OS=Agaricus bisporus GN=rps22 PE=2 SV=1 | 0.45 | 3 | 3 | 0 | 0 | 1 | 0 | 0 | 1.00 | 0.33 | 1.00 | 0.33 |
| sp\|P62909\|RS3_RAT 40S ribosomal protein S3 OS=Rattus norvegicus GN=Rps3 PE=1 SV=1 | 0.46 | 1.4 | 28 | 10 | 4 | 12 | 8 | 11 | 14.00 | 10.33 | 7.21 | 1.20 |
| Cluster of sp\|P11499\|HS90B_MOUSE Heat shock protein HSP 90-beta OS=Mus musculus GN=Hsp90ab1 PE=1 SV=3 | 0.46 | 1.5 | 48 | 7 | 0 | 25 | 12 | 0 | 18.31 | 12.48 | 14.95 | 7.26 |
| sp\|P62754\|RS6_MOUSE 40S ribosomal protein S6 OS=Mus musculus GN=Rps6 PE=1 SV=1 | 0.46 | 1.2 | 4 | 2 | 1 | 1 | 3 | 2 | 2.33 | 2.00 | 0.88 | 0.58 |
| Cluster of sp\|P68363\|TBA1B_HUMAN Tubulin alpha-1B chain OS=Homo sapiens GN=TUBA1B PE=1 SV=1 | 0.47 | 0.8 | 77 | 24 | 21 | 45 | 29 | 23 | 40.83 | 31.99 | 18.13 | 6.52 |
| sp\|Q07113\|MPRI_MOUSE Cation-independent mannose-6-phosphate receptor OS=Mus musculus GN=Igf2r PE=1 SV=1 | 0.47 | 0.7 | 38 | 13 | 8 | 21 | 9 | 14 | 19.67 | 14.67 | 9.28 | 3.48 |
| sp\|P42932\|TCPQ_MOUSE T-complex protein 1 subunit theta OS=Mus musculus GN=Cct8 PE=1 SV=3 | 0.47 | 0.6 | 8 | 0 | 0 | 4 | 1 | 0 | 2.67 | 1.67 | 2.67 | 1.20 |
| sp\|Q3SZ20\|GLYM_BOVIN Serine hydroxymethyltransferase, mitochondrial OS=Bos taurus GN=SHMT2 PE=2 SV=1 | 0.47 | 0.5 | 4 | 0 | 0 | 2 | 0 | 0 | 1.27 | 0.67 | 1.27 | 0.67 |
| Cluster of sp\|Q4R304\|RBBP7_MACFA Histone-binding protein RBBP7 OS=Macaca fascicularis GN=RBBP7 PE=2 SV=1 | 0.47 | 0.6 | 4 | 0 | 0 | 2 | 0 | 0 | 1.17 | 0.67 | 1.17 | 0.67 |
| sp\|Q5E9J1\|HNRPF_BOVIN Heterogeneous nuclear ribonucleoprotein F OS=Bos taurus GN=HNRNPF PE=2 SV=3 | 0.47 | 0.4 | 4 | 0 | 0 | 0 | 0 | 2 | 1.36 | 0.51 | 1.36 | 0.51 |
| sp\|P68040\|RACK1_MOUSE Receptor of activated protein C kinase 1 OS=Mus musculus GN=Rack1 PE=1 SV=3 | 0.47 | 1.5 | 26 | 8 | 5 | 12 | 7 | 7 | 13.00 | 8.67 | 6.56 | 1.67 |
| sp\|P08122\|CO4A2_MOUSE Collagen alpha-2(IV) chain OS=Mus musculus GN=Col4a2 PE=1 SV=4 | 0.48 | 0.9 | 3 | 2 | 4 | 5 | 2 | 1 | 3.00 | 2.67 | 0.58 | 1.20 |
| sp\|P14869\|RLA0_MOUSE 60S acidic ribosomal protein P0 OS=Mus musculus GN=Rplp0 PE=1 SV=3 | 0.48 | 1.5 | 14 | 7 | 2 | 7 | 5 | 3 | 7.67 | 5.00 | 3.48 | 1.15 |
| Cluster of sp\|P61107\|RAB14_RAT Ras-related protein Rab-14 OS=Rattus norvegicus GN=Rab14 PE=1 SV=3 | 0.48 | 1.3 | 7 | 2 | 1 | 3 | 3 | 2 | 3.33 | 2.51 | 1.82 | 0.49 |
| Cluster of sp\|O46674\|AT2A2_CANLF Sarcoplasmic/endoplasmic reticulum calcium ATPase 2 OS=Canis lupus familiaris GN=ATP2A2 PE=2 SV=1 | 0.48 | 0.7 | 3 | 0 | 0 | 3 | 0 | 1 | 0.85 | 1.19 | 0.85 | 0.74 |
| sp\|P17220\|PSA2_RAT Proteasome subunit alpha type-2 OS=Rattus norvegicus GN=Psma2 PE=1 SV=3 | 0.48 | 1 | 3 | 0 | 0 | 3 | 0 | 0 | 1.00 | 1.00 | 1.00 | 1.00 |
| sp\|Q6DHE8\|RHOAD_DANRE Rho-related GTP-binding protein RhoA-D OS=Danio rerio GN=rhoad PE=1 SV=1 | 0.48 | 1 | 3 | 0 | 0 | 2 | 1 | 0 | 1.00 | 1.00 | 1.00 | 0.58 |
| sp\|P47772\|RS13_ICTPU 40S ribosomal protein S13 OS=Ictalurus punctatus GN=rps13 PE=2 SV=3 | 0.48 | 1 | 3 | 0 | 0 | 2 | 1 | 0 | 1.00 | 1.00 | 1.00 | 0.58 |
| sp\|P10493\|NID1_MOUSE Nidogen-1 OS=Mus musculus GN=Nid1 PE=1 SV=2 | 0.49 | 0.8 | 5 | 2 | 5 | 3 | 3 | 3 | 4.46 | 3.46 | 1.00 | 0.00 |
| Cluster of sp\|P56480\|ATPB_MOUSE ATP synthase subunit beta, mitochondrial OS=Mus musculus GN=Atp5b PE=1 SV=2 | 0.49 | 0.8 | 63 | 21 | 15 | 45 | 14 | 17 | 32.75 | 25.12 | 15.03 | 9.79 |
| sp\|P21981\|TGM2_MOUSE Protein-glutamine gamma-glutamyltransferase 2 OS=Mus musculus GN=Tgm2 PE=1 SV=4 | 0.49 | 0.7 | 13 | 5 | 8 | 9 | 5 | 5 | 8.67 | 6.33 | 2.33 | 1.33 |
| Cluster of sp\|P09541\|MYL4_MOUSE Myosin light chain 4 OS=Mus musculus GN=Myl4 PE=1 SV=3 | 0.49 | 1.7 | 6 | 2 | 2 | 4 | 1 | 1 | 3.27 | 1.93 | 1.37 | 1.04 |
| sp\|Q62261\|SPTB2_MOUSE Spectrin beta chain, non-erythrocytic 1 OS=Mus musculus GN=Sptbn1 PE=1 SV=2 | 0.5 | 0.8 | 41 | 15 | 14 | 27 | 13 | 13 | 23.33 | 17.67 | 8.84 | 4.67 |
| sp\|P48962\|ADT1_MOUSE ADP/ATP translocase 1 OS=Mus musculus GN=Slc25a4 PE=1 SV=4 | 0.5 | 0.8 | 23 | 10 | 7 | 11 | 8 | 12 | 13.06 | 10.21 | 4.99 | 1.25 |
| sp\|P08249\|MDHM_MOUSE Malate dehydrogenase, mitochondrial OS=Mus musculus GN=Mdh2 PE=1 SV=3 | 0.5 | 1.3 | 15 | 1 | 0 | 11 | 1 | 0 | 5.33 | 4.00 | 4.84 | 3.51 |
| sp\|P62083\|RS7_RAT 40S ribosomal protein S7 OS=Rattus norvegicus GN=Rps7 PE=1 SV=1 | 0.5 | 1.7 | 3 | 3 | 1 | 3 | 1 | 0 | 2.33 | 1.33 | 0.67 | 0.88 |
| sp\|P04444\|HBBZ_MOUSE Hemoglobin subunit beta-H1 OS=Mus musculus GN=Hbb-bh1 PE=2 SV=3 | 0.5 | 1.2 | 6 | 0 | 0 | 3 | 2 | 0 | 1.94 | 1.66 | 1.94 | 0.88 |
| sp\|P07356\|ANXA2_MOUSE Annexin A2 OS=Mus musculus GN=Anxa2 PE=1 SV=2 | 0.51 | 1.6 | 11 | 1 | 2 | 6 | 2 | 1 | 4.67 | 3.00 | 3.18 | 1.53 |
| sp\|O88989\|MDHC_RAT Malate dehydrogenase, cytoplasmic OS=Rattus norvegicus GN=Mdh1 PE=1 SV=3 | 0.51 | 1.3 | 7 | 2 | 0 | 6 | 1 | 0 | 3.00 | 2.33 | 2.08 | 1.86 |
| Cluster of sp\|Q9QXS1\|PLEC_MOUSE Plectin OS=Mus musculus GN=Plec PE=1 SV=3 | 0.52 | 0.8 | 13 | 4 | 4 | 10 | 3 | 3 | 6.79 | 5.18 | 2.92 | 2.27 |
| Cluster of sp\|P48962\|ADT1_MOUSE ADP/ATP translocase 1 OS=Mus musculus GN=Slc25a4 PE=1 SV=4 | 0.52 | 1.4 | 49 | 20 | 14 | 19 | 15 | 24 | 27.41 | 19.27 | 10.72 | 2.60 |
| sp\|Q9CZ13\|QCR1_MOUSE Cytochrome b-c1 complex subunit 1, mitochondrial OS=Mus musculus GN=Uqcrc1 PE=1 SV=2 | 0.52 | 1.6 | 11 | 0 | 0 | 3 | 1 | 3 | 3.67 | 2.33 | 3.67 | 0.67 |
| sp\|P25698\|EF1A_SOYBN Elongation factor 1-alpha OS=Glycine max GN=TEFS1 PE=3 SV=2 | 0.52 | 2 | 3 | 1 | 0 | 1 | 0 | 1 | 1.33 | 0.67 | 0.88 | 0.33 |
| Cluster of sp\|P05132\|KAPCA_MOUSE cAMP-dependent protein kinase catalytic subunit alpha OS=Mus musculus GN=Prkaca PE=1 SV=3 | 0.52 | 2.3 | 4 | 0 | 0 | 2 | 0 | 0 | 1.33 | 0.58 | 1.33 | 0.58 |
| sp\|Q99PT1\|GDIR1_MOUSE Rho GDP-dissociation inhibitor 1 OS=Mus musculus GN=Arhgdia PE=1 SV=3 | 0.52 | 2 | 4 | 0 | 0 | 2 | 0 | 0 | 1.33 | 0.67 | 1.33 | 0.67 |
| sp\|P62831\|RL23_PIG 60S ribosomal protein L23 OS=Sus scrofa GN=RPL23 PE=1 SV=1 | 0.52 | 2 | 4 | 0 | 0 | 1 | 0 | 1 | 1.33 | 0.67 | 1.33 | 0.33 |
| sp\|P47753\|CAZA1_MOUSE F-actin-capping protein subunit alpha-1 OS=Mus musculus GN=Capza1 PE=1 SV=4 | 0.52 | 2.9 | 5 | 0 | 0 | 2 | 0 | 0 | 1.53 | 0.53 | 1.53 | 0.53 |
| sp\|P39061\|COIA1_MOUSE Collagen alpha-1(XVIII) chain OS=Mus musculus GN=Col18a1 PE=1 SV=4 | 0.53 | 1 | 3 | 0 | 0 | 2 | 1 | 0 | 1.00 | 1.00 | 1.00 | 0.58 |
| sp\|O19049\|HNRPK_RABIT Heterogeneous nuclear ribonucleoprotein K OS=Oryctolagus cuniculus GN=HNRNPK PE=2 SV=1 | 0.53 | 0.8 | 6 | 3 | 2 | 3 | 3 | 3 | 3.67 | 3.00 | 1.20 | 0.00 |
| sp\|Q99K41\|EMIL1_MOUSE EMILIN-1 OS=Mus musculus GN=Emilin1 PE=1 SV=1 | 0.53 | 0.9 | 3 | 1 | 3 | 2 | 2 | 2 | 2.33 | 2.00 | 0.67 | 0.00 |
| sp\|P85845\|FSCN1_RAT Fascin OS=Rattus norvegicus GN=Fscn1 PE=1 SV=2 | 0.53 | 1 | 3 | 0 | 0 | 3 | 0 | 0 | 1.00 | 1.00 | 1.00 | 1.00 |
| sp\|P24369\|PPIB_MOUSE Peptidyl-prolyl cis-trans isomerase B OS=Mus musculus GN=Ppib PE=1 SV=2 | 0.54 | 1.6 | 5 | 1 | 2 | 2 | 1 | 2 | 2.65 | 1.64 | 1.21 | 0.32 |
| sp\|P21796\|VDAC1_HUMAN Voltage-dependent anion-selective channel protein 1 OS=Homo sapiens GN=VDAC1 PE=1 SV=2 | 0.54 | 1.5 | 8 | 0 | 0 | 5 | 0 | 0 | 2.54 | 1.66 | 2.54 | 1.66 |
| sp\|Q9DCW4\|ETFB_MOUSE Electron transfer flavoprotein subunit beta OS=Mus musculus GN=Etfb PE=1 SV=3 | 0.56 | 1.7 | 5 | 0 | 0 | 3 | 0 | 0 | 1.67 | 1.00 | 1.67 | 1.00 |
| sp\|P18760\|COF1_MOUSE Cofilin-1 OS=Mus musculus GN=Cfl1 PE=1 SV=3 | 0.56 | 1.7 | 5 | 0 | 0 | 3 | 0 | 0 | 1.67 | 1.00 | 1.67 | 1.00 |
| sp\|Q60587\|ECHB_RAT Trifunctional enzyme subunit beta, mitochondrial OS=Rattus norvegicus GN=Hadhb PE=1 SV=1 | 0.56 | 1.7 | 4 | 1 | 0 | 1 | 1 | 1 | 1.67 | 1.00 | 1.20 | 0.00 |
| Cluster of sp\|Q9JI91\|ACTN2_MOUSE Alpha-actinin-2 OS=Mus musculus GN=Actn2 PE=1 SV=2 | 0.57 | 1.5 | 15 | 1 | 1 | 7 | 2 | 3 | 5.69 | 3.88 | 4.69 | 1.46 |
| sp\|Q5XIH7\|PHB2_RAT Prohibitin-2 OS=Rattus norvegicus GN=Phb2 PE=1 SV=1 | 0.57 | 1.4 | 11 | 7 | 2 | 8 | 3 | 3 | 6.67 | 4.67 | 2.60 | 1.67 |
| Cluster of sp\|P17182\|ENOA_MOUSE Alpha-enolase OS=Mus musculus GN=Eno1 PE=1 SV=3 | 0.59 | 1.4 | 30 | 8 | 5 | 14 | 10 | 6 | 14.51 | 10.25 | 7.95 | 2.30 |
| sp\|P80314\|TCPB_MOUSE T-complex protein 1 subunit beta OS=Mus musculus GN=Cct2 PE=1 SV=4 | 0.6 | 0.8 | 5 | 0 | 0 | 4 | 0 | 0 | 1.67 | 1.33 | 1.67 | 1.33 |
| sp\|P81155\|VDAC2_RAT Voltage-dependent anion-selective channel protein 2 OS=Rattus norvegicus GN=Vdac2 PE=1 SV=2 | 0.6 | 1.4 | 7 | 0 | 0 | 3 | 1 | 1 | 2.33 | 1.67 | 2.33 | 0.67 |
| sp\|Q63507\|RL14_RAT 60S ribosomal protein L14 OS=Rattus norvegicus GN=Rpl14 PE=1 SV=3 | 0.6 | 1.4 | 4 | 2 | 1 | 1 | 2 | 2 | 2.33 | 1.67 | 0.88 | 0.33 |
| sp\|O77768\|HNRPC_RABIT Heterogeneous nuclear ribonucleoprotein C OS=Oryctolagus cuniculus GN=HNRNPC PE=2 SV=1 | 0.6 | 1.4 | 7 | 0 | 0 | 3 | 2 | 0 | 2.33 | 1.67 | 2.33 | 0.88 |
| sp\|Q61171\|PRDX2_MOUSE Peroxiredoxin-2 OS=Mus musculus GN=Prdx2 PE=1 SV=3 | 0.6 | 1.5 | 4 | 1 | 1 | 3 | 0 | 1 | 2.00 | 1.33 | 1.00 | 0.88 |
| Cluster of sp\|O02691\|HCD2_BOVIN 3-hydroxyacyl-CoA dehydrogenase type-2 OS=Bos taurus GN=HSD17B10 PE=1 SV=3 | 0.61 | 1.5 | 2 | 0 | 2 | 2 | 1 | 0 | 1.39 | 0.91 | 0.73 | 0.50 |
| sp\|O35381\|AN32A_MOUSE Acidic leucine-rich nuclear phosphoprotein 32 family member A OS=Mus musculus GN=Anp32a PE=1 SV=1 | 0.61 | 1.3 | 4 | 0 | 0 | 1 | 2 | 0 | 1.33 | 1.00 | 1.33 | 0.58 |
| sp\|P21291\|CSRP1_HUMAN Cysteine and glycine-rich protein 1 OS=Homo sapiens GN=CSRP1 PE=1 SV=3 | 0.61 | 1.3 | 2 | 1 | 1 | 1 | 1 | 1 | 1.33 | 1.00 | 0.33 | 0.00 |
| Cluster of sp\|M0RC99\|RAB5A_RAT Ras-related protein Rab-5A OS=Rattus norvegicus GN=Rab5a PE=2 SV=1 | 0.61 | 1.5 | 4 | 0 | 0 | 3 | 0 | 0 | 1.33 | 0.91 | 1.33 | 0.91 |
| sp\|H9BW96\|RAB7_EPICO Ras-related protein rab7 OS=Epinephelus coioides GN=rab7 PE=1 SV=1 | 0.61 | 1.3 | 4 | 0 | 0 | 2 | 0 | 1 | 1.33 | 1.00 | 1.33 | 0.58 |
| sp\|P35268\|RL22_HUMAN 60S ribosomal protein L22 OS=Homo sapiens GN=RPL22 PE=1 SV=2 | 0.61 | 1.3 | 2 | 1 | 1 | 1 | 1 | 1 | 1.33 | 1.00 | 0.33 | 0.00 |
| sp\|P80316\|TCPE_MOUSE T-complex protein 1 subunit epsilon OS=Mus musculus GN=Cct5 PE=1 SV=1 | 0.62 | 0.7 | 3 | 0 | 0 | 1 | 0 | 1 | 1.00 | 0.67 | 1.00 | 0.33 |
| sp\|Q2TBU9\|RUVB2_BOVIN RuvB-like 2 OS=Bos taurus GN=RUVBL2 PE=2 SV=3 | 0.62 | 0.7 | 3 | 0 | 0 | 2 | 0 | 0 | 1.00 | 0.67 | 1.00 | 0.67 |
| sp\|P80313\|TCPH_MOUSE T-complex protein 1 subunit eta OS=Mus musculus GN=Cct7 PE=1 SV=1 | 0.62 | 0.7 | 3 | 0 | 0 | 2 | 0 | 0 | 1.00 | 0.67 | 1.00 | 0.67 |
| sp\|P47738\|ALDH2_MOUSE Aldehyde dehydrogenase, mitochondrial OS=Mus musculus GN=Aldh2 PE=1 SV=1 | 0.62 | 0.7 | 3 | 0 | 0 | 2 | 0 | 0 | 1.00 | 0.67 | 1.00 | 0.67 |
| Cluster of sp\|P18669\|PGAM1_HUMAN Phosphoglycerate mutase 1 OS=Homo sapiens GN=PGAM1 PE=1 SV=2 | 0.64 | 1.3 | 7 | 2 | 0 | 4 | 3 | 0 | 2.86 | 2.24 | 2.00 | 1.16 |
| sp\|O08709\|PRDX6_MOUSE Peroxiredoxin-6 OS=Mus musculus GN=Prdx6 PE=1 SV=3 | 0.66 | 1.5 | 3 | 0 | 0 | 2 | 0 | 0 | 1.00 | 0.67 | 1.00 | 0.67 |
| sp\|P13471\|RS14_RAT 40S ribosomal protein S14 OS=Rattus norvegicus GN=Rps14 PE=2 SV=3 | 0.66 | 1.5 | 3 | 0 | 0 | 2 | 0 | 0 | 1.00 | 0.67 | 1.00 | 0.67 |
| sp\|P22087\|FBRL_HUMAN rRNA 2'-O-methyltransferase fibrillarin OS=Homo sapiens GN=FBL PE=1 SV=2 | 0.66 | 1.5 | 3 | 0 | 0 | 2 | 0 | 0 | 1.00 | 0.67 | 1.00 | 0.67 |
| sp\|Q9Z2U0\|PSA7_MOUSE Proteasome subunit alpha type-7 OS=Mus musculus GN=Psma7 PE=1 SV=1 | 0.66 | 1.5 | 3 | 0 | 0 | 1 | 1 | 0 | 1.00 | 0.67 | 1.00 | 0.33 |
| sp\|P13272\|UCRI_BOVIN Cytochrome b-c1 complex subunit Rieske, mitochondrial OS=Bos taurus GN=UQCRFS1 PE=1 SV=3 | 0.66 | 1.5 | 3 | 0 | 0 | 2 | 0 | 0 | 1.00 | 0.67 | 1.00 | 0.67 |
| sp\|O60504\|VINEX_HUMAN Vinexin OS=Homo sapiens GN=SORBS3 PE=1 SV=2 | 0.66 | 1.5 | 0 | 1 | 2 | 0 | 2 | 0 | 1.00 | 0.67 | 0.58 | 0.67 |
| Cluster of sp\|P62143\|PP1B_RABIT Serine/threonine-protein phosphatase PP1-beta catalytic subunit OS=Oryctolagus cuniculus GN=PPP1CB PE=1 SV=3 | 0.71 | 1.5 | 6 | 0 | 0 | 4 | 0 | 0 | 1.88 | 1.23 | 1.88 | 1.23 |
